# Providing insight into the mechanism of action of cationic lipidated oligomers using metabolomics

**DOI:** 10.1128/msystems.00093-24

**Published:** 2024-04-12

**Authors:** Maytham Hussein, Muhammad Bilal Hassan Mahboob, Jessica R. Tait, James L. Grace, Véronique Montembault, Laurent Fontaine, John F. Quinn, Tony Velkov, Michael R. Whittaker, Cornelia B. Landersdorfer

**Affiliations:** 1 Department of Biochemistry and Pharmacology, School of Biomedical Sciences, Faculty of Medicine, Dentistry and Health Sciences, The University of Melbourne, Parkville, Victoria, Australia; 2 Department of Pharmacology, Monash Biomedicine Discovery Institute, Monash University, Clayton, Victoria, Australia; 3 Drug Delivery, Disposition, and Dynamics Theme, Monash Institute of Pharmaceutical Sciences, Monash University, Parkville, Victoria, Australia; 4 Institut des Molécules et Matériaux du Mans, UMR 6283 CNRS–Le Mans Université, Le Mans, France; 5 Department of Chemical and Biological Engineering, Faculty of Engineering, Monash University, Clayton, Victoria, Australia; University of California, Berkeley, Berkeley, California, USA

**Keywords:** antimicrobial resistance, peptidoglycan biosynthesis, cationic lipidated oligomers, antimicrobial peptides, antimicrobial polymer, metabolomics, methicillin-resistant *Staphylococcus aureus*

## Abstract

The increasing resistance of clinically relevant microbes against current commercially available antimicrobials underpins the urgent need for alternative and novel treatment strategies. Cationic lipidated oligomers (CLOs) are innovative alternatives to antimicrobial peptides and have reported antimicrobial potential. An understanding of their antimicrobial mechanism of action is required to rationally design future treatment strategies for CLOs, either in monotherapy or synergistic combinations. In the present study, metabolomics was used to investigate the potential metabolic pathways involved in the mechanisms of antibacterial activity of one CLO, C_12_-o-(BG-D)-10, which we have previously shown to be effective against methicillin-resistant *Staphylococcus aureus* (MRSA) ATCC 43300. The metabolomes of MRSA ATCC 43300 at 1, 3, and 6 h following treatment with C_12_-o-(BG-D)-10 (48 µg/mL, i.e., 3× MIC) were compared to those of the untreated controls. Our findings reveal that the studied CLO, C_12_-o-(BG-D)-10, disorganized the bacterial membrane as the first step toward its antimicrobial effect, as evidenced by marked perturbations in the bacterial membrane lipids and peptidoglycan biosynthesis observed at early time points, i.e., 1 and 3 h. Central carbon metabolism and the biosynthesis of DNA, RNA, and arginine were also vigorously perturbed, mainly at early time points. Moreover, bacterial cells were under osmotic and oxidative stress across all time points, as evident by perturbations of trehalose biosynthesis and pentose phosphate shunt. Overall, this metabolomics study has, for the first time, revealed that the antimicrobial action of C_12_-o-(BG-D)-10 may potentially stem from the dysregulation of multiple metabolic pathways.

**IMPORTANCE:**

Antimicrobial resistance poses a significant challenge to healthcare systems worldwide. Novel anti-infective therapeutics are urgently needed to combat drug-resistant microorganisms. Cationic lipidated oligomers (CLOs) show promise as new antibacterial agents against Gram-positive pathogens like methicillin-resistant *Staphylococcus aureus* (MRSA). Understanding their molecular mechanism(s) of antimicrobial action may help design synergistic CLO treatments along with monotherapy. Here, we describe the first metabolomics study to investigate the killing mechanism(s) of CLOs against MRSA. The results of our study indicate that the CLO, C_12_-o-(BG-D)-10, had a notable impact on the biosynthesis and organization of the bacterial cell envelope. C_12_-o-(BG-D)-10 also inhibits arginine, histidine, central carbon metabolism, and trehalose production, adding to its antibacterial characteristics. This work illuminates the unique mechanism of action of C_12_-o-(BG-D)-10 and opens an avenue to design innovative antibacterial oligomers/polymers for future clinical applications.

## INTRODUCTION

Antimicrobial resistance (AMR) represents a pressing global health challenge, posing significant threats to the successful treatment of infections and patient outcomes (1). Common mechanisms through which microbes develop resistance to antimicrobials include reduced drug uptake, drug target modification, and modification of drug and drug efflux; these may emerge due to genetic mutations or horizontal gene transfer (2). The emergence of AMR is largely attributed to the inappropriate and excessive use of various antibacterial agents, both within the healthcare sector and the agricultural industry (3). The dwindling arsenal of effective treatment options poses a substantial challenge, jeopardizing our ability to combat infections successfully. As a direct consequence, mortality rates are rising, as once-treatable infections become increasingly difficult to treat. Beyond the immediate health impacts, AMR causes a substantial economic burden through heightened healthcare costs, including due to prolonged hospital stays, higher likelihood of readmission, and increased expenditure on additional treatments (4). Recognizing AMR as a critical issue, global health organizations, such as the Infectious Diseases Society of America in 2009, the World Health Organization (WHO) in 2017, and the Centers for Disease Control and Prevention (US-CDC) of the United States in 2019, have emphasized the need for multifaceted strategies, including prudent antimicrobial use, surveillance, and the development of novel drugs. They also issued lists of critical pathogens on which to focus the research and development of new antimicrobials (5–8).

While conventional antimicrobials are currently used for treating infections, the rapid increase in AMR suggests the need for alternative treatments (9). Antimicrobial peptides (AMPs) have been investigated and have been demonstrated to be very effective in the killing of microbes (10–12). However, AMPs have limitations, such as instability, toxicity, and high production costs (13, 14). Recently, synthetic analogs of AMPs, i.e., antimicrobial polymers that mimic the structural features (cationic and hydrophobic moieties) of naturally occurring AMPs, have been designed and shown to overcome AMP limitations in *in vitro* studies and *in vivo* mouse models (15). This class of molecules has several advantages, such as broad-spectrum antimicrobial activities, a low tendency for resistance development, and a rapid bactericidal effect (11, 16–22). Furthermore, antimicrobial polymers offer extra advantages compared to AMPs in terms of stability, durability, and ease of large-scale production (17). These antimicrobial polymers have a broad range of mechanisms of action from membrane disruption to intracellular target inhibition, depending on the structural features (17). However, the full mechanism(s) of action of antimicrobial polymers remains unclear in the literature as detailed structure-property relationships are difficult to elucidate.

*Staphylococcus aureus* is a Gram-positive bacterial species categorized as a high-priority pathogen by the WHO and a serious threat by the US-CDC (23, 24). *S. aureus* is part of the normal flora of humans and usually does not cause infection while on the skin. However, when the skin barrier is damaged, *S. aureus* may enter the underlying tissue or bloodstream and cause a wide variety of infections (25). Currently, methicillin-resistant *S. aureus* (MRSA) is one of the main nosocomial pathogens and is prevalent in hospital settings (26). In 2019, 473,000 deaths were associated with MRSA infections globally (27). In Australia, MRSA infections were associated with increased inpatient mortality, as well as greater expense and longer hospital length of stay compared with methicillin-susceptible *S. aureus* (28).

In recent work, our group has shown that cationic lipidated oligomers (CLOs) demonstrate structure-dependent antimicrobial activity against both Gram-positive and Gram-negative bacteria and fungi (29). Interestingly, while these CLOs have been designed with the same structural features (with cationic residues and lipid tail) as the clinically used AMP colistin (30), they appear to have wider applicability. The synthesized CLOs have repeating cationic residues (e.g., tertiary amine, primary amine [mimicking lysine], guanidine [mimicking arginine], or imidazole [mimicking histidine]), which help these CLOs to electrostatically bind to the negatively charged bacterial membranes (17). The lipid tail in each of the CLOs helps to disrupt bacterial membranes more effectively (16). One CLO in particular, i.e., C_12_-o-(BG-D)-10, with 10 cationic guanidine residues and a C_12_ hydrocarbon lipid tail, exhibited marked antimicrobial activity against MRSA (29). These guanidine groups have been reported to form multidentate hydrogen bonds with sulfate and phosphate heads on the bacterial anionic membranes, leading to efficient bacterial membrane integration (17, 31, 32). However, their precise mechanism(s) of antimicrobial action remains largely unknown to date.

Metabolomics has emerged as a critical tool for the elucidation of the mechanisms of action of AMPs such as polymyxins (33, 34). In this study, we shine a light on the mechanism(s) of bacterial killing by the antimicrobial CLO, C_12_-o-(BG-D)-10, against MRSA strain ATCC 43300 using untargeted metabolomics.

## RESULTS AND DISCUSSION

The antibacterial activity of C_12_-o-(BG-D)-10 was previously assessed against *S. aureus* ATCC 43300 and demonstrated an appreciable activity with an MIC of 8–16 µg/mL (29). Additionally, C_12_-o-(BG-D)-10 exhibited an interesting mechanism of action when comparing dose-dependent membrane disruption (via fluorescence propidium iodide [PI] assay) and growth inhibition (Fig. S1). Specifically, C_12_-o-(BG-D)-10 exhibited only minor interaction with the bacterial membrane, with only ~30% membrane damage observed (relative to a melittin control) at the highest concentration tested. These results suggested that there could be potentially a secondary mechanism contributing to the observed antimicrobial activity for C_12_-o-(BG-D)-10 (29). To interrogate this complex mode of action, a metabolomics study was performed using an initial inoculum of 10^8^ colony-forming units (CFU)/mL with samples at 1, 3, and 6 h. The 48 µg/mL (3× MIC) C_12_-o-(BG-D)-10 concentration provided maximal bacterial killing at 1 h with ~1.5 log_10_CFU/mL decrease compared to the control (Fig. S2a). Metabolomics results of different perturbed metabolic pathways of MRSA are discussed below.

### Multivariate and univariate analysis

A total of 1,578 putative metabolites were identified under all treatment conditions. Out of these, 33% were not mapped to known metabolic pathways and 67% were mapped to known metabolic pathways according to common databases, e.g., PseudoCyc, MetaCyc, and LipidMaps databases. Most of the acquired metabolites belonged to lipid (18%), peptide (18%), and amino acid (17%) metabolism, while the minority of metabolites belonged to carbohydrate (6%), nucleotide (4%), secondary metabolites (2%), energy (1%), and glycan (0.5%) metabolite classes. The same databases were then used to designate metabolic classes or map the unmapped metabolites. Univariate data analysis was performed using two-sample *t*-tests (log_2_ fold change [FC] ≥ 0.59 or ≤−0.59, corresponding to a metabolite-level change of approximately 1.5-fold; false discovery rate [FDR] adjusted *P*-value ≤ 0.05) to determine significantly perturbed metabolites across all time points (1, 3, and 6 h) (Table S1). This analysis identified ~476 significantly perturbed metabolites (295, 241, and 185 at 1, 3, and 6 h, respectively) (Fig. S3). Across all time points, there were 53 overlapping metabolites with 155, 72, and 57 unique metabolites at 1, 3, and 6 h, respectively (Fig. S3). The majority of these metabolites were diminished in response to treatment with C_12_-o-(BG-D)-10. The heatmap showed that the intensities of metabolites varied after treatment with C_12_-o-(BG-D)-10 across all time points, especially at 1 h (Fig. S4). The reproducibility for all sample groups was acceptable across all time points (1, 3, and 6 h), where the median relative standard deviations (RSDs) across all time points were 14%–16% for untreated (control) groups, 17%–22% for treated samples, and 12% for the quality control (QC) group, consistent with some baseline variability in the dynamics of ordinary bacterial metabolism with and without C_12_-o-(BG-D)-10 treatment (Table 1). The well-separated treatment and control groups in the principal component analysis (PCA) revealed that C_12_-o-(BG-D)-10 treatment altered the metabolomic profile of MRSA across all time points (Fig. S5). The classification of the significantly impacted metabolites across all time points revealed that the lipids, peptides, amino acids, and carbohydrate (including glycans) metabolites were largely impacted, while the nucleotide metabolites and energy metabolites were less significantly perturbed across all the time points (Fig. S6).

**TABLE 1:** Median RSDs of all the metabolites of MRSA before and after treatment with C_12_-o-(BG-D)-10 across all the time points, i.e., 1, 3, and 6 h

	Median RSD %			
	1 h	3 h	6 h	QC
Control (untreated)	14	16	16	12
Treatment C_12_-o-(BG-D)-10	22	22	17	

### Pathway enrichment analysis for the significantly perturbed metabolites

C_12_-o-(BG-D)-10 treatment induced extensive perturbations in the metabolomic profile of MRSA ATCC 43300 across all time points. Consequently, therefore, we mapped and analyzed the significantly impacted metabolic features across all time points (i.e., 1, 3, and 6 h). The mapping of the significantly perturbed features of MRSA revealed that glycerophospholipids and fatty acids (FAs) metabolism, peptidoglycan and teichoic acid biosynthesis, DNA and RNA biosynthesis/nucleotide biosynthesis, central carbon metabolism, arginine biosynthesis, histidine metabolism, and pantothenate and co-enzyme A (CoA) biosynthesis were among the most significantly perturbed pathways (Table S2).

### Glycerophospholipid and fatty acid metabolism

**Fig 1:**
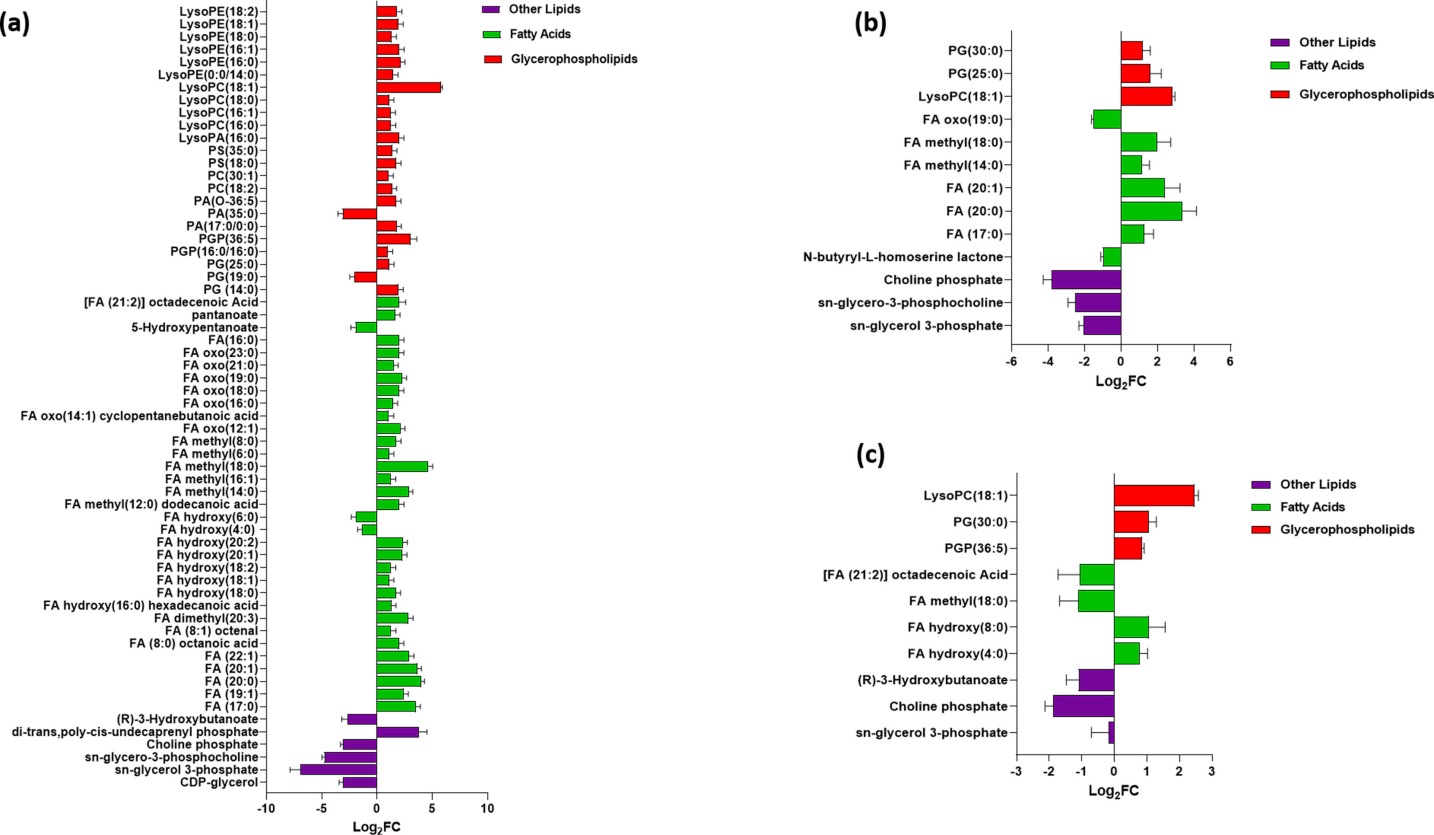
Significantly impacted lipids in MRSA ATCC 43300 following treatment with C_12_-o-(BG-D)10 at 1 h (**a**), 3 h (**b**), and 6 h (**c**). Putative lipid names are assigned based on accurate mass (≥1.0-log_2_-FC; *P* < 0.05). PE, phosphoethanolamines; PG, glycerophosphoglycerols; PS, glycerophosphoserines; PC, glycerophosphocholines; PA, glycerophosphates; PI, glycerophosphoinositols; PGP, glycerophosphoglycerophosphates; LysoPE, lysophosphatidylethanolamines; LysoPA, lysophosphatidic acid; LysoPC, lysophosphatidylcholines; and FA, fatty acids.

Following C_12_-o-(BG-D)-10 treatment, lipids were significantly perturbed, particularly the glycerophospholipids and fatty acid classes compared to other classes, i.e., glycerolipids, sphingolipids, and sterol lipids. Among glycerophospholipids, long chain glycerophosphoglycerols, including PG(25:0), PG(19:0), and PG(14:0), were largely perturbed at 1 h (Fig. 1a). The abundance of a highly important metabolite involved in the synthesis of bacterial membrane lipids and known precursor of teichoic acid in the Gram-positive bacterial cell wall, CDP-glycerol, was significantly decreased (log_2_FC = −3.01) at 1 h (Fig. 1a) (35). The levels of two more well-known metabolites involved in bacterial membrane lipids biosynthesis, *sn*-glycerol 3-phosphate and *sn*-glycero-3-phosphocholine, were substantially decreased at 1 h post-C_12_-o-(BG-D)-10 treatment (log_2_FC = −6.69 and −4.73, respectively) (Fig. 1a) (36–38). Also, di-trans,poly-cis-undecaprenyl phosphate (also known as lipid-P, bactoprenol, and C55-P) was significantly perturbed (log_2_FC = 3.9) at 1 h; lipid-P is involved in transporting peptidoglycan subunits from the cytoplasmic face of the cell membrane through the periplasmic space to the extracellular surface and plays a role in the synthesis of teichoic acid of the bacterial cell envelope (35, 39–42). Significant perturbations were also evident in the level of choline phosphate (log_2_FC = −3.0), which has a crucial role in the biosynthesis of wall teichoic acid in bacteria (43). The abundance of (R)-3-hydroxybutanoate, an essential precursor involved in the synthesis of the bacterial membrane, was significantly decreased at 1 h (log_2_FC = −2.6) (Fig. 1a) (44).

At 3 h, the impact of the C_12_-o-(BG-D)-10 on the glycerophospholipid levels continued, as manifested by significant decreases of *sn*-glycerol 3-phosphate (log_2_FC = −2.02) and *sn*-glycero-3-phosphocholine (log_2_FC = −2.4), albeit less than at 1 h (Fig. 1b). CDP-glycerol was not detected in the later time points, i.e., 3 and 6 h. Choline phosphate (log_2_FC = −3.7) was further significantly reduced. Notably, abundance of glycerophospholipids and fatty acids, including PG(25:0), PG(30:0), FA (17:0), FA (20:0), FA (20:1), FA methyl(14:0), FA methyl(18:0), and LysoPC(18:1), was significantly increased (≥1-log_2_FC, *P* < 0.05) except for FA oxo(19:0) (≥−1-log_2_FC, *P* < 0.05). Importantly, the abundance of *N*-butyryl-L-homoserine lactone, a quorum-sensing signaling molecule, was markedly decreased (log_2_FC = −1.00) in response to C_12_-o-(BG-D)-10 treatment (Fig. 1b) (45).

At 6 h, the level of *sn*-glycero-3-phosphocholine decreased moderately (log_2_FC = −0.68), while *sn*-glycerol 3-phosphate was completely diminished and was not detected. The level of choline phosphate (log_2_FC = −1.8) was reduced. Most fatty acids and phosphatidylglycerol phosphate levels were further increased, including PG (30:0), FA hydroxy (4:0), FA hydroxy (8:0), LysoPC (18:1), and PGP (36:5) (≥1-log_2_FC, *P* < 0.05). The level of (R)-3-hydroxybutanoate was further significantly decreased at 6 h (log_2_FC = −1.06). Also, the levels of [FA (21:2)] octadecenoic acid and FA methyl (18:0) were reduced (≥−1-log_2_FC, *P* < 0.05) (Fig. 1c).

Given that a higher number of significant perturbations of the lipid bilayer was observed at 1 h compared to 3 and 6 h, this suggests that C_12_-o-(BG-D)-10 disrupts the lipid bacterial membrane as the first step of the bacterial killing mechanism.

### Amino-sugar and sugar-nucleotide metabolism, peptidoglycan and teichoic acid biosynthesis

**Fig 2:**
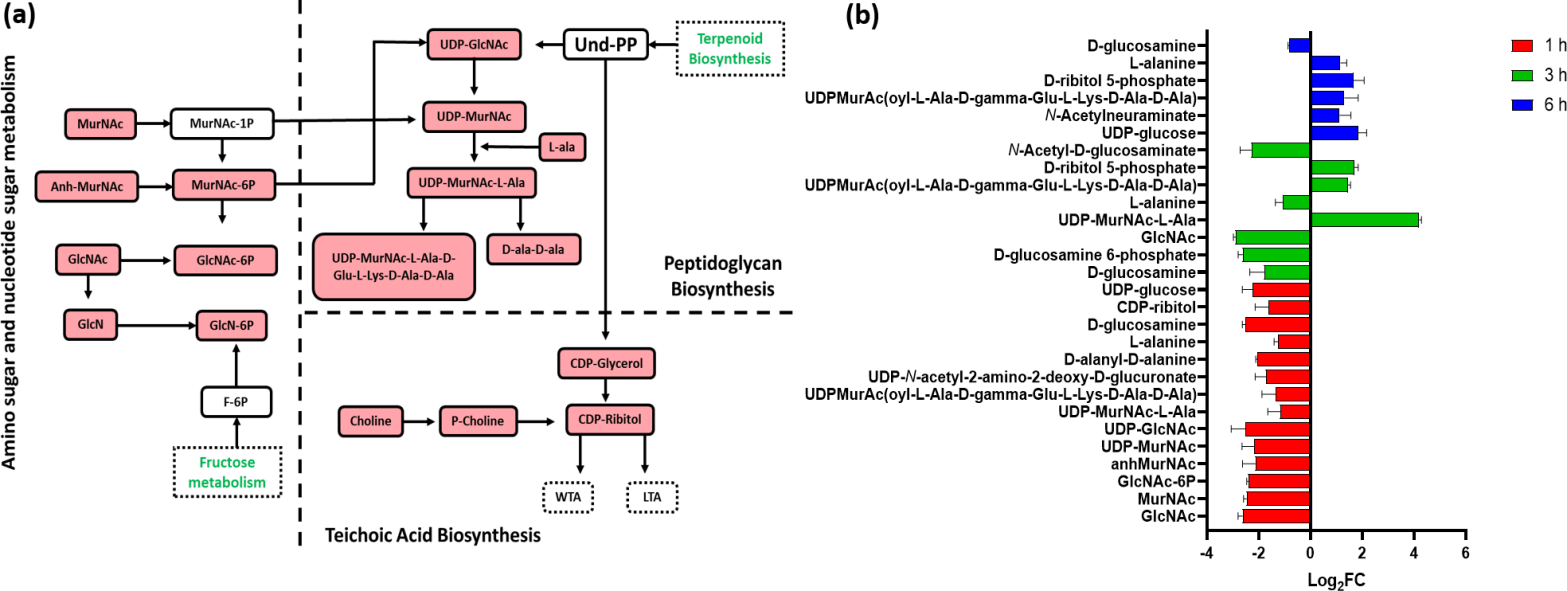
(**a**) Diagrammatic representation of all significantly impacted (increased: blue, decreased: red) amino-sugar and sugar-nucleotide metabolites in MRSA ATCC 43300 following treatment with C_12_-o-(BG-D)-10. (**b**) Significantly impacted amino-sugar and sugar-nucleotides in MRSA ATCC 43300 following treatment with C_12_-o-(BG-D)-10 at 1 h (red), 3 h (green), and 6 h (blue). Putative metabolite names are assigned based on accurate mass (≥1.0-log_2_-FC; *P* < 0.05).

Sugar nucleotides and amino sugars are the building blocks of peptidoglycans, involved in the synthesis of bacterial cell walls and teichoic acid in Gram-positive bacteria (46). After C_12_-o-(BG-D)-10 treatment, sugar nucleotides and amino sugar metabolism showed substantial alterations, indicating downregulation of peptidoglycan biosynthesis across all time points (1, 3, and 6 h). At 1 h, the treated bacterial cells displayed significant perturbations of metabolites involved in the early stages of peptidoglycan and teichoic acid formation. In contrast, modest disturbances were observed at 6 h (Fig. 2a).

At 1 h after C_12_-o-(BG-D)-10 treatment, the levels of seven crucial precursors involved in the biosynthesis of bacterial cell walls exhibited a significant decline. These included *N*-acetyl-D-glucosaminate (GlcNAc), *N*-acetylmuramate (MurNAc), *N*-acetyl-D-glucosamine 6-phosphate (GlcNAc-6P), 1,6-anhydro-*N*-acetylmuramate (anhMurNAc), UDP-*N*-acetylmuramate (UDP-MurNAc), and UDP-*N*-acetyl-D-glucosamine (UDP-GlcNAc) (≥−2-log_2_FC, *P* < 0.05) (Fig. 2b). Likewise, the precursors of the downstream pathway, peptidoglycan, including D-alanyl-D-alanine, UDP-*N*-acetylmuramoyl-L-alanine (UDP-MurNAc-L-Ala), UDP-MurAc(oyl-L-Ala-D-gamma-Glu-L-Lys-D-Ala-D-Ala), UDP-*N*-acetyl-2-amino-2-deoxy-D-glucuronate, and L-alanine were also markedly reduced (≥−1-log_2_-FC, *P* < 0.05). Interestingly, C_12_-o-(BG-D)-10 caused a significant impact on the crucial elements of wall teichoic acid. Notably, it decreased the levels of key constituents namely MurNAc and GlcNAc, which also play integral roles in amino sugar metabolism, as well as the level of CDP-ribitol, a pivotal molecule of wall teichoic acid biogenesis (≥−1.0-log_2_FC, *P* < 0.05) (47). The level of choline involved in the synthesis of crucial intermediates of teichoic acid was also significantly reduced (log_2_FC = −3.32) (48). UDP-glucose, a nucleotide sugar that serves as a key intracellular intermediary in the biosynthesis of the bacterial cell envelope, was significantly lowered (log_2_FC = −2.1) at 1 h (Fig. 2b) (49).

At 3 h, further perturbations in the building blocks of peptidoglycan were observed, where the levels of amino and nucleotide sugar intermediates involved in the generation of peptidoglycan building blocks were significantly reduced. These included D-glucosamine 6-phosphate, *N*-acetyl-D-glucosamine, and GlcNAc, which were reduced (≥−2-log_2_FC, *P* < 0.05) (Fig. 2b). Additionally, the level of UDP-MurNAc-L-Ala and UDP-MurNAc(oyl-L-Ala-D-gamma-Glu-L-Lys-D-Ala-D-Ala) was increased (≥1-log_2_FC, *P* < 0.05), while the level of D-glucosamine and L-alanine was decreased (≥−1-log_2_FC, *P* < 0.05). The level of D-ribitol 5-phosphate was significantly increased (log_2_FC = 1.7), which has a crucial role in the synthesis of wall teichoic acid in the bacterial cell envelope (Fig. 2b) (40).

Contrary to the pronounced effects observed at earlier time points, 1 and 3 h, C_12_-o-(BG-D)-10 treatment caused a minor effect on sugar nucleotides and amino sugars and its downstream pathways (peptidoglycan and wall teichoic acid) at 6 h. Notably, there was a significant increase in the levels of UDP-glucose, N-acetylneuraminate, UDP-MurNAc(oyl-L-Ala-D-gamma-Glu-L-Lys-D-Ala-D-Ala), D-ribitol 5-phosphate, and L-alanine (≥1-log_2_FC, *P* < 0.05) (Fig. 2b).

Similar to the response patterns of glycerophospholipids and fatty acids, the amino and nucleotide sugars and metabolites involved in the biogenesis of peptidoglycan were more frequently perturbed at 1 h than at later time points.

### Histidine metabolism

**Fig 3:**
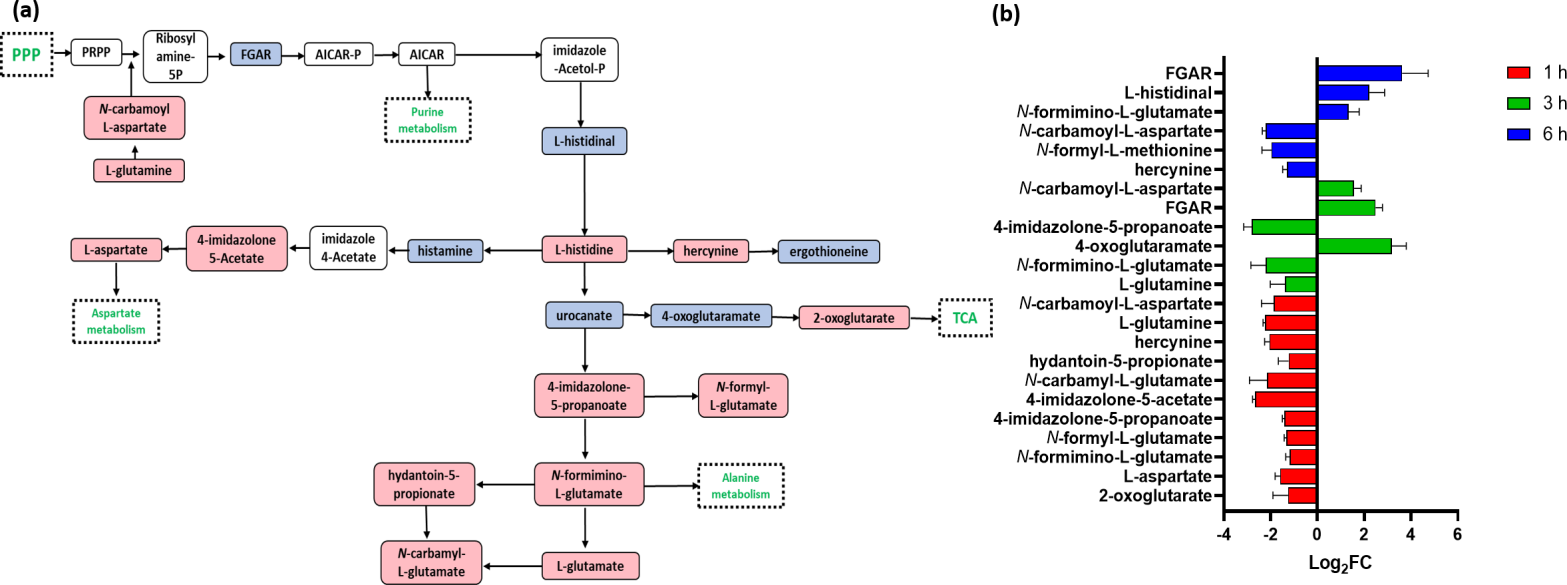
(**a**) Diagrammatic representation of all significantly impacted (increased: blue, decreased: red) histidine metabolites in MRSA ATCC 43300 following treatment with C_12_-o-(BG-D)-10. (**b**) Significantly impacted histidine metabolites in MRSA ATCC 43300 following treatment with C_12_-o-(BG-D)-10 at 1 h (red), 3 h (green), and 6 h (blue). Putative metabolite names are assigned based on accurate mass (≥1.0-log_2_-FC; *P* < 0.05).

The histidine metabolic pathway plays a crucial role in fundamental regulatory processes in bacteria, including amino acids, purines, and thiamine biosynthesis (50). The main metabolic intermediate generated by the histidine pathway is 5′-phosphoribosyl-4-carboxamide-5-aminoimidazole (AICAR). This intermediate is at the crossroads between purine-histidine cross talk (51). Therefore, the histidine pathway has been extensively investigated as a promising therapeutic target for novel antibiotics to treat infections caused by *Staphylococcus* (50, 52). Treatment with C_12_-o-(BG-D)-10 led to perturbation in several essential metabolites within the histidine biosynthetic pathway, notably including intermediates like L-glutamine and L-glutamate, both recognized as indicators of bacterial stress response (Fig. 3a) (53).

At 1 h post-C_12_-o-(BG-D)-10 treatment, 10 essential intermediates of histidine metabolism were significantly perturbed. Specifically, 4-imidazolone-5-acetate, *N*-carbamyl-L-glutamate, hydantoin-5-propionate, L-glutamine, and hercynine displayed more significant alteration (≥−2-log_2_FC, *P* < 0.05) compared to 4-imidazolone-5-propanoate, *N*-formyl-L-glutamate, *N*-formimino-L-glutamate, L-aspartate, and 2-oxoglutarate (≥−1-log_2_FC, *P* < 0.05) (Fig. 3b).

At 3 h, alterations were more pronounced than at 1 h. This was evident in decreased abundance of L-glutamate, L-glutamine, *N*-formimino L-glutamate, and 4-imidazolone-5-propanoate (≥−1-log_2_FC, *P* < 0.05), accompanied by an increased level of 4-oxoglutaramate (log_2_FC = 3.1). Importantly, the level of 5-phosphoribosyl-*N*-formylglycinamide (FGAR) (log_2_FC = 2.4) significantly increased, a key element in AICAR synthesis (Fig. 3b).

At 6 h, further significant perturbations were observed in the histidine biosynthetic pathway. FGAR, N-formimino-L-glutamate, and L-histidinal levels increased (≥1-log_2_FC, *P* < 0.05), while the levels of hercynine, *N*-formyl-L-methionine, and *N*-carbamoyl-L-aspartate decreased (≥−1-log_2_FC, *P* < 0.05) (Fig. 3b). These time-sensitive changes underscore a dynamic response of the histidine pathway to the treatment.

### Nucleotide (purine and pyrimidine) metabolism

**Fig 4:**
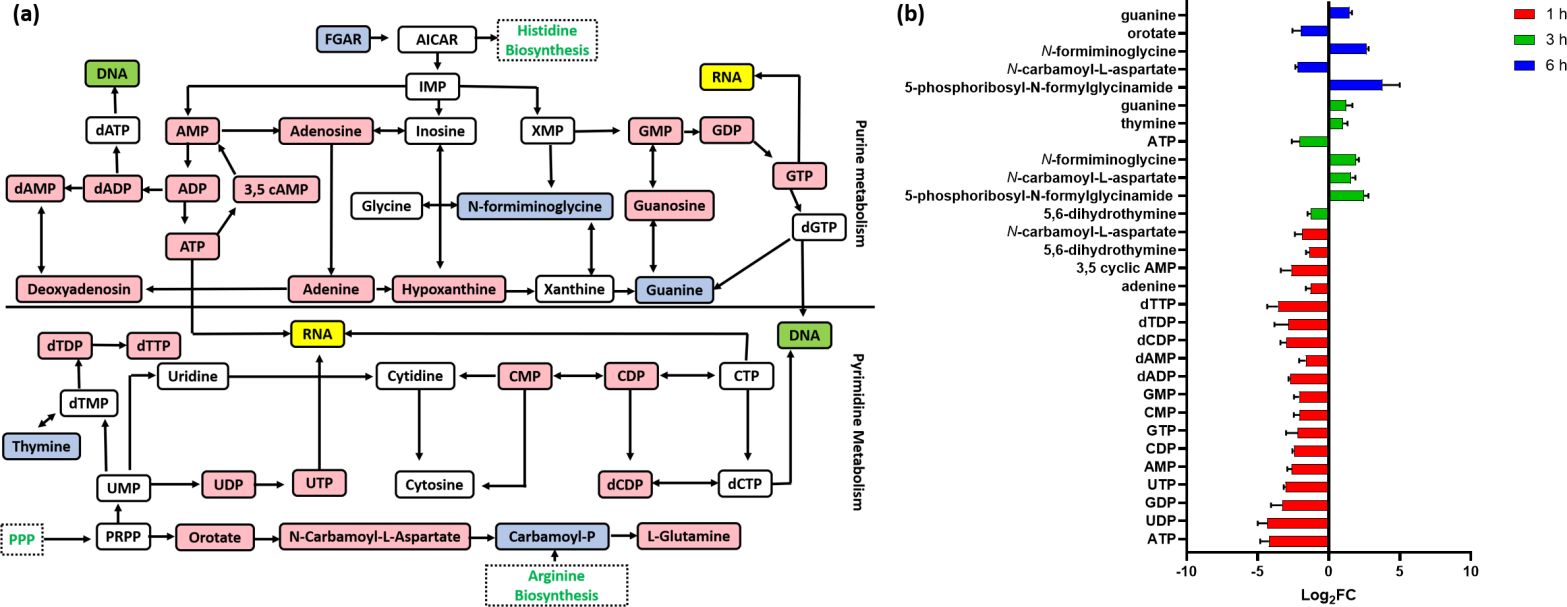
(**a**) Diagrammatic representation of all significantly impacted (increased: blue, decreased: red) pyrimidine and purine metabolites in MRSA ATCC 43300 following treatment with C_12_-o-(BG-D)-10. (**b**) Significantly impacted nucleotide and peptide metabolites in MRSA ATCC 43300 following treatment with C_12_-o-(BG-D)-10 at 1 h (red), 3 h (green), and 6 h (blue). Putative metabolite names are assigned based on accurate mass (≥1.0-log_2_-FC; *P* < 0.05).

C_12_-o-(BG-D)-10 treatment induced a marked dysregulation in the histidine interconnected pathway and nucleotide (purine and pyrimidine) metabolism, both are crucial for DNA and RNA formation (Fig. 4a) (54). This impact was more pronounced at 1 and 3 h compared to the later time point, 6 h (Fig. 4b).

At 1 h, a marked decline in the levels of 14 nucleotides was observed following C_12_-o-(BG-D)-10 treatment, including ATP, UTP, AMP, and dAMP (≥−1.5-log_2_FC, *P* < 0.05), which ultimately impacted DNA and RNA synthesis (Fig. 4b) (55, 56). Among nucleotide bases, adenine (log_2_FC = −1.2) was significantly reduced (Fig. 4b). An important observation at 1 h was that the level of 3,5 cyclic AMP required for cyclic di-AMP synthesis was profoundly reduced (log_2_FC = −2.7) (Fig. 4b). Cyclic di-AMP is an essential regulator for several bacterial biochemical processes, including metabolic pathways governing bacterial growth (fatty acid, carbohydrates, and nucleotide metabolism), virulence, biofilm formation, and cell cycle progression (57). It is also involved in bacterial cell survival mechanisms (58). The level of 5,6-dihydrothymine was reduced (log_2_FC = −1.6). Furthermore, the level of *N*-carbamoyl-L-aspartate, another key intermediate in the metabolism of purines and pyrimidines, was also decreased (log_2_FC = −1.91) (Fig. 4b) (59).

At 3 h, significant perturbations were observed in the levels of two pivotal intermediates of purine and pyrimidine metabolism. Specifically, the level of 5,6-dihydrothymine (log_2_FC = −1.2) exhibited a decrease, while the level of 5-phosphoribosyl-*N*-formylglycinamide (log_2_FC = 2.4) showed an increase (59, 60). Moreover, the levels of *N*-carbamoyl-L-aspartate and *N*-formiminoglycine, thymine, and guanine demonstrated increases (≥1.0-log_2_FC, *P* < 0.05) (Fig. 4b). However, the level of ATP, which is the major source of energy in the bacteria, experienced a further significant reduction (log_2_FC = −1.9) (61).

At 6 h, a notable increase was observed in the level of 5′-phosphoribosyl-*N*-formylglycinamide (log_2_FC = 3.7). Conversely, the *N*-carbamoyl-L-aspartate level experienced a further decline (log_2_FC = −2.2), while the level of *N*-formiminoglycine increased (log_2_FC = 2.6) (Fig. 4b). Orotate (log_2_FC = −1.9), an essential metabolite in the biosynthesis of pyrimidine, was significantly decreased at 6 h (Fig. 4b) (62). Among nucleotide bases, the level of guanine (log_2_FC = 1.4) was significantly increased (Fig. 4b).

### Central carbon metabolism

**Fig 5:**
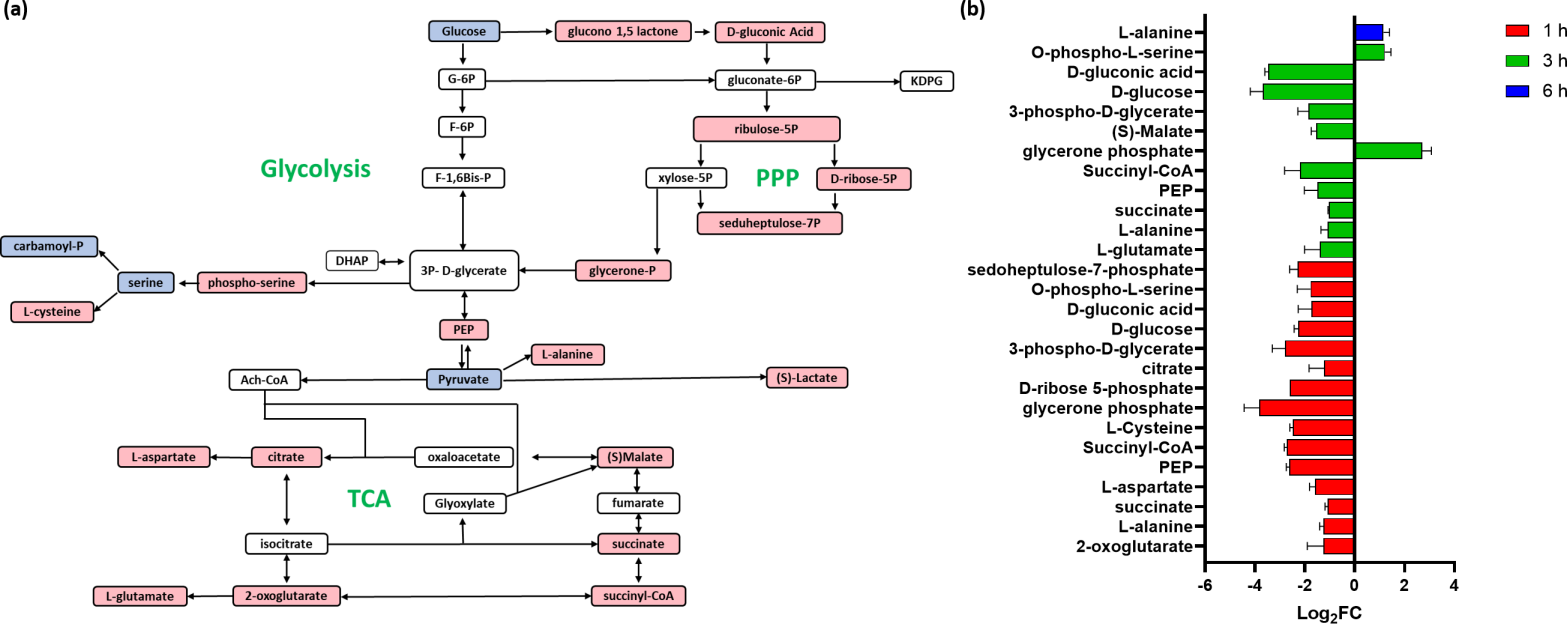
(**a**) Diagrammatic representation of all significantly impacted (increased: blue, decreased: red) central carbon metabolism metabolites in MRSA ATCC 43300 following treatment with C_12_-o-(BG-D)-10. (**b**) Significantly impacted central carbon metabolism metabolites in MRSA ATCC 43300 following treatment with C_12_-o-(BG-D)-10 at 1 h (red), 3 h (green), and 6 h (blue). Putative metabolite names are assigned based on accurate mass (≥1.0-log_2_-FC; *P* < 0.05).

Central carbon metabolism, which includes glycolysis, pentose phosphate pathway (PPP), and tricarboxylic acid cycle (TCA), is required for bacteria to generate energy in the form of ATP. This process provides precursors for all the biosynthetic reactions that are required for cell survival (63, 64). This makes central carbon metabolism, i.e., carbon uptake and carbon utilization, an attractive antimicrobial target because these processes are typically essential for microbial survival (65–67). Innate immunity, which frequently deprives bacteria of iron, amino acids, and other essential nutrients, serves as an example of the efficacy of this strategy (68–70). C_12_-o-(BG-D)-10 treatment caused perturbations in all three pathways of central carbon metabolism more significant at 1 h compared to later time points (Fig. 5a).

At 1 h, 18 important intermediates of central carbon metabolism, interlinking glycolysis, TCA, and PPP were significantly perturbed (Fig. 5b). This included, glycerone phosphate, phosphoenolpyruvate (PEP), succinyl-CoA, D-ribose 5-phosphate, 3-phospho-D-glycerate, L-cysteine, D-glucose, and sedoheptulose-7-phosphate, 2-oxoglutarate, L-alanine, succinate, L-aspartate, citrate, D-gluconic acid, and O-phospho-L-serine (≥−1-log_2_FC, *P* < 0.05) (Fig. 5b).

The effect of C_12_-o-(BG-D)-10 continued at 3 h, when D-glucose, D-gluconic acid, L-glutamate, L-alanine, succinate, PEP, (S)-malate, 3-phospho-D-glycerate, and succinyl-CoA (≥−1-log_2_FC, *P* < 0.05) were significantly perturbed. O-phospho-L-serine and glycerone phosphate (≥1-log_2_FC, *P* < 0.05) were also perturbed (Fig. 5b).

At 6 h, most of the metabolites of the central carbon metabolism were diminished and were not detected except for L-alanine, which was further significantly perturbed (log_2_FC = 1.15) (Fig. 5b).

Taken together, the crucial central carbon metabolism was largely and significantly perturbed at 1 and 3 h, while only minor perturbations were observed at 6 h.

### Arginine metabolism

**Fig 6:**
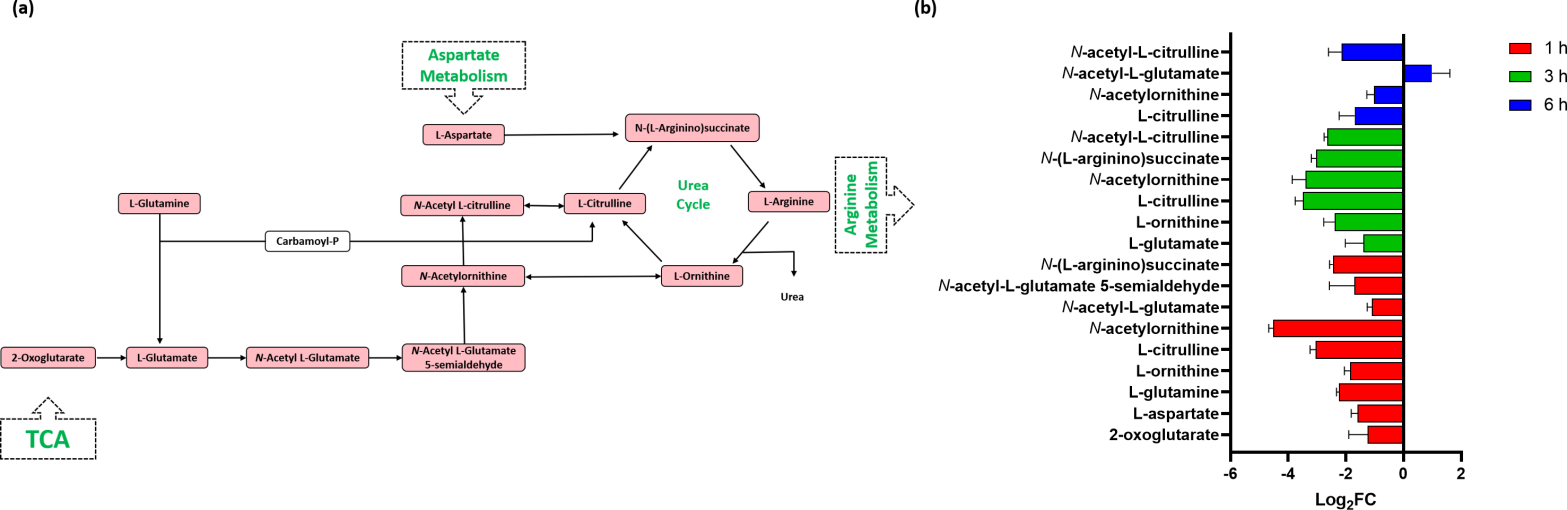
(**a**) Diagrammatic representation of all significantly impacted (increased: blue, decreased: red) arginine metabolism and interrelated TCA cycle metabolites in MRSA ATCC 43300 following treatment with C_12_-o-(BG-D)-10. (**b**) Significantly impacted metabolites in the arginine biosynthesis and metabolism in MRSA ATCC 43300 following treatment with C_12_-o-(BG-D)-10 at 1 h (red), 3 h (green), and 6 h (blue). Putative metabolite names are assigned based on accurate mass (≥1.0-log_2_-FC; *P* < 0.05).

The disruption of arginine (one of the most versatile and inter-convertible) metabolism has recently emerged as a powerful approach to control and subvert bacterial pathogenesis (71). C_12_-o-(BG-D)-10 treatment significantly impacted arginine metabolism, particularly at 1 and 3 h, with minimal changes observed after 6 h of treatment. (Fig. 6a).

After 1 h of treatment, nine metabolites of arginine biosynthesis, including L-aspartate, L-glutamine, L-citrulline, L-ornithine, *N*-acetyl-L-glutamate, *N*-(L-arginino) succinate, *N*-acetyl-L-glutamate 5-semialdehyde, 2-oxoglutarate, and *N*-acetylornithine, were significantly perturbed (≥−1-log_2_-FC, *P* < 0.05) (Fig. 6b).

At 3 h, the inhibitory impact of C_12_-o-(BG-D)-10 on arginine biosynthesis continued, although at an intensity wherein the levels of six important intermediates, including L-glutamine, L-ornithine, L-citrulline, *N*-(L-arginino) succinate, *N*-acetyl-L-citrulline, and *N*-acetylornithine, were significantly reduced (≥−1-log_2_-FC, *P* < 0.05) (Fig. 6b).

By 6 h, the level of *N*-acetyl-L-glutamate was increased (log_2_FC = 1.0), signifying a bacterial stress response. The levels of L-citrulline, *N*-acetylornithine, and *N*-acetyl-L-citrulline were decreased (≥−1-log_2_FC, *P* < 0.05) (Fig. 6b).

### Coenzyme A biosynthesis

Pantothenic acid, also known as vitamin B5, is essentially required by bacteria to synthesize coenzyme A (CoA). CoA, in turn, is crucial for the generation of fatty acids, carbohydrates, proteins, and even some intermediates within the TCA cycle (72). C_12_-o-(BG-D)-10 treatment resulted in marked disruption in the pantothenate and CoA biosynthesis (Fig. S7a).

At 1 h, C_12_-o-(BG-D)-10 treatment caused significant perturbations in the levels of five metabolites related to the pantothenate and CoA biosynthesis pathways (Fig. S7b). In particular, the levels of L-cysteine, CoA, L-aspartate, and 5,6-dihydrouracil significantly decreased (≥−1.0-log_2_FC, *P* < 0.05) (Fig. S7b). In contrast, uracil level increased (log_2_FC = 1.04). At 3 h, there was a notable decrease (≥−2-log2FC, *P* < 0.05) in the *N*-((R)-pantothenoyl)-L-cysteine and (R)−2,3-dihydroxy-3-methylbutanoate. Additionally, the level of pantothenate decreased (log_2_FC = −1.5), whereas 3-methyl-2-oxobutanoic acid increased (log_2_FC = 1.4) (Fig. S7b). The effect of C_12_-o-(BG-D)-10 persisted at 6 h, when the levels of pantetheine and pantothenate increased (≥1-log_2_FC, *P* < 0.05), while *N*-((R)-pantothenoyl)-L-cysteine demonstrated decreases (log_2_FC = −2.6) (Fig. S7b).

### Homeostasis and stress metabolism

C_12_-o-(BG-D)-10 treatment induced a significant perturbation in the metabolites responsible for bacterial homeostasis. D-glucose-6-phosphate, a substrate for trehalose-6-phosphate and UDP-glucose synthesis, experienced marked perturbations across all time points: at 1 h (log_2_FC = −1.8), 3 h (log_2_FC = −0.7), and 6 h (log_2_FC = 1.1) (Fig. S8) (73, 74). Trehalose, in its dephosphorylated form, serves as a recognized osmoprotectant in bacteria during osmotic stress (75). Significant alteration in the level of trehalose-6-phosphate at 3 h (log_2_FC = −1.71) indicated that the C_12_-o-(BG-D)-10 treatment induced osmotic stress. Choline, a precursor to glycine betaine, which acts as a potent osmoprotectant underwent significant reduction at 1 h (log_2_FC = −3.3) (76). However, choline was not detected at the later time points. Likewise, PPP intermediates involved in the maintenance of redox homeostasis in the cell were markedly perturbed, which was indicated by a significant decrease in the levels of ribose-5-phosphate, NADP+, and CMP (≥−1.5-log_2_FC, *P* < 0.05) at 1 h (Fig. S8) (77). However, these PPP intermediates were not detected at later time points.

### Conclusion

MRSA is a serious threat to global health and contributes to nearly half a million annual deaths. As further resistance emerges against current antimicrobials in clinical use, there is an urgent need for new treatment options. Our group has synthesized CLOs modeled on the structure of antimicrobial peptides. C_12_-o-(BG-D)-10 was previously found to inhibit the growth of MRSA *in vitro*; however, this activity was only partially attributable to membrane disruption as evident via fluorescence assay. The full mechanism(s) of its antimicrobial activity was not fully understood. This is the first study to investigate the mechanism(s) of antimicrobial action of a CLO, i.e., C_12_-o-(BG-D)-10 using metabolomics. C_12_-o-(BG-D)-10 antimicrobial action commences with disrupting the bacterial cell envelope. Early at 1 h, significant perturbations were observed in cell membrane lipids and glycerophospholipids, along with sugar nucleotides and amino-sugar metabolites linked to peptidoglycan and teichoic acid biosynthesis. The polymer also affected RNA and DNA biosynthesis and led to pronounced perturbations in histidine metabolism (linked to the synthesis of purines and pyrimidines), energy metabolism (i.e., arginine and TCA cycle), pantothenate biosynthesis, and CoA biogenesis (essentially required by cells for survival and normal growth). C_12_-o-(BG-D)-10 also perturbed central carbon metabolism and the stress pathway in the bacteria more prominently at the initial time points, i.e., 1 and 3 h. These insights on the mechanisms of action of C_12_-o-(BG-D)-10 will enable the rational design of antimicrobial combinations of clinically available antimicrobials with C_12_-o-(BG-D)-10 in future *in vitro* and *in vivo* studies in an approach to achieve synergistic and effective bacterial killing.

## MATERIALS AND METHODS

### CLO and antibiotic stock solution, media, and bacterial isolates

C_12_-o-(BG-D)-10 was synthesized by Cu(0)-mediated reversible deactivation radical polymerization using the protocol by Grace et al. (29). The CLO stock solutions were prepared by dissolving the CLO in DMSO first, diluting with MilliQ water (to 20% DMSO), and then vortexing until clear. DMSO was filtered through 0.22 µm sterile nylon filters before use. Methicillin-resistant *S. aureus* ATCC 43300 was used in the study. All susceptibility and time-kill studies were performed in cation-adjusted Mueller-Hinton broth (CAMHB; containing 20–25 mg/L Ca^2+^ and 10–12.5 mg/L Mg^2+^; BD, Sparks, MD, USA). Viable counting was performed on cation-adjusted Mueller–Hinton agar (CAMHA; containing 25 mg/L Ca^2+^ and 12.5 mg/L Mg^2+^; BD, Sparks, MD, USA).

### Antibacterial killing kinetics of CLO

A static concentration time-kill assay of C_12_-o-(BG-D)-10 against MRSA ATCC 43300 was performed (Fig. S2). Before the time-kill assay, MRSA ATCC 43300 was sub-cultured on a CAMHA plate and then incubated at 37°C for ~18–24 h. Three colonies were transferred from the CAMHA plate to inoculate 10 mL of sterile CAMHB in a 50 mL Falcon tube and incubated overnight in a shaking water bath (37°C, 150 rpm, ~16 h). The optical density of the bacterial suspension was measured using a spectrophotometer, and the suspension was appropriately diluted to achieve the targeted initial inoculum of ∼10^6^ CFU/mL (Fig. S2b). The inoculated tubes were dosed with C_12_-o-(BG-D)-10 (in 20% DMSO) to achieve concentrations of 8, 16, and 64 µg/mL. The DMSO concentrations in the tubes were ≤0.125%. One culture tube was drug-free as a control. At 0, 1.5, 5, 24, 48, and 72 h, 1 mL samples were collected from each tube, centrifuged, and washed twice with 0.9% normal saline. The samples were then serially diluted in saline plates and plated onto CAMHA plates. After 24-h incubation at 37°C, the CFU were counted, and the time-kill curves were graphed as log_10_ CFU/mL vs time (hours).

### Metabolomics sample preparation

An untargeted metabolomics study was carried out to explore the mechanism(s) of action of C_12_-o-(BG-D)-10 against MRSA ATCC 43300 using a concentration of 48 µg/mL (i.e., 3× MIC). Samples were taken and analyzed at the 1-, 3-, and 6-h time points in four biological replicates. An overnight culture was prepared by inoculating a single colony into 100 mL CAMHB in 250 mL conical flasks (Pyrex) and incubating the suspension in a shaker at 37°C and 180 rpm for ~16 h. After overnight incubation, log-phase cells were prepared in fresh MHB and then incubated for 2 h at 37°C at 180 rpm to the log phase with a starting bacterial inoculum of 10^8^ CFU/mL. Then, C_12_-o-(BG-D)-10 was added to obtain the desired concentration of 48 µg/mL (3× MIC), in parallel to a CLO-free control for each replicate. The flasks were then incubated at 37°C with shaking at 180 rpm. At each time point (0, 1, 3, and 6 h), 15 mL samples were transferred to 50 mL Falcon tubes for quenching, and the optical density reading at 600 nm (OD_600_) was then measured and normalized to the pre-treatment level of approximately ~0.5 with fresh CAMHB. Samples were then centrifuged at 3,220 × *g* and 4°C for 10 min, and the supernatants were removed. The pellets were stored at −80°C until metabolite extraction. The experiment was performed in four biological replicates to reduce the bias from inherent random variation.

### Metabolomics metabolite extraction

The bacterial pellets were washed twice in 1 mL of 0.9% saline and then centrifuged at 3,220 × *g* and 4°C for 5 min to remove residual extracellular metabolites and medium components. The washed pellets were resuspended in a cold extraction solvent (chloroform-methanol-water at 1:3:1, vol/vol) containing 1 µM each of the internal standards 3-[(3-cholamidopropyl)-dimethylammonio]−1-propanesulfonate, *N*-cyclohexyl-3-aminopropanesulfonic acid, piperazine-*N*, *N*-bis (2-ethanesulfonic acid), and Tris. The samples were then frozen in liquid nitrogen, thawed on ice, and vortexed to release the intracellular metabolites (three times). Next, the samples were transferred to 1.5 mL Eppendorf tubes and centrifuged at 14,000 *g* at 4°C for 10 min to remove any particulate matter. Finally, 200 µL of the supernatant was transferred into injection vials for liquid chromatography-mass spectrometry (LC-MS) analysis. An equal volume of each sample was combined and used as a QC sample.

To ensure that our metabolite extraction specifically targeted live-stressed bacterial cells, we performed a series of optimization steps, including a high inoculum time-kill assay, as illustrated in Fig. S2a. By this method, we determined that there was at least 10^6.5^ (i.e., 3 million) CFU/mL present in the samples, which equates to >45 million viable CFU per harvested sample. Furthermore, it is crucial to highlight that the mechanism of action of the polymer, as previously tested and confirmed in this study, demonstrates a membrane-damaging effect. This characteristic supports the assertion that the percentage of dead but intact cells in the pelleted samples is expected to be low.

### LC-MS analysis of metabolites

Both hydrophilic interaction liquid chromatography (HILIC) and reversed-phase liquid chromatography (RPLC) coupled with high-resolution mass spectrometry were employed to ensure the detection of both hydrophilic and hydrophobic metabolites. Samples were analyzed on a Dionex U3000 high-performance liquid chromatography system in tandem with a Q-Exactive Orbitrap mass spectrometer (Thermo Fisher) in both positive and negative ion modes with a resolution at 35,000. The HILIC method was described previously in detail (78). Briefly, samples maintained at 4°C were eluted through a ZIC-pHILIC column (5 µm, polymeric, 150 × 4.6 mm; SeQuant, Merck) by mobile phase A (20 mM ammonium carbonate) and mobile phase B (acetonitrile). The gradient started with 80% mobile phase B at a flow rate of 0.3 mL/min and was followed by a linear gradient to 50% mobile phase B over 15 min. The Ascentis Express C8 column (5 cm × 2.1 mm, 2.7 µm) (catalog no. 53,831-U; Sigma-Aldrich) was applied in the RPLC method. The samples were controlled at 4°C and eluted by mobile phase A (40% of isopropanol and 60% of Milli-Q water with 8 mM ammonium formate and 2 mM formic acid) and mobile phase B (98% of isopropanol and 2% of Milli-Q water with 8 mM ammonium formate and 2 mM formic acid). The linear gradient started from 100% mobile phase A to a final composition of 35% mobile phase A and 65% mobile phase B over 24 min at 0.2 mL/min. All samples were analyzed within a single LC-MS batch to avoid variations. The pooled quality control samples, internal standards, and total ion chromatograms were assessed to evaluate the chromatographic peaks, signal reproducibility, and stability of the analytes. To assist in the identification of metabolites, a mixture of ∼500 metabolite standards was analyzed within the same batch.

### Data processing, bioinformatics, and statistical analyses

IDEOM (Identification and Evaluation of Metabolites) was used to convert raw data obtained by LC-MS to annotated and hyperlinked metabolites (79). ProteoWizard, a freely available software library for LC-MS data analysis, was first used to extract mzXML files from LC-MS raw sheets. These files were then processed using XCMS, a graphical user interface, for peak picking and generating peakML files (80). Using MZmatch.R tool, peaks were aligned and filtered with minimum detectible intensity of 100,000 and RSD of <0.5 and peak shape (coda dw) of >0.8. Using the same MZmatch, the missing peaks were also retrieved and annotated. Common sources of noise (contaminant signals, peak shoulders, and irreproducible peaks) were removed using the IDEOM interface. The gain and loss of protons were corrected in positive and negative electrospray ionization mode and then a data-dependent mass recalibration (2 ppm) step for putative metabolites was performed. Metabolites confirmed with authentic standards were assigned with MSI level 1 identification. Putative metabolites (with MSI level 2 identification) were identified by comparing their accurate masses and retention times with the standards in the databases including KEGG (Kyoto Encyclopedia of Genes and Genomics), LipidMaps, MetaCyc, and preferably EcoCyc. Peak height intensities were used for the quantification of the metabolites. Statistical analysis was performed using MetaboAnalyst 5.0 (81), a freely available online statistical tool. Briefly, putative metabolites (with median RSD ≤ 20% and confidence interval ≥ 5) were extracted from IDEOM and tabled per time point, then uploaded on MetaboAnalyst 5.0. Data were filtered using the interquartile range, normalized by the median, log_2_ transformed, and autoscaled. Fold change was calculated relative to the control from the corresponding time point. Univariate analysis was performed using two-sample *t*-tests (log_2_FC ≥ 0.59 or ≤−0.59, corresponding to a metabolite level change of approximately 1.5-fold; FDR adjusted *P*-value ≤ 0.05) to determine significantly perturbed metabolites for each time point. Multivariate analysis was performed and included the generation of heat maps and PCA plots. Finally, the KEGG IDs of metabolites were uploaded to KEGG Mapper (82), and pathways were constructed. The individual value plots for the significantly perturbed metabolites after treatment with C_12_-o-(BG-D)-10 across all the time points can be found in the supplemental material (Fig. S9 to S14).

## Data Availability

The entirety of our raw data is available on Metabolomics Workbench under Study No. ST003053.

## SUPPLEMENTAL MATERIAL

Supplemental material msystems.00093-24-s0001.docx Supplemental figures and Table S2.

Table S1 msystems.00093-24-s0002.docx Metabolite IDs.

ASM does not own the copyrights to Supplemental Material that may be linked to, or accessed through, an article. The authors have granted ASM a non-exclusive, world-wide license to publish the Supplemental Material files. Please contact the corresponding author directly for reuse.

## References

[1] Hay SI, Rao PC, Dolecek C, Day NPJ, Stergachis A, Lopez AD, Murray CJL. 2018. Measuring and mapping the global burden of antimicrobial resistance. BMC Med 16:78. doi:10.1186/s12916-018-1073-z 29860943 PMC5985573

[2] Reygaert WC. 2018. An overview of the antimicrobial resistance mechanisms of bacteria. AIMS Microbiol 4:482–501. doi:10.3934/microbiol.2018.3.482 31294229 PMC6604941

[3] Dadgostar P. 2019. Antimicrobial resistance: implications and costs. Infect Drug Resist 12:3903–3910. doi:10.2147/IDR.S234610 31908502 PMC6929930

[4] Poudel AN, Zhu S, Cooper N, Little P, Tarrant C, Hickman M, Yao G. 2023. The economic burden of antibiotic resistance: a systematic review and meta-analysis. PLoS One 18:e0285170. doi:10.1371/journal.pone.0285170 37155660 PMC10166566

[5] Peterson LR. 2009. Bad bugs, no drugs: no ESCAPE revisited. Clin Infect Dis 49:992–993. doi:10.1086/605539 19694542

[6] Asokan GV, Ramadhan T, Ahmed E, Sanad H. 2019. WHO global priority pathogens list: a bibliometric analysis of Medline-PubMed for knowledge mobilization to infection prevention and control practices in Bahrain. Oman Med J 34:184–193. doi:10.5001/omj.2019.37 31110624 PMC6505350

[7] Centers for Disease Control Prevention (CDC). 2019. Antibiotic resistance threats in the United States, 2019. US Department of Health and Human Services, Centres for Disease Control and Prevention.

[8] Butler MS, Henderson IR, Capon RJ, Blaskovich MAT. 2023. Antibiotics in the clinical pipeline as of December 2022. J Antibiot 76:431–473. doi:10.1038/s41429-023-00629-8 PMC10248350 37291465

[9] Lewis K. 2020. At the crossroads of bioenergetics and antibiotic discovery. Biochemistry (Mosc) 85:1469–1483. doi:10.1134/S0006297920120019 33705287

[10] Erdem Büyükkiraz M, Kesmen Z. 2022. Antimicrobial peptides (AMPs): a promising class of antimicrobial compounds. J Appl Microbiol 132:1573–1596. doi:10.1111/jam.15314 34606679

[11] Ergene C, Yasuhara K, Palermo EF. 2018. Biomimetic antimicrobial polymers: recent advances in molecular design. Polym Chem 9:2407–2427. doi:10.1039/C8PY00012C

[12] Carmona-Ribeiro AM, Araújo PM. 2021. Antimicrobial polymer−based assemblies: a review. Int J Mol Sci 22:5424. doi:10.3390/ijms22115424 34063877 PMC8196616

[13] Zhu Y, Hao W, Wang X, Ouyang J, Deng X, Yu H, Wang Y. 2022. Antimicrobial peptides, conventional antibiotics, and their synergistic utility for the treatment of drug‐resistant infections. Med Res Rev 42:1377–1422. doi:10.1002/med.21879 34984699

[14] Qin H, Zuo W, Ge L, Siu SWI, Wang L, Chen X, Ma C, Chen T, Zhou M, Cao Z, Kwok HF. 2023. Discovery and analysis of a novel antimicrobial peptide B1AW from the skin secretion of Amolops wuyiensis and improving the membrane-binding affinity through the construction of the lysine-introduced analogue. Comput Struct Biotechnol J 21:2960–2972. doi:10.1016/j.csbj.2023.05.006 37228702 PMC10205438

[15] Zhou W, Shi G, Zhao P, Zhang G, Yang P, Li B, Li B, Wan X, Zheng Y. 2023. Dynamic helical cationic polyacetylenes for fast and highly efficient killing of bacteria. Acta Biomater 161:134–143. doi:10.1016/j.actbio.2023.02.017 36804537

[16] Si Z, Zheng W, Prananty D, Li J, Koh CH, Kang E-T, Pethe K, Chan-Park MB. 2022. Polymers as advanced antibacterial and antibiofilm agents for direct and combination therapies. Chem Sci 13:345–364. doi:10.1039/d1sc05835e 35126968 PMC8729810

[17] Pham P, Oliver S, Boyer C. 2023. Design of antimicrobial polymers. Macromol Chem Phys 224:2200226. doi:10.1002/macp.202200226

[18] Camacho-Cruz L, Velazco-Medel MA, Cruz-Gómez A, Bucio E. 2020. Antimicrobial polymers, p 1–42. In Advanced antimicrobial materials and applications. Springer.

[19] Thoma LM, Boles BR, Kuroda K. 2014. Cationic methacrylate polymers as topical antimicrobial agents against Staphylococcus aureus nasal colonization. Biomacromolecules 15:2933–2943. doi:10.1021/bm500557d 25010735 PMC4130249

[20] Lam SJ, O’Brien-Simpson NM, Pantarat N, Sulistio A, Wong EHH, Chen Y-Y, Lenzo JC, Holden JA, Blencowe A, Reynolds EC, Qiao GG. 2016. Combating multidrug-resistant Gram-negative bacteria with structurally nanoengineered antimicrobial peptide polymers. Nat Microbiol 1:16162. doi:10.1038/nmicrobiol.2016.162 27617798

[21] Nederberg F, Zhang Y, Tan JPK, Xu K, Wang H, Yang C, Gao S, Guo XD, Fukushima K, Li L, Hedrick JL, Yang Y-Y. 2011. Biodegradable nanostructures with selective lysis of microbial membranes. Nat Chem 3:409–414. doi:10.1038/nchem.1012 21505501

[22] Mizutani M, Palermo EF, Thoma LM, Satoh K, Kamigaito M, Kuroda K. 2012. Design and synthesis of self-degradable antibacterial polymers by simultaneous chain-and step-growth radical copolymerization. Biomacromolecules 13:1554–1563. doi:10.1021/bm300254s 22497522

[23] Beyer P, Paulin S. 2020. Priority pathogens and the antibiotic pipeline: an update. Bull World Health Organ 98:151. doi:10.2471/BLT.20.251751 32132745 PMC7047031

[24] Kadri SS. 2020. Key takeaways from the US CDC’s 2019 antibiotic resistance threats report for frontline providers. Crit Care Med 48:939–945. doi:10.1097/CCM.0000000000004371 32282351 PMC7176261

[25] Rasigade J-P, Vandenesch F. 2014. Staphylococcus aureus: a pathogen with still unresolved issues. Infect Genet Evol 21:510–514. doi:10.1016/j.meegid.2013.08.018 23994773

[26] Ferri M, Ranucci E, Romagnoli P, Giaccone V. 2017. Antimicrobial resistance: a global emerging threat to public health systems. Crit Rev Food Sci Nutr 57:2857–2876. doi:10.1080/10408398.2015.1077192 26464037

[27] Murray CJL, Ikuta KS, Sharara F, Swetschinski L, Robles Aguilar G, Gray A, Han C, Bisignano C, Rao P, Wool E, et al.. 2022. Global burden of bacterial antimicrobial resistance in 2019: a systematic analysis. The Lancet 399:629–655. doi:10.1016/S0140-6736(21)02724-0 PMC8841637 35065702

[28] Miyakis S, Brentnall S, Masso M, Reynolds G, Byrne MK, Newton P, Crawford S, Fish J, Nicholas B, Hill T, van Oijen AM, Wollongong Antimicrobial Resistance Research Alliance (WARRA) and One Health Understanding Through Bacterial Resistance to Antibiotics Knowledge (OUTBREAK) Consortium. 2022. Key predictors and burden of meticillin-resistant Staphylococcus aureus infection in comparison with meticillin-susceptible S. aureus infection in an Australian hospital setting. J Hosp Infect 129:41–48. doi:10.1016/j.jhin.2022.07.004 35839999

[29] Grace JL, Amado M, Reid JC, Elliott AG, Landersdorfer CB, Truong NP, Kempe K, Cooper MA, Davis TP, Montembault V, Pascual S, Fontaine L, Velkov T, Quinn JF, Whittaker MR. 2019. An optimised Cu(0)-RDRP approach for the synthesis of lipidated oligomeric vinyl azlactone: toward a versatile antimicrobial materials screening platform. J Mater Chem B 7:6796–6809. doi:10.1039/c9tb01624d 31603181

[30] El-Sayed Ahmed MAE-G, Zhong L-L, Shen C, Yang Y, Doi Y, Tian G-B. 2020. Colistin and its role in the era of antibiotic resistance: an extended review (2000–2019). Emerg Microbes Infect 9:868–885. doi:10.1080/22221751.2020.1754133 32284036 PMC7241451

[31] Chan DI, Prenner EJ, Vogel HJ. 2006. Tryptophan-and arginine-rich antimicrobial peptides: structures and mechanisms of action. Biochim Biophys Acta 1758:1184–1202. doi:10.1016/j.bbamem.2006.04.006 16756942

[32] Cutrona KJ, Kaufman BA, Figueroa DM, Elmore DE. 2015. Role of arginine and lysine in the antimicrobial mechanism of histone-derived antimicrobial peptides. FEBS Lett 589:3915–3920. doi:10.1016/j.febslet.2015.11.002 26555191 PMC4713009

[33] Hussein M, Han M-L, Zhu Y, Zhou Q, Lin Y-W, Hancock REW, Hoyer D, Creek DJ, Li J, Velkov T. 2019. Metabolomics study of the synergistic killing of polymyxin B in combination with amikacin against polymyxin-susceptible and-resistant Pseudomonas aeruginosa. Antimicrob Agents Chemother 64:e01587-19. doi:10.1128/AAC.01587-19 31611351 PMC7187587

[34] Han M-L, Zhu Y, Creek DJ, Lin Y-W, Gutu AD, Hertzog P, Purcell T, Shen H-H, Moskowitz SM, Velkov T, Li J. 2019. Comparative metabolomics and transcriptomics reveal multiple pathways associated with polymyxin killing in Pseudomonas aeruginosa. mSystems 4:e00149-18. doi:10.1128/mSystems.00149-18 30637340 PMC6325167

[35] Martinez OE. 2022. Structural and biochemical insights into bacterial cell wall glycopolymer display. University of California, Los Angeles.

[36] Cronan JE, Rock CO. 2008. Biosynthesis of membrane lipids. EcoSal Plus 3. doi:10.1128/ecosalplus.3.6.4 26443744

[37] Ogawa T, Tanaka A, Kawamoto J, Kurihara T. 2018. Purification and characterization of 1-acyl-sn-glycerol-3-phosphate acyltransferase with a substrate preference for polyunsaturated fatty acyl donors from the eicosapentaenoic acid-producing bacterium Shewanella livingstonensis Ac10. J Biochem 164:33–39. doi:10.1093/jb/mvy025 29415144

[38] Joondan N, Jhaumeer-Laulloo S, Caumul P. 2014. A study of the antibacterial activity of l-phenylalanine and l-tyrosine esters in relation to their CMCs and their interactions with 1, 2-dipalmitoyl-sn-glycero-3-phosphocholine, DPPC as model membrane. Microbiol Res 169:675–685. doi:10.1016/j.micres.2014.02.010 24667307

[39] Workman SD, Worrall LJ, Strynadka NCJ. 2018. Crystal structure of an intramembranal phosphatase central to bacterial cell-wall peptidoglycan biosynthesis and lipid recycling. Nat Commun 9:1159. doi:10.1038/s41467-018-03547-8 29559664 PMC5861054

[40] Brown S, Santa Maria JP, Walker S. 2013. Wall teichoic acids of Gram-positive bacteria. Annu Rev Microbiol 67:313–336. doi:10.1146/annurev-micro-092412-155620 24024634 PMC3883102

[41] Sewell EW, Brown ED. 2014. Taking aim at wall teichoic acid synthesis: new biology and new leads for antibiotics. J Antibiot 67:43–51. doi:10.1038/ja.2013.100 24169797

[42] Bouhss A, Trunkfield AE, Bugg TDH, Mengin-Lecreulx D. 2008. The biosynthesis of peptidoglycan lipid-linked intermediates. FEMS Microbiol Rev 32:208–233. doi:10.1111/j.1574-6976.2007.00089.x 18081839

[43] Baddiley J. 1970. Structure, biosynthesis, and function of teichoic acids. Acc Chem Res 3:98–105. doi:10.1021/ar50027a003

[44] Mierziak J, Burgberger M, Wojtasik W. 2021. 3-hydroxybutyrate as a metabolite and a signal molecule regulating processes of living organisms. Biomolecules 11:402. doi:10.3390/biom11030402 33803253 PMC8000602

[45] Chen Y, Chen T, Yin J. 2023. Impact of N-butyryl-l-homoserine lactone–mediated quorum sensing on acidogenic fermentation under saline conditions: insights into volatile fatty acids production and microbial community. Bioresour Technol 368:128354. doi:10.1016/j.biortech.2022.128354 36410593

[46] Pasquina-Lemonche L, Burns J, Turner RD, Kumar S, Tank R, Mullin N, Wilson JS, Chakrabarti B, Bullough PA, Foster SJ, Hobbs JK. 2020. The architecture of the Gram-positive bacterial cell wall. Nature 582:294–297. doi:10.1038/s41586-020-2236-6 32523118 PMC7308169

[47] van Dalen R, Peschel A, van Sorge NM. 2020. Wall teichoic acid in Staphylococcus aureus host interaction. Trends Microbiol 28:985–998. doi:10.1016/j.tim.2020.05.017 32540314

[48] Bärland N, Rueff A-S, Cebrero G, Hutter CAJ, Seeger MA, Veening J-W, Perez C. 2022. Mechanistic basis of choline import involved in teichoic acids and lipopolysaccharide modification. Sci Adv 8:eabm1122. doi:10.1126/sciadv.abm1122 35235350 PMC8890701

[49] Ralevic V. 2008. UDP-glucose, p 1–4. In Enna SJ, Bylund DB (ed), xPharm: the comprehensive pharmacology reference. Elsevier, New York.

[50] Henriksen ST, Liu J, Estiu G, Oltvai ZN, Wiest O. 2010. Identification of novel bacterial histidine biosynthesis inhibitors using docking, ensemble rescoring, and whole-cell assays. Bioorg Med Chem 18:5148–5156. doi:10.1016/j.bmc.2010.05.060 20573514 PMC2903657

[51] Rébora K, Laloo B, Daignan-Fornier B. 2005. Revisiting purine-histidine cross-pathway regulation in Saccharomyces cerevisiae: a central role for a small molecule. Genetics 170:61–70. doi:10.1534/genetics.104.039396 15744050 PMC1449729

[52] Winkler ME, Ramos-Montañez S. 2009. Biosynthesis of histidine. EcoSal Plus 3. doi:10.1128/ecosalplus.3.6.1.9 PMC4251894 26443768

[53] Mascher T, Helmann JD, Unden G. 2006. Stimulus perception in bacterial signal-transducing histidine kinases. Microbiol Mol Biol Rev 70:910–938. doi:10.1128/MMBR.00020-06 17158704 PMC1698512

[54] Moffatt BA, Ashihara H. 2002. Purine and pyrimidine nucleotide synthesis and metabolism. Arabidopsis Book 1:e0018. doi:10.1199/tab.0018 22303196 PMC3243375

[55] Yang JH, Wright SN, Hamblin M, McCloskey D, Alcantar MA, Schrübbers L, Lopatkin AJ, Satish S, Nili A, Palsson BO, Walker GC, Collins JJ. 2019. A white-box machine learning approach for revealing antibiotic mechanisms of action. Cell 177:1649–1661. doi:10.1016/j.cell.2019.04.016 31080069 PMC6545570

[56] Lopatkin AJ, Yang JH. 2021. Digital insights into nucleotide metabolism and antibiotic treatment failure. Front Digit Health 3:583468. doi:10.3389/fdgth.2021.583468 34355212 PMC8336923

[57] Jenal U, Reinders A, Lori C. 2017. Cyclic di-GMP: second messenger extraordinaire. Nat Rev Microbiol 15:271–284. doi:10.1038/nrmicro.2016.190 28163311

[58] Matange N. 2015. Revisiting bacterial cyclic nucleotide phosphodiesterases: cyclic AMP hydrolysis and beyond. FEMS Microbiol Lett 362:fnv183. doi:10.1093/femsle/fnv183 26424768

[59] Berens RL, Krug EC, Marr JJ. 1995. Purine and pyrimidine metabolism, p 89–117. In Biochemistry and molecular biology of parasites. Elsevier.

[60] Nagy PL, McCorkle GM, Zalkin H. 1993. purU, a source of formate for purT-dependent phosphoribosyl-N-formylglycinamide synthesis. J Bacteriol 175:7066–7073. doi:10.1128/jb.175.21.7066-7073.1993 8226647 PMC206834

[61] Mempin R, Tran H, Chen C, Gong H, Kim Ho K, Lu S. 2013. Release of extracellular ATP by bacteria during growth. BMC Microbiol 13:1–13. doi:10.1186/1471-2180-13-301 24364860 PMC3882102

[62] Löffler M, Carrey EA, Zameitat E. 2015. Orotic acid, more than just an intermediate of pyrimidine de novo synthesis. J Genet Genomics 42:207–219. doi:10.1016/j.jgg.2015.04.001 26059769

[63] Murima P, McKinney JD, Pethe K. 2014. Targeting bacterial central metabolism for drug development. Chem Biol 21:1423–1432. doi:10.1016/j.chembiol.2014.08.020 25442374

[64] Wu Z, Liang X, Li M, Ma M, Zheng Q, Li D, An T, Wang G. 2023. Advances in the optimization of central carbon metabolism in metabolic engineering. Microb Cell Fact 22:76. doi:10.1186/s12934-023-02090-6 37085866 PMC10122336

[65] Fang FC, Frawley ER, Tapscott T, Vázquez-Torres A. 2016. Bacterial stress responses during host infection. Cell Host Microbe 20:133–143. doi:10.1016/j.chom.2016.07.009 27512901 PMC4985009

[66] Tong M, Brown ED. 2023. Food for thought: opportunities to target carbon metabolism in antibacterial drug discovery. Ann N Y Acad Sci 1524:51–64. doi:10.1111/nyas.14991 37005709

[67] Keller M, Han X, Dörr T. 2022. Disrupting central carbon metabolism increases antibiotic susceptibility in Vibrio cholerae. bioRxiv. doi:10.1101/2022.12.22.521713 PMC10029711 36840595

[68] Pernas L. 2021. Cellular metabolism in the defense against microbes. J Cell Sci 134:jcs252023. doi:10.1242/jcs.252023 33558420

[69] Zhang YJ, Rubin EJ. 2013. Feast or famine: the host–pathogen battle over amino acids. Cell Microbiol 15:1079–1087. doi:10.1111/cmi.12140 23521858 PMC6434321

[70] Hennigar SR, McClung JP. 2016. Nutritional immunity: starving pathogens of trace minerals. Am J Lifestyle Med 10:170–173. doi:10.1177/1559827616629117 30202269 PMC6124953

[71] Xiong L, Teng JLL, Botelho MG, Lo RC, Lau SKP, Woo PCY. 2016. Arginine metabolism in bacterial pathogenesis and cancer therapy. Int J Mol Sci 17:363. doi:10.3390/ijms17030363 26978353 PMC4813224

[72] Leonardi R, Jackowski S. 2007. Biosynthesis of pantothenic acid and coenzyme A. EcoSal Plus 2. doi:10.1128/ecosalplus.3.6.3.4 PMC4950986 26443589

[73] Kolbe A, Tiessen A, Schluepmann H, Paul M, Ulrich S, Geigenberger P. 2005. Trehalose 6-phosphate regulates starch synthesis via posttranslational redox activation of ADP-glucose pyrophosphorylase. Proc Natl Acad Sci U S A 102:11118–11123. doi:10.1073/pnas.0503410102 16046541 PMC1180623

[74] Ruhal R, Kataria R, Choudhury B. 2013. Trends in bacterial trehalose metabolism and significant nodes of metabolic pathway in the direction of trehalose accumulation. Microb Biotechnol 6:493–502. doi:10.1111/1751-7915.12029 23302511 PMC3918152

[75] Iturriaga G, Suárez R, Nova-Franco B. 2009. Trehalose metabolism: from osmoprotection to signaling. Int J Mol Sci 10:3793–3810. doi:10.3390/ijms10093793 19865519 PMC2769160

[76] Wargo MJ. 2013. Homeostasis and catabolism of choline and glycine betaine: lessons from Pseudomonas aeruginosa. Appl Environ Microbiol 79:2112–2120. doi:10.1128/AEM.03565-12 23354714 PMC3623244

[77] Stincone A, Prigione A, Cramer T, Wamelink MMC, Campbell K, Cheung E, Olin-Sandoval V, Grüning N-M, Krüger A, Tauqeer Alam M, Keller MA, Breitenbach M, Brindle KM, Rabinowitz JD, Ralser M. 2015. The return of metabolism: biochemistry and physiology of the pentose phosphate pathway. Biol Rev Camb Philos Soc 90:927–963. doi:10.1111/brv.12140 25243985 PMC4470864

[78] Maifiah MHM, Creek DJ, Nation RL, Forrest A, Tsuji BT, Velkov T, Li J. 2017. Untargeted metabolomics analysis reveals key pathways responsible for the synergistic killing of colistin and doripenem combination against Acinetobacter baumannii. Sci Rep 7:45527. doi:10.1038/srep45527 28358014 PMC5371981

[79] Creek DJ, Jankevics A, Burgess KEV, Breitling R, Barrett MP. 2012. IDEOM: an excel interface for analysis of LC–MS-based metabolomics data. Bioinformatics 28:1048–1049. doi:10.1093/bioinformatics/bts069 22308147

[80] Scheltema RA, Jankevics A, Jansen RC, Swertz MA, Breitling R. 2011. PeakML/mzMatch: a file format, Java library, R library, and tool-chain for mass spectrometry data analysis. Anal Chem 83:2786–2793. doi:10.1021/ac2000994 21401061

[81] Pang Z, Chong J, Zhou G, de Lima Morais DA, Chang L, Barrette M, Gauthier C, Jacques P-É, Li S, Xia J. 2021. MetaboAnalyst 5.0: narrowing the gap between raw spectra and functional insights. Nucleic Acids Res 49:W388–W396. doi:10.1093/nar/gkab382 34019663 PMC8265181

[82] Kanehisa M, Sato Y. 2020. KEGG Mapper for Inferring cellular functions from protein sequences. Protein Sci 29:28–35. doi:10.1002/pro.3711 31423653 PMC6933857

