# Supplementary material for: Providing insight into the mechanism of action of cationic lipidated oligomers using metabolomics
Source: mSystems. 2024 Apr 12;9(5):e00093-24. doi: 10.1128/msystems.00093-24 (PMC11097639; doi:10.1128/msystems.00093-24)
Supplement: Table S1 — Metabolite IDs. [file msystems.00093-24-s0002.docx]

**Table S1.** Significant metabolites putatively identified following exposure to C_12_-o-(BG-D)-10 in methicillin-resistant *Staphylococcus aureus* (MRSA) ATCC 43300. Significant metabolites (highlighted in bold and italic) were determined using two-sample *t*-tests [ log_2_-fold change [FC] ≥ 0.59 or ≤ -0.59, corresponding to a metabolite level change of approximately 1.5-fold; False Discovery Rate (FDR) adjusted *p*-value ≤0.05].

| 1 hr Metabolites | | | | | | | | |
| --- | --- | --- | --- | --- | --- | --- | --- | --- |
| Mass  (g/mol) | **Retention Time**  **(min)** | **Formula** | **Putative metabolites** | **Confidence** | **Map** | **Pathway** | **Log2-fold change** | **FDR adjusted *p*-value** |
| 148.0372 | 15.7353 | C5H8O5 | (R)-2-Hydroxyglutarate | 8 | Amino Acid Metabolism | glutamate degradation V (via hydroxyglutarate) | ***-2.76950*** | 0.00000 |
| 113.0477 | 8.5001 | C5H7NO2 | (S)-1-Pyrroline-5-carboxylate | 8 | Amino Acid Metabolism | Glutamate metabolism, Arginine and proline metabolism | -1.15260 | 0.00028 |
| 111.0797 | 28.2827 | C5H9N3 | 1H-Imidazole-4-ethanamine | 5 | Amino Acid Metabolism | Histidine metabolism | ***0.81590*** | 0.00001 |
| 102.0317 | 7.2680 | C4H6O3 | 2-Oxobutanoate | 8 | Amino Acid Metabolism | Glycine, serine and threonine metabolism, Methionine metabolism, Valine, leucine and isoleucine biosynthesis, Propanoate metabolism, C5-Branched dibasic acid metabolism | -0.30477 | 0.01935 |
| 180.0899 | 9.4283 | C9H12N2O2 | 3-Hydroxykynurenamine | 5 | Amino Acid Metabolism | Tryptophan metabolism | -2.50310 | 0.00000 |
| 851.1713 | 9.5834 | C26H44N7O17P3S | 3-Methylbutanoyl-CoA | 8 | Amino Acid Metabolism | Valine, leucine and isoleucine degradation | -2.53530 | 0.00000 |
| 87.0684 | 4.8882 | C4H9NO | 4-Aminobutanal | 8 | Amino Acid Metabolism | Arginine and proline metabolism, beta-Alanine metabolism | 0.66117 | 0.00052 |
| 103.0634 | 6.0104 | C4H9NO2 | 4-Aminobutanoate | 8 | Amino Acid Metabolism | Arginine and proline metabolism, Glutamate metabolism, beta-Alanine metabolism, Butanoate metabolism | -4.40690 | 0.00000 |
| 136.0525 | 7.1063 | C8H8O2 | 4-Hydroxyphenylacetaldehyde | 6 | Amino Acid Metabolism | Tyrosine metabolism, Alkaloid biosynthesis I | 1.10750 | 0.02341 |
| 142.0379 | 13.3423 | C5H6N2O3 | 4-Imidazolone-5-acetate | 7 | Amino Acid Metabolism | Histidine metabolism | ***-2.65560*** | 0.00020 |
| 156.0535 | 11.4999 | C6H8N2O3 | 4-Imidazolone-5-propanoate | 8 | Amino Acid Metabolism | Histidine metabolism | ***-1.40230*** | 0.02299 |
| 129.0426 | 10.4948 | C5H7NO3 | 4-Oxoproline | 7 | Amino Acid Metabolism | Arginine and proline metabolism | -2.55140 | 0.00000 |
| 131.0583 | 13.7925 | C5H9NO3 | 5-Aminolevulinate | 8 | Amino Acid Metabolism | Glycine, serine and threonine metabolism, Porphyrin and chlorophyll metabolism | -1.50430 | 0.00003 |
| 297.0900 | 15.5806 | C11H15N5O3S | 5'-Methylthioadenosine | 6 | Amino Acid Metabolism | Arginine and proline metabolism, Methionine metabolism, Zeatin biosynthesis | -2.26590 | 0.00961 |
| 324.0242 | 16.2085 | C10H13O10P | 5-O-(1-Carboxyvinyl)-3-phosphoshikimate | 6 | Amino Acid Metabolism | Phenylalanine, tyrosine and tryptophan biosynthesis | -2.77170 | 0.00000 |
| 140.9829 | 18.9036 | CH4NO5P | Carbamoyl phosphate | 6 | Amino Acid Metabolism | Arginine and proline metabolism, Purine metabolism, Pyrimidine metabolism, Glutamate metabolism, Nitrogen metabolism | ***0.83076*** | 0.00246 |
| 103.0997 | 20.7356 | C5H13NO | Choline | 8 | Amino Acid Metabolism | Glycine, serine and threonine metabolism, Glycerophospholipid metabolism | ***-3.32600*** | 0.00000 |
| 160.0848 | 11.0998 | C6H12N2O3 | D-Alanyl-D-alanine | 8 | Amino Acid Metabolism | D-Alanine metabolism, Peptidoglycan biosynthesis | ***-2.04190*** | 0.00000 |
| 142.0742 | 12.5569 | C6H10N2O2 | Ectoine | 5 | Amino Acid Metabolism | Glycine, serine and threonine metabolism | -2.00530 | 0.00000 |
| 199.0959 | 11.5965 | C8H13N3O3 | gamma-Glutamyl-beta-aminopropiononitrile | 5 | Amino Acid Metabolism | Cyanoamino acid metabolism | -3.98950 | 0.00000 |
| 216.1108 | 8.5437 | C9H16N2O4 | gamma-Glutamyl-gamma-aminobutyraldehyde | 7 | Amino Acid Metabolism | Arginine and proline metabolism | -1.90850 | 0.00926 |
| 161.0688 | 9.6548 | C6H11NO4 | L-2-Aminoadipate | 8 | Amino Acid Metabolism | Lysine biosynthesis, Lysine degradation, Penicillin and cephalosporin biosynthesis | -0.34900 | 0.00624 |
| 89.0478 | 14.5914 | C3H7NO2 | L-Alanine | 8 | Amino Acid Metabolism | Alanine and aspartate metabolism, Cysteine metabolism, Taurine and hypotaurine metabolism, Selenoamino acid metabolism, D-Alanine metabolism, Carbon fixation, Reductive carboxylate cycle (CO2 fixation) | ***-1.96500*** | 0.00006 |
| 174.1117 | 23.2214 | C6H14N4O2 | L-Arginine | 8 | Amino Acid Metabolism | Arginine and proline metabolism, Clavulanic acid biosynthesis, D-Arginine and D-ornithine metabolism | -0.72173 | 0.00291 |
| 227.0795 | 14.6162 | C10H13NO5 | L-Arogenate | 6 | Amino Acid Metabolism | Phenylalanine, tyrosine and tryptophan biosynthesis | -3.50130 | 0.00052 |
| 132.0536 | 15.1918 | C4H8N2O3 | L-Asparagine | 6 | Amino Acid Metabolism | Alanine and aspartate metabolism, Tetracycline biosynthesis, Cyanoamino acid metabolism, Nitrogen metabolism | -2.22200 | 0.00001 |
| 133.0375 | 15.2080 | C4H7NO4 | L-Aspartate | 8 | Amino Acid Metabolism | Alanine and aspartate metabolism, Arginine and proline metabolism, Glycine, serine and threonine metabolism, Lysine biosynthesis, Arginine and proline metabolism, Histidine metabolism, beta-Alanine metabolism, Cyanoamino acid metabolism, Carbon fixation | ***-1.57140*** | 0.00007 |
| 161.1052 | 12.7187 | C7H15NO3 | L-Carnitine | 7 | Amino Acid Metabolism | Lysine degradation | -2.62820 | 0.00000 |
| 175.0957 | 15.6479 | C6H13N3O3 | L-Citrulline | 8 | Amino Acid Metabolism | Arginine and proline metabolism | ***-3.02830*** | 0.00000 |
| 121.0197 | 8.2078 | C3H7NO2S | L-Cysteine | 6 | Amino Acid Metabolism | Glycine, serine and threonine metabolism, Methionine metabolism, Cysteine metabolism, Penicillin and cephalosporin biosynthesis, Taurine and hypotaurine metabolism, Glutathione metabolism, Thiamine metabolism, Pantothenate and CoA biosynthesis | ***-2.48440*** | 0.00000 |
| 163.0481 | 12.6491 | C5H9NO5 | L-erythro-4-Hydroxyglutamate | 7 | Amino Acid Metabolism | Arginine and proline metabolism | -1.81070 | 0.00003 |
| 147.0531 | 14.8480 | C5H9NO4 | L-Glutamate | 6 | Amino Acid Metabolism | Arginine and proline metabolism, Glutamate metabolism, Histidine metabolism, D-Glutamine and D-glutamate metabolism, Glutathione metabolism, Butanoate metabolism, C5-Branched dibasic acid metabolism, Porphyrin and chlorophyll metabolism, Nitrogen metabolism | -0.94216 | 0.00213 |
| 146.0692 | 14.8369 | C5H10N2O3 | L-Glutamine | 8 | Amino Acid Metabolism | Glutamate metabolism, Purine metabolism, Pyrimidine metabolism, D-Glutamine and D-glutamate metabolism, Nitrogen metabolism | -3.22600 | 0.00065 |
| 155.0695 | 14.7300 | C6H9N3O2 | L-Histidine | 8 | Amino Acid Metabolism | Histidine metabolism, beta-Alanine metabolism | 0.70178 | 0.00200 |
| 190.0953 | 21.9441 | C7H14N2O4 | LL-2,6-Diaminoheptanedioate | 6 | Amino Acid Metabolism | Lysine biosynthesis | -2.03130 | 0.00693 |
| 146.1055 | 21.9485 | C6H14N2O2 | L-Lysine | 8 | Amino Acid Metabolism | Lysine biosynthesis, Lysine degradation, Biotin metabolism, Alkaloid biosynthesis II | -1.40080 | 0.00034 |
| 149.0511 | 11.1425 | C5H11NO2S | L-Methionine | 8 | Amino Acid Metabolism | Methionine metabolism | -0.65185 | 0.00030 |
| 132.0899 | 21.2008 | C5H12N2O2 | L-Ornithine | 8 | Amino Acid Metabolism | Arginine and proline metabolism, D-Arginine and D-ornithine metabolism, Glutathione metabolism | ***-1.82180*** | 0.00004 |
| 119.0582 | 14.7210 | C4H9NO3 | L-Threonine | 8 | Amino Acid Metabolism | Glycine, serine and threonine metabolism, Valine, leucine and isoleucine biosynthesis, Porphyrin and chlorophyll metabolism | -1.45450 | 0.00199 |
| 290.1229 | 16.6755 | C10H18N4O6 | N-(L-Arginino)succinate | 8 | Amino Acid Metabolism | Arginine and proline metabolism, Alanine and aspartate metabolism | ***-2.45090*** | 0.00000 |
| 274.1278 | 13.0316 | C10H18N4O5 | N2-Succinyl-L-arginine | 6 | Amino Acid Metabolism | Arginine and proline metabolism | -1.20400 | 0.00438 |
| 232.1058 | 8.8266 | C9H16N2O5 | N2-Succinyl-L-ornithine | 6 | Amino Acid Metabolism | Arginine and proline metabolism | -1.72980 | 0.00001 |
| 301.0563 | 14.6784 | C8H16NO9P | N-Acetyl-D-glucosamine 6-phosphate | 8 | Amino Acid Metabolism | Glutamate metabolism, Aminosugars metabolism | ***-2.25780*** | 0.02515 |
| 173.0688 | 7.1114 | C7H11NO4 | N-Acetyl-L-glutamate 5-semialdehyde | 8 | Amino Acid Metabolism | Arginine and proline metabolism | ***-1.81020*** | 0.01253 |
| 189.0637 | 14.4359 | C7H11NO5 | N-Acetyl-L-glutamate | 8 | Amino Acid Metabolism | Arginine and proline metabolism | ***-1.09830*** | 0.00006 |
| 174.1005 | 9.7524 | C7H14N2O3 | N-Acetylornithine | 8 | Amino Acid Metabolism | Arginine and proline metabolism | -1.35990 | 0.00096 |
| 190.0590 | 16.9523 | C6H10N2O5 | N-Carbamyl-L-glutamate | 7 | Amino Acid Metabolism | Histidine metabolism | ***-2.00340*** | 0.00085 |
| 174.0640 | 14.6705 | C6H10N2O4 | N-Formimino-L-glutamate | 8 | Amino Acid Metabolism | Histidine metabolism | -1.17450 | 0.00011 |
| 175.0480 | 15.0158 | C6H9NO5 | N-Formyl-L-glutamate | 8 | Amino Acid Metabolism | Histidine metabolism | ***-1.32630*** | 0.00004 |
| 177.0459 | 8.3939 | C6H11NO3S | N-Formyl-L-methionine | 7 | Amino Acid Metabolism | Methionine metabolism | -1.18040 | 0.00467 |
| 199.0247 | 16.0295 | C4H10NO6P | O-Phospho-L-homoserine | 8 | Amino Acid Metabolism | Glycine, serine and threonine metabolism | ***-3.96110*** | 0.00001 |
| 185.0089 | 16.6012 | C3H8NO6P | O-Phospho-L-serine | 8 | Amino Acid Metabolism | Glycine, serine and threonine metabolism, Cysteine metabolism | ***-1.68560*** | 0.00110 |
| 219.0740 | 14.1218 | C8H13NO6 | O-Succinyl-L-homoserine | 8 | Amino Acid Metabolism | Methionine metabolism, Sulfur metabolism | -2.41270 | 0.00000 |
| 174.0528 | 4.7017 | C7H10O5 | Shikimate | 6 | Amino Acid Metabolism | Phenylalanine, tyrosine and tryptophan biosynthesis | 0.89170 | 0.00360 |
| 679.1029 | 15.9349 | C20H31N3O19P2 | UDP-N-acetylmuramate | 8 | Amino Acid Metabolism | D-Glutamine and D-glutamate metabolism, Aminosugars metabolism, Peptidoglycan biosynthesis | ***-2.11710*** | 0.00021 |
| 750.1397 | 15.5757 | C23H36N4O20P2 | UDP-N-acetylmuramoyl-L-alanine | 8 | Amino Acid Metabolism | D-Glutamine and D-glutamate metabolism, Peptidoglycan biosynthesis | ***-1.12460*** | 0.00537 |
| 138.0430 | 28.7932 | C6H6N2O2 | Urocanate | 8 | Amino Acid Metabolism | Histidine metabolism | 0.74249 | 0.00014 |
| 197.1164 | 11.5505 | C9H15N3O2 | Hercynine | 5 | Amino Acid Metabolism | Histidine metabolism | ***-2.03050*** | 0.00001 |
| 172.0484 | 7.5729 | C6H8N2O4 | Hydantoin-5-propionate | 5 | Amino Acid Metabolism | Histidine metabolism | ***-2.67120*** | 0.00424 |
| 398.0997 | 17.4363 | C21H18O8 | Dihydro-NAME | 7 | Biosynthesis of Polyketides and Nonribosomal Peptides | Biosynthesis of type II polyketide products | -3.55360 | 0.00310 |
| 529.0866 | 14.8523 | C16H25N3O13P2 | dTDP-3-amino-2,3,6-trideoxy-D-threo-hexopyranos-4-ulose | 5 | Biosynthesis of Polyketides and Nonribosomal Peptides | Biosynthesis of vancomycin group antibiotics | -2.66360 | 0.00343 |
| 155.0582 | 8.8894 | C7H9NO3 | (3S,5R)-carbapenam | 7 | Biosynthesis of Secondary Metabolites | (5R)-carbapenem biosynthesis | -2.50490 | 0.00000 |
| 90.0317 | 10.0198 | C3H6O3 | (S)-Lactate | 8 | Carbohydrate Metabolism | Glycolysis / Gluconeogenesis, Pyruvate metabolism, Propanoate metabolism, Styrene degradation | -1.71320 | 0.00115 |
| 536.1597 | 3.2201 | C18H32O18 | 1-4-beta-D-Glucan | 5 | Carbohydrate Metabolism | Starch and sucrose metabolism | 2.50960 | 0.00017 |
| 195.0743 | 16.9602 | C6H13NO6 | 2-Amino-2-deoxy-D-gluconate | 5 | Carbohydrate Metabolism | Pentose phosphate pathway, Aminosugars metabolism | -2.57240 | 0.00000 |
| 214.0242 | 11.8548 | C5H11O7P | 2-Deoxy-D-ribose 1-phosphate | 8 | Carbohydrate Metabolism | Pentose phosphate pathway, Pyrimidine metabolism | -3.67890 | 0.00001 |
| 146.0216 | 16.3543 | C5H6O5 | 2-Oxoglutarate | 8 | Carbohydrate Metabolism | Citrate cycle (TCA cycle), Ascorbate and aldarate metabolism, Glutamate metabolism, Alanine and aspartate metabolism, Lysine biosynthesis, Histidine metabolism, D-Glutamine and D-glutamate metabolism, Glyoxylate and dicarboxylate metabolism | ***-1.21980*** | 0.00244 |
| 162.0528 | 13.1369 | C6H10O5 | 3-Ethylmalate | 7 | Carbohydrate Metabolism | Glyoxylate and dicarboxylate metabolism | 0.79570 | 0.00034 |
| 185.9929 | 17.4534 | C3H7O7P | 3-Phospho-D-glycerate | 8 | Carbohydrate Metabolism | Glycolysis / Gluconeogenesis, Glycine, serine and threonine metabolism, Glycerolipid metabolism, Glyoxylate and dicarboxylate metabolism, Carbon fixation | ***-2.68010*** | 0.00143 |
| 537.0767 | 16.8096 | C14H25N3O15P2 | CDP-ribitol | 7 | Carbohydrate Metabolism | Pentose and glucuronate interconversions | 0.67344 | 0.00098 |
| 192.0271 | 20.4849 | C6H8O7 | Citrate | 8 | Carbohydrate Metabolism | Citrate cycle (TCA cycle), Glutamate metabolism, Alanine and aspartate metabolism, Glyoxylate and dicarboxylate metabolism, Reductive carboxylate cycle (CO2 fixation) | ***-1.16990*** | 0.00319 |
| 767.1147 | 13.3423 | C21H36N7O16P3S | CoA | 8 | Carbohydrate Metabolism | Citrate cycle (TCA cycle), Fatty acid metabolism, Pantothenate and CoA biosynthesis | ***-2.38860*** | 0.00017 |
| 196.0583 | 16.0945 | C6H12O7 | D-Gluconic acid | 8 | Carbohydrate Metabolism | Pentose phosphate pathway | ***-1.67990*** | 0.00080 |
| 179.0794 | 16.2658 | C6H13NO5 | D-Glucosamine | 5 | Carbohydrate Metabolism | Aminosugars metabolism | ***-2.51720*** | 0.00000 |
| 260.0297 | 16.2538 | C6H13O9P | D-Glucose 6-phosphate | 6 | Carbohydrate Metabolism | Starch and sucrose metabolism, Streptomycin biosynthesis, Inositol phosphate metabolism | -1.81110 | 0.00008 |
| 180.0634 | 12.4954 | C6H12O6 | D-Glucose | 8 | Carbohydrate Metabolism | Glycolysis / Gluconeogenesis, Pentose phosphate pathway, Galactose metabolism, Starch and sucrose metabolism, Streptomycin biosynthesis, Indole and ipecac alkaloid biosynthesis | ***-2.26030*** | 0.00384 |
| 86.0370 | 3.5901 | C4H6O2 | Diacetyl | 8 | Carbohydrate Metabolism | Butanoate metabolism | -1.36940 | 0.03370 |
| 230.0190 | 15.9172 | C5H11O8P | D-Ribose 5-phosphate | 6 | Carbohydrate Metabolism | Pentose phosphate pathway, Purine metabolism, Carbon fixation | ***-2.59820*** | 0.00029 |
| 290.0404 | 16.3305 | C7H15O10P | D-Sedoheptulose 7-phosphate | 6 | Carbohydrate Metabolism | Pentose phosphate pathway, Carbon fixation | ***-2.23810*** | 0.00010 |
| 169.9980 | 16.3988 | C3H7O6P | Glycerone phosphate | 8 | Carbohydrate Metabolism | Glycolysis / Gluconeogenesis, Inositol metabolism, Pentose and glucuronate interconversions, Fructose and mannose metabolism, Galactose metabolism, Glycerolipid metabolism, Glycerophospholipid metabolism, Pyruvate metabolism, Carbon fixation | ***-3.73260*** | 0.00002 |
| 164.0685 | 10.8091 | C6H12O5 | L-Rhamnofuranose | 8 | Carbohydrate Metabolism | Fructose and mannose metabolism | -0.92585 | 0.00003 |
| 309.1062 | 13.4556 | C11H19NO9 | N-Acetylneuraminate | 6 | Carbohydrate Metabolism | Aminosugars metabolism | -1.79600 | 0.00005 |
| 319.0670 | 14.9725 | C8H18NO10P | N-Gluconyl ethanolamine phosphate | 5 | Carbohydrate Metabolism | Undefined | -2.79180 | 0.00000 |
| 167.9824 | 16.8761 | C3H5O6P | Phosphoenolpyruvate | 6 | Carbohydrate Metabolism | Glycolysis / Gluconeogenesis, Citrate cycle (TCA cycle), Phenylalanine, tyrosine and tryptophan biosynthesis, Aminophosphonate metabolism, Pyruvate metabolism, Carbon fixation, Reductive carboxylate cycle (CO2 fixation) | ***-2.63020*** | 0.03287 |
| 154.0031 | 12.0185 | C3H7O5P | Propanoyl phosphate | 8 | Carbohydrate Metabolism | Propanoate metabolism, C5-Branched dibasic acid metabolism | 4.12250 | 0.00000 |
| 88.0160 | 8.7945 | C3H4O3 | Pyruvate | 8 | Carbohydrate Metabolism | Glycolysis / Gluconeogenesis, Citrate cycle (TCA cycle), Pentose phosphate pathway, Ascorbate and aldarate metabolism, Biosynthesis of steroids__Alanine and aspartate metabolism__Glycine, serine and threonine metabolism | ***0.85066*** | 0.00269 |
| 118.0266 | 15.6987 | C4H6O4 | Succinate | 8 | Carbohydrate Metabolism | Citrate cycle (TCA cycle), Oxidative phosphorylation, Glutamate metabolism, Alanine and aspartate metabolism, Tyrosine metabolism, Phenylalanine metabolism, gamma-Hexachlorocyclohexane degradation, Glyoxylate and dicarboxylate metabolism | ***-1.07520*** | 0.00001 |
| 867.1307 | 15.5102 | C25H40N7O19P3S | Succinyl-CoA | 8 | Carbohydrate Metabolism | Citrate cycle (TCA cycle), Valine, leucine and isoleucine degradation, Benzoate degradation via hydroxylation, 1- and 2-Methylnaphthalene degradation, Benzoate degradation via CoA ligation, Propanoate metabolism, Reductive carboxylate cycle (CO2 fixation) | ***-2.71560*** | 0.00000 |
| 566.0552 | 16.6734 | C15H24N2O17P2 | UDP-glucose | 6 | Carbohydrate Metabolism | Pentose and glucuronate interconversions, Galactose metabolism, Ascorbate and aldarate metabolism, Pyrimidine metabolism, Starch and sucrose metabolism, Nucleotide sugars metabolism, Glycerolipid metabolism, Zeatin biosynthesis, Biosynthesis of ansamycins | ***-2.15730*** | 0.00022 |
| 607.0819 | 15.2307 | C17H27N3O17P2 | UDP-N-acetyl-D-glucosamine | 6 | Carbohydrate Metabolism | Aminosugars metabolism, Lipopolysaccharide biosynthesis, Peptidoglycan biosynthesis | ***-2.46460*** | 0.00015 |
| 427.0297 | 16.3139 | C10H15N5O10P2 | ADP | 8 | Energy Metabolism | Oxidative phosphorylation, Photosynthesis, Purine metabolism, Zeatin biosynthesis | ***-3.82290*** | 0.00001 |
| 506.9962 | 16.8687 | C10H16N5O13P3 | ATP | 8 | Energy Metabolism | Oxidative phosphorylation, Photosynthesis, Purine metabolism, Puromycin biosynthesis, Zeatin biosynthesis | -0.82983 | 0.00035 |
| 222.0675 | 16.8675 | C7H14N2O4S | Cystathionine | 8 | Energy Metabolism | Sulfur metabolism | -2.66120 | 0.00059 |
| 663.1090 | 13.5704 | C21H27N7O14P2 | NAD+ | 6 | Energy Metabolism | Oxidative phosphorylation, Glutamate metabolism, Nicotinate and nicotinamide metabolism | ***-2.51890*** | 0.00005 |
| 665.1233 | 13.3024 | C21H29N7O14P2 | NADH | 8 | Energy Metabolism | Oxidative phosphorylation | ***-4.16510*** | 0.00313 |
| 743.0754 | 16.6502 | C21H28N7O17P3 | NADP+ | 8 | Energy Metabolism | Photosynthesis, Glutathione metabolism, Nicotinate and nicotinamide metabolism | ***-2.54030*** | 0.00053 |
| 745.0911 | 17.3776 | C21H30N7O17P3 | NADPH | 8 | Energy Metabolism | Photosynthesis, Glutathione metabolism | ***-2.45370*** | 0.02754 |
| 97.9769 | 16.3546 | H3O4P | Orthophosphate | 7 | Energy Metabolism | Oxidative phosphorylation, Photosynthesis, Peptidoglycan biosynthesis | -3.37740 | 0.00313 |
| 177.9433 | 27.2114 | H4O7P2 | Pyrophosphate | 5 | Energy Metabolism | Oxidative phosphorylation | 0.69665 | 0.00621 |
| 574.6761 | 15.4063 | C40H65N9O26P2 | UDPMurAc(oyl-L-Ala-D-gamma-Glu-L-Lys-D-Ala-D-Ala) | 8 | Glycan Biosynthesis and Metabolism | Peptidoglycan biosynthesis | ***-1.25500*** | 0.00433 |
| 477.0557 | 16.2632 | C12H21N3O13P2 | CDP-glycerol | 5 | Lipid Metabolism | Glycerophospholipid metabolism | -3.01780 | 0.00001 |
| 104.0474 | 8.4394 | C4H8O3 | (R)-3-Hydroxybutanoate | 8 | Lipid Metabolism | Synthesis and degradation of ketone bodies, Butanoate metabolism | -2.67630 | 0.00001 |
| 257.1028 | 13.7581 | C8H20NO6P | sn-glycero-3-Phosphocholine | 8 | Lipid Metabolism | Glycerophospholipid metabolism, Ether lipid metabolism | -4.73520 | 0.00000 |
| 183.0662 | 14.6590 | C5H14NO4P | Choline phosphate | 8 | Lipid Metabolism | Glycerophospholipid metabolism, Glycine, serine and threonine metabolism | ***-3.07110*** | 0.00000 |
| 172.0137 | 15.0612 | C3H9O6P | sn-Glycerol 3-phosphate | 8 | Lipid Metabolism | Glycerolipid metabolism, Glycerophospholipid metabolism | -6.69940 | 0.00001 |
| 281.2718 | 3.6864 | C18H35NO | [FA (18:1)] 9Z-octadecenamide | 5 | Lipids: Fatty Acyls | Fatty amides | 0.92439 | 0.00032 |
| 282.2560 | 3.3645 | C18H34O2 | [FA (18:1)] 9Z-octadecenoic acid | 8 | Lipids: Fatty Acyls | Fatty acid biosynthesis, Biosynthesis of unsaturated fatty acids | 1.56350 | 0.00083 |
| 228.1000 | 10.8157 | C11H16O5 | [FA hydroxy(11:2/11:2)] 2R,9R-dihydroxy-3S,4S,7S,8S-diepoxy-5E,10-undecadien-1-ol | 7 | Lipids: Fatty Acyls | Fatty alcohols | 0.68002 | 0.00037 |
| 312.3030 | 3.2272 | C20H40O2 | FA (20:0) | 5 | Lipids: Fatty Acyls | Fatty Acids and Conjugates | ***4.02220*** | 0.00001 |
| 310.2874 | 3.3011 | C20H38O2 | FA (20:1) | 5 | Lipids: Fatty Acyls | Fatty Acids and Conjugates | ***3.64940*** | 0.00000 |
| 214.1572 | 27.3856 | C12H22O3 | 3-Oxododecanoic acid | 8 | Lipids: Fatty Acyls | Fatty Acids and Conjugates | 1.02180 | 0.00071 |
| 270.2559 | 3.3450 | C17H34O2 | FA (17:0) | 5 | Lipids: Fatty Acyls | Fatty Acids and Conjugates | ***3.50840*** | 0.00001 |
| 144.1150 | 27.5195 | C8H16O2 | FA (8:0) octanoic acid | 8 | Lipids: Fatty Acyls | Fatty acid biosynthesis | 2.04880 | 0.00199 |
| 126.1044 | 3.6087 | C8H14O | FA (8:1) octenal | 7 | Lipids: Fatty Acyls | Fatty aldehydes | 1.32620 | 0.01475 |
| 334.2875 | 3.2823 | C22H38O2 | FA dimethyl(20:3) | 5 | Lipids: Fatty Acyls | Fatty Acids and Conjugates | 2.88280 | 0.00001 |
| 258.2194 | 27.4429 | C15H30O3 | FA hydroxy(15:0) | 5 | Lipids: Fatty Acyls | Fatty Acids and Conjugates | 0.64767 | 0.01282 |
| 272.2352 | 3.5253 | C16H32O3 | FA hydroxy(16:0)] hexadecanoic acid | 5 | Lipids: Fatty Acyls | Fatty Acids and Conjugates | 1.34860 | 0.00112 |
| 300.2666 | 3.4965 | C18H36O3 | FA hydroxy(18:0) | 5 | Lipids: Fatty Acyls | Fatty Acids and Conjugates | 1.74920 | 0.00011 |
| 314.2457 | 3.3635 | C18H34O4 | FA hydroxy(18:1)] | 5 | Lipids: Fatty Acyls | Fatty Acids and Conjugates | 1.14830 | 0.00552 |
| 312.2303 | 3.6713 | C18H32O4 | FA hydroxy(18:2) | 5 | Lipids: Fatty Acyls | Octadecanoids | ***1.32190*** | 0.00366 |
| 326.2826 | 3.3869 | C20H38O3 | FA hydroxy(20:1) | 5 | Lipids: Fatty Acyls | Fatty Acids and Conjugates | ***2.33370*** | 0.00014 |
| 324.2666 | 3.3937 | C20H36O3 | FA hydroxy(20:2) | 5 | Lipids: Fatty Acyls | Eicosanoids | ***2.37160*** | 0.00011 |
| 322.2509 | 3.3355 | C20H34O3 | FA hydroxy(20:3) | 5 | Lipids: Fatty Acyls | Eicosanoids | 0.82071 | 0.00714 |
| 187.0845 | 4.6807 | C8H13NO4 | FA hydroxy(4:0) N-(3S-hydroxy-butanoyl)-homoserine lactone | 8 | Lipids: Fatty Acyls | Fatty amides | -1.29090 | 0.00184 |
| 132.0786 | 4.8176 | C6H12O3 | FA hydroxy(6:0) | 7 | Lipids: Fatty Acyls | Fatty Acids and Conjugates | -1.86480 | 0.00370 |
| 160.1100 | 7.1169 | C8H16O3 | FA hydroxy(8:0) hydroxy-octanoic acid | 7 | Lipids: Fatty Acyls | Fatty Acids and Conjugates | 0.66914 | 0.01471 |
| 214.1933 | 3.5409 | C13H26O2 | FA methyl(12:0) dodecanoic acid | 5 | Lipids: Fatty Acyls | Fatty Acids and Conjugates | 0.79030 | 0.00008 |
| 242.2246 | 3.4334 | C15H30O2 | FA methyl(14:0) | 5 | Lipids: Fatty Acyls | Fatty Acids and Conjugates | ***2.92200*** | 0.00002 |
| 244.1672 | 4.7216 | C13H24O4 | FA methyl(14:0) dodecanedioic acid | 5 | Lipids: Fatty Acyls | Fatty Acids and Conjugates | 0.65767 | 0.02986 |
| 268.2403 | 3.4094 | C17H32O2 | FA methyl(16:1) | 5 | Lipids: Fatty Acyls | Fatty Acids and Conjugates | 1.31720 | 0.00478 |
| 298.2872 | 3.2888 | C19H38O2 | FA methyl(18:0) | 5 | Lipids: Fatty Acyls | Fatty Acids and Conjugates | ***4.62640*** | 0.00000 |
| 308.2352 | 3.3613 | C19H32O3 | FA methyl(18:2) | 7 | Lipids: Fatty Acyls | Fatty Acids and Conjugates | 0.60250 | 0.01196 |
| 130.0994 | 4.6023 | C7H14O2 | FA methyl(6:0) | 5 | Lipids: Fatty Acyls | Fatty Acids and Conjugates | 1.15010 | 0.00347 |
| 158.1306 | 3.8574 | C9H18O2 | FA methyl(8:0) | 5 | Lipids: Fatty Acyls | Fatty Acids and Conjugates | 1.80520 | 0.00334 |
| 212.1413 | 3.6856 | C12H20O3 | FA oxo(12:1) | 5 | Lipids: Fatty Acyls | Fatty Acids and Conjugates | 2.15910 | 0.00039 |
| 238.1566 | 3.7285 | C14H22O3 | FA oxo(14:1) cyclopentanebutanoic acid | 5 | Lipids: Fatty Acyls | Octadecanoids | 1.12940 | 0.00221 |
| 256.2038 | 27.1490 | C15H28O3 | FA oxo(15:0) | 5 | Lipids: Fatty Acyls | Fatty Acids and Conjugates | 0.84002 | 0.00018 |
| 270.2195 | 3.4647 | C16H30O3 | FA oxo(16:0) | 5 | Lipids: Fatty Acyls | Fatty Acids and Conjugates | ***1.48680*** | 0.00470 |
| 298.2502 | 3.3297 | C18H34O3 | FA oxo(18:0) | 5 | Lipids: Fatty Acyls | Fatty Acids and Conjugates | ***2.04900*** | 0.00002 |
| 312.2666 | 3.4622 | C19H36O3 | FA oxo(19:0) | 5 | Lipids: Fatty Acyls | Fatty Acids and Conjugates | ***2.23400*** | 0.00003 |
| 340.2981 | 3.4084 | C21H40O3 | FA oxo(21:0) | 5 | Lipids: Fatty Acyls | Fatty Acids and Conjugates | ***1.54200*** | 0.00020 |
| 256.2403 | 3.3826 | C16H32O2 | FA(16:0) | 6 | Lipids: Fatty Acyls | Fatty acid biosynthesis, Fatty acid elongation in mitochondria, Fatty acid metabolism, Biosynthesis of unsaturated fatty acids | ***2.06230*** | 0.00085 |
| 351.3140 | 3.3689 | C22H41NO2 | N-(11Z,14Z-eicosadienoyl)-ethanolamine | 5 | Lipids: Fatty Acyls | Fatty amides | 4.71620 | 0.00014 |
| 353.3293 | 3.3768 | C22H43NO2 | N-(11Z-eicosaenoyl)-ethanolamine | 5 | Lipids: Fatty Acyls | Fatty amides | 3.85990 | 0.00173 |
| 171.0896 | 7.2128 | C8H13NO3 | N-Butyryl-L-homoserine lactone | 6 | Lipids: Fatty Acyls | Fatty amides | -0.90193 | 0.00179 |
| 285.2667 | 6.9842 | C17H35NO2 | Pentadecanoyl-EA | 7 | Lipids: Fatty Acyls | Fatty amides | 0.76012 | 0.00195 |
| 228.2089 | 6.9506 | C14H28O2 | Tetradecanoic acid | 5 | Lipids: Fatty Acyls | Fatty acid biosynthesis | 1.01200 | 0.01449 |
| 324.3030 | 3.2225 | C21H40O2 | [FA (21:2)] octadecenoic Acid | 5 | Lipids: Fatty Acyls | Fatty Acids and Conjugates | ***2.01130*** | 0.00197 |
| 102.0681 | 5.9090 | C5H10O2 | Pentanoate | 7 | Lipids: Fatty Acyls | Fatty Acids and Conjugates | 1.71260 | 0.01214 |
| 118.0630 | 7.0955 | C5H10O3 | 5-Hydroxypentanoate | 5 | Lipids: Fatty Acyls | Fatty Acids and Conjugates | -1.98890 | 0.00001 |
| 368.3298 | 3.5597 | C23H44O3 | FA oxo(23:0) | 5 | Lipids: Fatty Acyls | Fatty Acids and Conjugates | ***2.05190*** | 0.00299 |
| 296.2714 | 3.3116 | C19H36O2 | FA (19:1) | 5 | Lipids: Fatty Acyls | Fatty Acids and Conjugates | ***2.45310*** | 0.00006 |
| 338.3187 | 3.2221 | C22H42O2 | FA (22:1) | 5 | Lipids: Fatty Acyls | Fatty Acids and Conjugates | ***2.95870*** | 0.00003 |
| 596.5018 | 3.1050 | C36H68O6 | Glycerol triundecanoate | 5 | Lipids: Glycerolipids | Undefined | 0.75986 | 0.00005 |
| 358.3086 | 3.4156 | C21H42O4 | MG(18:0/0:0/0:0) | 5 | Lipids: Glycerolipids | Monoradylglycerols | 2.16820 | 0.00018 |
| 484.2808 | 3.3958 | C22H45O9P | [PG (16:0)] 1-hexadecanoyl-sn-glycero-3-phospho-(1'-sn-glycerol) | 5 | Lipids: Glycerophospholipids | Glycerophosphoglycerols | 0.59270 | 0.01391 |
| 248.1010 | 15.2310 | C9H16N2O6 | [PG (18:0)] 1-octadecanoyl-sn-glycero-3-phospho-(1'-sn-glycerol) | 7 | Lipids: Glycerophospholipids | Glycerophosphoglycerols | -0.69751 | 0.03387 |
| 610.3846 | 3.1824 | C30H59O10P | [PG (24:0)] | 5 | Lipids: Glycerophospholipids | Glycerophosphoglycerols | 0.78895 | 0.02778 |
| 572.2968 | 3.8794 | C25H49O12P | [PI(16:0)] 1-hexadecanoyl-sn-glycero-3-phospho-(1'-myo-inositol) | 5 | Lipids: Glycerophospholipids | Glycerophosphoinositols | 2.10920 | 0.00017 |
| 523.2915 | 3.7555 | C24H46NO9P | [PS(18:1)] 1-(9Z-octadecenoyl)-sn-glycero-3-phosphoserine | 5 | Lipids: Glycerophospholipids | Glycerophosphoserines | 1.86520 | 0.00138 |
| 804.5319 | 3.2668 | C46H77O9P | 1-(8-[3]-ladderane-octanoyl)-2-(8-[3]-ladderane-octanyl)-sn-glycero-3-phospho-(1'-sn-glycerol) | 5 | Lipids: Glycerophospholipids | Glycerophosphoglycerols | 1.99480 | 0.00000 |
| 680.4457 | 3.4145 | C35H69O8PS | 1,2-ditetradecanoyl-sn-glycero-3-phosphosulfocholine | 5 | Lipids: Glycerophospholipids | Undefined | ***3.70540*** | 0.00051 |
| 410.2435 | 4.0708 | C19H39O7P | LysoPA(16:0) | 5 | Lipids: Glycerophospholipids | Glycerophosphates | ***2.06060*** | 0.00028 |
| 425.2549 | 4.3098 | C19H40NO7P | LysoPE(0:0/14:0) | 5 | Lipids: Glycerophospholipids | Glycerophosphoethanolamines | 1.53130 | 0.00059 |
| 453.2865 | 4.2010 | C21H44NO7P | LysoPE(16:0) | 5 | Lipids: Glycerophospholipids | Glycerophosphoethanolamines | ***2.16310*** | 0.00023 |
| 451.2704 | 4.1969 | C21H42NO7P | LysoPE(16:1) | 5 | Lipids: Glycerophospholipids | Glycerophosphoethanolamines | ***2.08650*** | 0.00069 |
| 481.3176 | 4.2073 | C23H48NO7P | LysoPE(18:0) | 5 | Lipids: Glycerophospholipids | Glycerophosphoethanolamines | 1.37290 | 0.00019 |
| 479.3016 | 4.1073 | C23H46NO7P | LysoPE(18:1) | 5 | Lipids: Glycerophospholipids | Glycerophosphoethanolamines | 1.99100 | 0.00020 |
| 477.2861 | 4.0815 | C23H44NO7P | LysoPE(18:2) | 5 | Lipids: Glycerophospholipids | Glycerophosphoethanolamines | 1.88010 | 0.00073 |
| 775.6077 | 18.8495 | C43H86NO8P | PE(38:0) | 5 | Lipids: Glycerophospholipids | Glycerophosphoethanolamines | 0.83074 | 0.00477 |
| 470.2648 | 3.4492 | C21H43O9P | PG(15:0/0:0) | 5 | Lipids: Glycerophospholipids | Glycerophosphoglycerols | 0.71243 | 0.00019 |
| 762.4466 | 28.2974 | C42H67O10P | PG(18:4(6Z,9Z,12Z,15Z)/18:4(6Z,9Z,12Z,15Z)) | 7 | Lipids: Glycerophospholipids | Glycerophosphoglycerols | 1.17950 | 0.00919 |
| 624.4003 | 3.1600 | C31H61O10P | PG(25:0) | 7 | Lipids: Glycerophospholipids | Glycerophosphoglycerols | ***1.17500*** | 0.00537 |
| 652.4324 | 3.1391 | C33H65O10P | PG(27:0) | 5 | Lipids: Glycerophospholipids | Glycerophosphoglycerols | ***0.99652*** | 0.00796 |
| 694.4788 | 3.1206 | C36H71O10P | PG(30:0) | 5 | Lipids: Glycerophospholipids | Glycerophosphoglycerols | ***0.58726*** | 0.01361 |
| 401.2381 | 7.1205 | C38H76O13P2 | PGP(16:0/16:0) | 7 | Lipids: Glycerophospholipids | Undefined | ***1.03840*** | 0.03308 |
| 525.3069 | 3.7590 | C24H48NO9P | PS(18:0) | 5 | Lipids: Glycerophospholipids | Glycerophosphoserines | ***1.78680*** | 0.00490 |
| 747.5396 | 3.3203 | C40H78NO9P | PS(O-34:1) | 5 | Lipids: Glycerophospholipids | Glycerophosphoserines | ***1.49780*** | 0.00105 |
| 703.5169 | 3.2989 | C38H74NO8P | PC(30:1) | 5 | Lipids: Glycerophospholipids | Glycerophosphocholines | ***1.09360*** | 0.00303 |
| 519.3311 | 4.1873 | C26H50NO7P | PC(18:2) | 5 | Lipids: Glycerophospholipids | Glycerophosphocholines | ***1.41200*** | 0.00577 |
| 680.4770 | 3.2144 | C39H69O7P | PA(O-36:5) | 5 | Lipids: Glycerophospholipids | Glycerophosphates | ***1.79100*** | 0.00062 |
| 690.5188 | 7.1366 | C38H75O8P | PA(35:0) | 5 | Lipids: Glycerophospholipids | Glycerophosphates | ***-3.06360*** | 0.00003 |
| 424.2595 | 3.8603 | C20H41O7P | PA(17:0/0:0) | 5 | Lipids: Glycerophospholipids | Glycerophosphates | ***1.83040*** | 0.00034 |
| 848.4612 | 4.7338 | C42H74O13P2 | PGP(36:5) | 7 | Lipids: Glycerophospholipids | Undefined | ***2.98450*** | 0.00008 |
| 493.3176 | 4.2375 | C24H48NO7P | LysoPC(16:1) | 5 | Lipids: Glycerophospholipids | Glycerophosphocholines | 1.29780 | 0.00029 |
| 495.3330 | 4.2267 | C24H50NO7P | LysoPC(16:0) | 7 | Lipids: Glycerophospholipids | Glycerophosphocholines | 1.32160 | 0.00088 |
| 521.3489 | 4.0759 | C26H52NO7P | LysoPC(18:1) | 5 | Lipids: Glycerophospholipids | Glycerophosphocholines | ***5.78150*** | 0.00005 |
| 523.3638 | 4.0002 | C26H54NO7P | LysoPC(18:0) | 5 | Lipids: Glycerophospholipids | Glycerophosphocholines | ***1.14750*** | 0.01139 |
| 777.5534 | 3.2746 | C41H80NO10P | PS(35:0) | 5 | Lipids: Glycerophospholipids | Glycerophosphoserines | ***1.43260*** | 0.00011 |
| 526.3273 | 3.2875 | C25H51O9P | PG(19:0) | 7 | Lipids: Glycerophospholipids | Glycerophosphoglycerols | ***-2.01630*** | 0.00575 |
| 760.5055 | 3.2729 | C44H73O8P | PG (14:0) | 5 | Lipids: Glycerophospholipids | Glycerophosphoglycerols | ***2.02650*** | 0.00000 |
| 301.2982 | 4.3884 | C18H39NO2 | Sphinganine | 7 | Lipids: Sphingolipids | Sphingolipid metabolism | 1.72670 | 0.00617 |
| 592.3989 | 3.2740 | C34H56O8 | Collettiside I | 5 | Lipids: Sterol lipids | Sterols | 1.59720 | 0.00005 |
| 240.0637 | 7.1045 | C11H12O6 | (1R,6R)-6-Hydroxy-2-succinylcyclohexa-2,4-diene-1-carboxylate | 5 | Metabolism of Cofactors and Vitamins | Ubiquinone biosynthesis | -1.39180 | 0.01469 |
| 195.0757 | 9.2198 | C7H9N5O2 | 2-Amino-4-hydroxy-6-hydroxymethyl-7,8-dihydropteridine | 8 | Metabolism of Cofactors and Vitamins | Folate biosynthesis | 2.68500 | 0.00764 |
| 326.1228 | 13.1175 | C13H18N4O6 | 6,7-Dimethyl-8-(1-D-ribityl)lumazine | 8 | Metabolism of Cofactors and Vitamins | Riboflavin metabolism | -0.60394 | 0.00003 |
| 139.0269 | 13.9205 | C6H5NO3 | 6-Hydroxynicotinate | 7 | Metabolism of Cofactors and Vitamins | Nicotinate and nicotinamide metabolism | -1.60010 | 0.00001 |
| 188.1525 | 20.9271 | C9H20N2O2 | 7,8-Diaminononanoate | 8 | Metabolism of Cofactors and Vitamins | Biotin metabolism | -1.53950 | 0.00001 |
| 246.1037 | 8.6823 | C10H18N2O3S | 9-mercaptodethiobiotin | 7 | Metabolism of Cofactors and Vitamins | biotin biosynthesis II | 0.73824 | 0.02017 |
| 278.1267 | 10.3730 | C14H18N2O4 | alpha-Ribazole | 8 | Metabolism of Cofactors and Vitamins | Riboflavin metabolism__Porphyrin and chlorophyll metabolism | 1.07850 | 0.00784 |
| 226.0478 | 15.8936 | C10H10O6 | Chorismate | 6 | Metabolism of Cofactors and Vitamins | Ubiquinone biosynthesis, Phenylalanine, tyrosine and tryptophan biosynthesis, Folate biosynthesis, Biosynthesis of siderophore group nonribosomal peptides | -1.68220 | 0.00003 |
| 214.1317 | 8.9268 | C10H18N2O3 | Dethiobiotin | 8 | Metabolism of Cofactors and Vitamins | Biotin metabolism | 0.77419 | 0.01054 |
| 785.1564 | 11.0797 | C27H33N9O15P2 | FAD | 6 | Metabolism of Cofactors and Vitamins | Riboflavin metabolism | -0.81853 | 0.00070 |
| 131.0219 | 9.2559 | C4H5NO4 | Iminoaspartate | 8 | Metabolism of Cofactors and Vitamins | Nicotinate and nicotinamide metabolism | 0.80927 | 0.00989 |
| 115.0269 | 7.2352 | C4H5NO3 | Maleamate | 7 | Metabolism of Cofactors and Vitamins | Nicotinate and nicotinamide metabolism | 0.80516 | 0.00009 |
| 122.0480 | 7.1410 | C6H6N2O | Nicotinamide | 6 | Metabolism of Cofactors and Vitamins | Nicotinate and nicotinamide metabolism | -1.47370 | 0.00005 |
| 123.0321 | 7.8712 | C6H5NO2 | Nicotinate | 6 | Metabolism of Cofactors and Vitamins | Nicotinate and nicotinamide metabolism, Alkaloid biosynthesis II | 1.19840 | 0.00043 |
| 254.0898 | 24.3403 | C11H14N2O5 | N-Ribosylnicotinamide | 8 | Metabolism of Cofactors and Vitamins | Nicotinate and nicotinamide metabolism | -3.03040 | 0.00002 |
| 168.0898 | 5.6195 | C8H12N2O2 | Pyridoxamine | 7 | Metabolism of Cofactors and Vitamins | Vitamin B6 metabolism | 1.76200 | 0.00176 |
| 376.1382 | 7.8162 | C17H20N4O6 | Riboflavin | 6 | Metabolism of Cofactors and Vitamins | Riboflavin metabolism | 1.06150 | 0.00126 |
| 264.1045 | 21.3349 | C12H16N4OS | Thiamin | 8 | Metabolism of Cofactors and Vitamins | Thiamine metabolism | -2.89230 | 0.00007 |
| 329.0528 | 11.3317 | C10H12N5O6P | 3',5'-Cyclic AMP | 6 | Nucleotide Metabolism | Purine metabolism | ***-2.76330*** | 0.00547 |
| 128.0585 | 13.7965 | C5H8N2O2 | 5,6-Dihydrothymine | 8 | Nucleotide Metabolism | Pyrimidine metabolism | ***-1.60340*** | 0.00000 |
| 114.0430 | 7.1092 | C4H6N2O2 | 5,6-Dihydrouracil | 8 | Nucleotide Metabolism | Pyrimidine metabolism, beta-Alanine metabolism, Pantothenate and CoA biosynthesis | ***-1.84010*** | 0.00001 |
| 135.0545 | 8.5389 | C5H5N5 | Adenine | 8 | Nucleotide Metabolism | Purine metabolism, Zeatin biosynthesis | ***-1.23980*** | 0.00104 |
| 267.0966 | 8.4193 | C10H13N5O4 | Adenosine | 8 | Nucleotide Metabolism | Purine metabolism | -0.79972 | 0.00484 |
| 347.0632 | 13.5328 | C10H14N5O7P | AMP | 8 | Nucleotide Metabolism | Purine metabolism, Zeatin biosynthesis | ***-2.58320*** | 0.00004 |
| 403.0182 | 17.2381 | C9H15N3O11P2 | CDP | 8 | Nucleotide Metabolism | Pyrimidine metabolism | ***-1.23980*** | 0.00000 |
| 323.0520 | 15.8692 | C9H14N3O8P | CMP | 6 | Nucleotide Metabolism | Pyrimidine metabolism | ***-2.02730*** | 0.00013 |
| 111.0432 | 9.9875 | C4H5N3O | Cytosine | 8 | Nucleotide Metabolism | Pyrimidine metabolism | -0.81534 | 0.00165 |
| 411.0349 | 14.1563 | C10H15N5O9P2 | dADP | 8 | Nucleotide Metabolism | Purine metabolism | ***-2.71690*** | 0.00000 |
| 331.0681 | 12.4574 | C10H14N5O6P | dAMP | 5 | Nucleotide Metabolism | Purine metabolism | ***-1.66340*** | 0.00042 |
| 491.0013 | 16.2314 | C10H16N5O12P3 | dATP | 6 | Nucleotide Metabolism | Purine metabolism | ***-2.52620*** | 0.00000 |
| 387.0236 | 16.2051 | C9H15N3O10P2 | dCDP | 6 | Nucleotide Metabolism | Pyrimidine metabolism | ***-3.07870*** | 0.00283 |
| 251.1018 | 7.4768 | C10H13N5O3 | Deoxyadenosine | 6 | Nucleotide Metabolism | Purine metabolism | -1.11070 | 0.00398 |
| 227.0908 | 10.0043 | C9H13N3O4 | Deoxycytidine | 8 | Nucleotide Metabolism | Pyrimidine metabolism | -2.23430 | 0.00001 |
| 402.0231 | 14.6494 | C10H16N2O11P2 | dTDP | 6 | Nucleotide Metabolism | Pyrimidine metabolism | ***-2.81840*** | 0.00540 |
| 481.9895 | 16.3499 | C10H17N2O14P3 | dTTP | 8 | Nucleotide Metabolism | Pyrimidine metabolism | ***-3.61920*** | 0.00322 |
| 443.0247 | 18.4040 | C10H15N5O11P2 | GDP | 8 | Nucleotide Metabolism | Purine metabolism | ***-3.20780*** | 0.00091 |
| 363.0583 | 16.8633 | C10H14N5O8P | GMP | 6 | Nucleotide Metabolism | Purine metabolism, Glutamate metabolism | ***-2.06760*** | 0.00005 |
| 522.9913 | 20.5619 | C10H16N5O14P3 | GTP | 6 | Nucleotide Metabolism | Purine metabolism, Riboflavin metabolism, Folate biosynthesis | ***-2.28500*** | 0.00359 |
| 283.0918 | 12.4403 | C10H13N5O5 | Guanosine | 8 | Nucleotide Metabolism | Purine metabolism | -0.64912 | 0.03306 |
| 136.0385 | 9.7264 | C5H4N4O | Hypoxanthine | 8 | Nucleotide Metabolism | Purine metabolism | -2.67300 | 0.00163 |
| 176.0433 | 17.3792 | C5H8N2O5 | N-Carbamoyl-L-aspartate | 8 | Nucleotide Metabolism | Pyrimidine metabolism, Alanine and aspartate metabolism | ***-1.92310*** | 0.01398 |
| 126.0429 | 7.0906 | C5H6N2O2 | Thymine | 6 | Nucleotide Metabolism | Pyrimidine metabolism | 0.78996 | 0.00009 |
| 404.0023 | 16.8160 | C9H14N2O12P2 | UDP | 6 | Nucleotide Metabolism | Pyrimidine metabolism, Peptidoglycan biosynthesis, Zeatin biosynthesis | ***-4.15790*** | 0.00004 |
| 324.0360 | 15.2943 | C9H13N2O9P | UMP | 6 | Nucleotide Metabolism | Pyrimidine metabolism, Peptidoglycan biosynthesis | -1.83540 | 0.00012 |
| 112.0273 | 9.5781 | C4H4N2O2 | Uracil | 8 | Nucleotide Metabolism | Pyrimidine metabolism, beta-Alanine metabolism, Pantothenate and CoA biosynthesis | ***1.04600*** | 0.00077 |
| 168.0284 | 27.5088 | C5H4N4O3 | Urate | 6 | Nucleotide Metabolism | Purine metabolism | 0.93136 | 0.00182 |
| 244.0696 | 9.6448 | C9H12N2O6 | Uridine | 5 | Nucleotide Metabolism | Pyrimidine metabolism | 1.18550 | 0.00311 |
| 483.9689 | 18.9324 | C9H15N2O15P3 | UTP | 8 | Nucleotide Metabolism | Pyrimidine metabolism | ***-3.04010*** | 0.00002 |
| 280.1059 | 11.0194 | C13H16N2O5 | Phe-Asp | 7 | Peptide(di-) | Hydrophobic peptide | -0.89458 | 0.00088 |
| 222.1001 | 10.1135 | C11H14N2O3 | Phe-Gly | 5 | Peptide(di-) | Hydrophobic peptide | -2.78870 | 0.00000 |
| 216.1472 | 11.8713 | C10H20N2O3 | Val-Val | 5 | Peptide(di-) | Hydrophobic peptide | -1.09880 | 0.00005 |
| 415.1704 | 10.2975 | C16H25N5O8 | Ala-Asn-Asp-Pro | 5 | Peptide(tetra-) | Acidic peptide | -3.66940 | 0.00115 |
| 488.2056 | 8.5534 | C19H32N6O7S | Asn-Met-Gln-Pro | 7 | Peptide(tetra-) | Hydrophobic peptide | -0.66062 | 0.00641 |
| 386.1805 | 11.6589 | C16H26N4O7 | Asp-Val-Gly-Pro | 5 | Peptide(tetra-) | Hydrophobic peptide | -2.43140 | 0.00000 |
| 261.0963 | 15.4586 | C9H15N3O6 | Ala-Asp-Gly | 5 | Peptide(tri-) | Acidic peptide | -1.42590 | 0.00062 |
| 301.1275 | 7.1654 | C12H19N3O6 | Ala-Asp-Pro | 5 | Peptide(tri-) | Acidic peptide | -1.43120 | 0.00307 |
| 193.0409 | 8.2321 | C6H11NO4S | &gamma;-thiomethyl glutamate | 7 | Undefined | Undefined | -2.52570 | 0.00007 |
| 275.1003 | 15.9415 | C11H17NO7 | 1,6-anhydro-N-acetylmuramate | 6 | Undefined | Undefined | ***-2.03350*** | 0.00050 |
| 197.1206 | 29.3319 | C14H15N | 2-(3-Phenylpropyl)pyridine | 5 | Undefined | Undefined | 0.62797 | 0.00141 |
| 160.0920 | 27.6285 | C8H16OS | 2-Methyl-4-propyl-1,3-oxathiane | 5 | Undefined | Undefined | 0.77100 | 0.00127 |
| 196.0961 | 7.2974 | C8H12N4O2 | 3,5-dihydro-5-methylidene-4H-imidazol-4-one | 5 | Undefined | Undefined | -2.44400 | 0.00000 |
| 286.2511 | 3.4824 | C17H34O3 | 3-Hydroxy-palmitic acid methyl ester | 5 | Undefined | Undefined | 1.60310 | 0.00391 |
| 297.2667 | 3.5867 | C18H35NO2 | 3-Ketosphingosine | 5 | Undefined | Undefined | 1.55300 | 0.00076 |
| 184.1099 | 4.4760 | C10H16O3 | 5-exo-Hydroxy-1,2-campholide | 7 | Undefined | Undefined | 0.99521 | 0.00713 |
| 234.1003 | 7.1024 | C12H14N2O3 | 5-Methoxytryptophan | 5 | Undefined | Undefined | -0.65509 | 0.00063 |
| 403.0881 | 15.4440 | C12H22NO12P | 6-(alpha-D-Glucosaminyl)-1D-myo-inositol 1,2-cyclic phosphate | 5 | Undefined | Undefined | -2.42900 | 0.00000 |
| 220.0519 | 13.5547 | C7H12N2O4S | AMCC | 7 | Undefined | Undefined | -2.80700 | 0.00000 |
| 222.0892 | 27.2377 | C12H14O4 | Apiole | 5 | Undefined | Undefined | 0.63498 | 0.00610 |
| 117.9359 | 21.6031 | CrH2O4 | chromate | 7 | Undefined | Undefined | 0.76324 | 0.00030 |
| 541.0603 | 13.5679 | C15H21N5O13P2 | Cyclic ADP-ribose | 5 | Undefined | Undefined | -2.24370 | 0.00003 |
| 453.0282 | 13.5401 | C22H13Cl2N3O4 | dichlorochromopyrrolate | 5 | Undefined | Undefined | -1.73640 | 0.02451 |
| 244.1059 | 8.7335 | C10H16N2O5 | dihydrothymidine | 7 | Undefined | Undefined | -2.42510 | 0.00000 |
| 846.6651 | 27.9515 | C55H91O4P | di-trans,poly-cis-undecaprenyl phosphate | 8 | Undefined | teichoic acid (poly-glycerol) biosynthesis, peptidoglycan biosynthesis I, peptidoglycan biosynthesis II, enterobacterial common antigen biosynthesis, biosynthesis of 4-amino-4-deoxy-L-arabinose-modified lipid A | ***3.92260*** | 0.00079 |
| 246.0506 | 13.0359 | C6H15O8P | Glycerophosphoglycerol | 7 | Undefined | Undefined | -2.38620 | 0.00000 |
| 101.1205 | 11.6123 | C6H15N | Hexylamine | 7 | Undefined | Undefined | 0.59822 | 0.00117 |
| 100.0273 | 27.1254 | C3H4N2O2 | Hydantoin | 6 | Undefined | Undefined | 0.68400 | 0.00078 |
| 98.0480 | 27.1602 | C4H6N2O | Imidazole-4-methanol | 5 | Undefined | Undefined | 0.70167 | 0.00116 |
| 231.1105 | 4.7214 | C10H17NO5 | Isovalerylglutamicacid | 7 | Undefined | Undefined | -2.52980 | 0.00000 |
| 328.2980 | 3.3582 | C20H40O3 | L-2-Hydroxyphytanate | 7 | Undefined | Undefined | 1.57150 | 0.00065 |
| 260.1008 | 7.1124 | C10H16N2O6 | L-alpha-glutamyl-L-hydroxyproline | 5 | Undefined | Undefined | -2.79190 | 0.00001 |
| 228.1474 | 7.8000 | C11H20N2O3 | L-isoleucyl-L-proline | 7 | Undefined | Undefined | 0.72917 | 0.01122 |
| 266.1383 | 5.1403 | C12H18N4O3 | L-lysine-p-nitroanilide | 7 | Undefined | Undefined | -2.42930 | 0.00000 |
| 434.0953 | 13.5712 | C13H19N6O9P | L-seryl-AMP | 7 | Undefined | Undefined | -2.52990 | 0.00000 |
| 133.0198 | 8.6936 | C4H7NO2S | L-thiazolidine-4-carboxylate | 7 | Undefined | Undefined | -2.00330 | 0.00028 |
| 95.9879 | 27.1581 | CH4O3S | Methanesulfonic acid | 7 | Undefined | dimethylsulfide degradation II (oxidation), methanesulfonate degradation | 0.68162 | 0.00023 |
| 106.0450 | 4.3287 | C4H10OS | methionol | 6 | Undefined | methionine degradation III | 1.68420 | 0.00664 |
| 213.1110 | 13.6555 | C9H15N3O3 | Methyl 2-diazoacetamidohexonate | 7 | Undefined | Undefined | -0.69138 | 0.03495 |
| 439.2357 | 8.3343 | C26H33NO5 | Militarinone B | 5 | Undefined | Undefined | -0.96580 | 0.00114 |
| 223.1056 | 12.7078 | C8H17NO6 | N-acetyl -D- glucosaminitol | 7 | Undefined | Undefined | -2.55380 | 0.00000 |
| 205.0952 | 9.8011 | C8H15NO5 | N-Acetyl-D-fucosamine | 5 | Undefined | Undefined | -0.73635 | 0.01024 |
| 237.0850 | 13.2638 | C8H15NO7 | N-Acetyl-D-glucosaminate | 5 | Undefined | Undefined | ***-2.67430*** | 0.00000 |
| 246.1009 | 7.1333 | C13H14N2O3 | N-Acetyl-D-tryptophan | 5 | Undefined | Undefined | -2.70710 | 0.00000 |
| 235.0692 | 15.7598 | C8H13NO7 | N-Acetylgalactosaminate | 5 | Undefined | Undefined | -0.64023 | 0.01193 |
| 191.0617 | 5.4806 | C7H13NO3S | N-Acetylmethionine | 7 | Undefined | Undefined | -0.99874 | 0.01503 |
| 293.1110 | 10.7185 | C11H19NO8 | N-Acetylmuramate | 8 | Undefined | Undefined | ***-2.47370*** | 0.00000 |
| 287.1121 | 7.8367 | C11H17N3O6 | N-Ribosylhistidine | 5 | Undefined | Undefined | -2.29080 | 0.02519 |
| 129.1518 | 14.6571 | C8H19N | Octylamine | 7 | Undefined | Undefined | 3.34240 | 0.00003 |
| 264.0899 | 4.5308 | C16H12N2O2 | Perlolyrine | 5 | Undefined | Undefined | 1.96900 | 0.00016 |
| 374.1723 | 7.4983 | C21H26O6 | Phantomolin | 5 | Undefined | Undefined | -2.12050 | 0.00000 |
| 221.0358 | 14.0798 | C7H11NO5S | S-(3-oxo-3-carboxy-n-propyl)cysteine | 5 | Undefined | Undefined | -3.29650 | 0.00008 |
| 249.0861 | 15.6898 | C10H19NO2S2 | S-Acetyldihydrolipoamide | 5 | Undefined | Alanineandaspartatemetabolism | -2.66250 | 0.00187 |
| 400.1070 | 8.2218 | C13H25N2O8PS | S-Acetylphosphopantetheine | 7 | Undefined | Undefined | 0.94034 | 0.00684 |
| 3 hr Metabolites | | | | | | | | |
| Mass | **RT** | **Formula** | **Putative metabolite** | **Confidence** | **MAP** | **Pathway** | **Log2-fold change** | **FDR adjusted *p*-value** |
| 148.0372 | 15.7353 | C5H8O5 | (R)-2-Hydroxyglutarate | 8 | Amino Acid Metabolism | glutamate degradation V (via hydroxyglutarate) | ***-2.54440*** | 0.00003 |
| 113.0477 | 8.5001 | C5H7NO2 | (S)-1-Pyrroline-5-carboxylate | 8 | Amino Acid Metabolism | Glutamate metabolism, Arginine and proline metabolism | -2.05010 | 0.00001 |
| 146.0579 | 3.5035 | C6H10O4 | (S)-2-Aceto-2-hydroxybutanoate | 6 | Amino Acid Metabolism | Valine, leucine and isoleucine biosynthesis | 2.09410 | 0.00080 |
| 160.0372 | 10.2934 | C6H8O5 | 2-Oxoadipate | 6 | Amino Acid Metabolism | Lysine biosynthesis, Lysine degradation, Tryptophan metabolism | 1.19860 | 0.00074 |
| 180.0899 | 9.4283 | C9H12N2O2 | 3-Hydroxykynurenamine | 5 | Amino Acid Metabolism | Tryptophan metabolism | -2.64410 | 0.00004 |
| 116.0473 | 7.0732 | C5H8O3 | 3-Methyl-2-oxobutanoic acid | 8 | Amino Acid Metabolism | Valine, leucine and isoleucine degradation, Valine, leucine and isoleucine biosynthesis, Pantothenate and CoA biosynthesis | ***1.44610*** | 0.00400 |
| 851.1713 | 9.5834 | C26H44N7O17P3S | 3-Methylbutanoyl-CoA | 8 | Amino Acid Metabolism | Valine, leucine and isoleucine degradation | -2.64630 | 0.00000 |
| 87.0684 | 4.8882 | C4H9NO | 4-Aminobutanal | 8 | Amino Acid Metabolism | Arginine and proline metabolism, beta-Alanine metabolism | 0.96887 | 0.00236 |
| 145.0375 | 8.9960 | C5H7NO4 | 4-Oxoglutaramate | 6 | Amino Acid Metabolism | Histidine metabolism | ***3.16590*** | 0.00002 |
| 129.0426 | 10.4948 | C5H7NO3 | 4-Oxoproline | 7 | Amino Acid Metabolism | Arginine and proline metabolism | -0.59648 | 0.00002 |
| 131.0583 | 13.7925 | C5H9NO3 | 5-Aminolevulinate | 8 | Amino Acid Metabolism | Glycine, serine and threonine metabolism, Porphyrin and chlorophyll metabolism | 0.61156 | 0.00044 |
| 160.0848 | 11.0998 | C6H12N2O3 | D-Alanyl-D-alanine | 8 | Amino Acid Metabolism | D-Alanine metabolism, Peptidoglycan biosynthesis | -0.62660 | 0.00409 |
| 259.0457 | 16.3549 | C6H14NO8P | D-Glucosamine 6-phosphate | 8 | Amino Acid Metabolism | Glutamate metabolism__Aminosugars metabolism | ***-2.54890*** | 0.00000 |
| 142.0742 | 12.5569 | C6H10N2O2 | Ectoine | 5 | Amino Acid Metabolism | Glycine, serine and threonine metabolism | -1.70890 | 0.00002 |
| 229.0885 | 14.6993 | C9H15N3O2S | Ergothioneine | 7 | Amino Acid Metabolism | Histidine metabolism | ***0.65400*** | 0.00535 |
| 199.0959 | 11.5965 | C8H13N3O3 | gamma-Glutamyl-beta-aminopropiononitrile | 5 | Amino Acid Metabolism | Cyanoamino acid metabolism | -4.03100 | 0.00001 |
| 216.1108 | 8.5437 | C9H16N2O4 | gamma-Glutamyl-gamma-aminobutyraldehyde | 7 | Amino Acid Metabolism | Arginine and proline metabolism | -0.84628 | 0.01202 |
| 117.0539 | 15.7490 | C3H7N3O2 | Guanidinoacetate | 7 | Amino Acid Metabolism | Glycine, serine and threonine metabolism__Arginine and proline metabolism | -1.20250 | 0.00039 |
| 117.0577 | 3.9158 | C8H7N | Indole | 8 | Amino Acid Metabolism | Tryptophan metabolism__Phenylalanine, tyrosine and tryptophan biosynthesis__Benzoxazinone biosynthesis | 1.09250 | 0.00007 |
| 161.0688 | 9.6548 | C6H11NO4 | L-2-Aminoadipate | 8 | Amino Acid Metabolism | Lysine biosynthesis__Lysine degradation__Penicillin and cephalosporin biosynthesis | 1.28760 | 0.00001 |
| 89.0478 | 14.5914 | C3H7NO2 | L-Alanine | 8 | Amino Acid Metabolism | Alanine and aspartate metabolism__Cysteine metabolism__Taurine and hypotaurine metabolism__Selenoamino acid metabolism__D-Alanine metabolism__Carbon fixation__Reductive carboxylate cycle (CO2 fixation) | ***-1.06410*** | 0.00015 |
| 117.0426 | 9.6439 | C4H7NO3 | L-Aspartate 4-semialdehyde | 6 | Amino Acid Metabolism | Glycine, serine and threonine metabolism__Lysine biosynthesis | 1.29810 | 0.00000 |
| 175.0957 | 15.6479 | C6H13N3O3 | L-Citrulline | 8 | Amino Acid Metabolism | Arginine and proline metabolism | ***-3.44050*** | 0.00001 |
| 169.0046 | 16.3292 | C3H7NO5S | L-Cysteate | 8 | Amino Acid Metabolism | Cysteine metabolism__Taurine and hypotaurine metabolism | -2.71960 | 0.00002 |
| 121.0197 | 8.2078 | C3H7NO2S | L-Cysteine | 6 | Amino Acid Metabolism | Glycine, serine and threonine metabolism__Methionine metabolism__Cysteine metabolism__Penicillin and cephalosporin biosynthesis__Taurine and hypotaurine metabolism__Glutathione metabolism__Thiamine metabolism__Pantothenate and CoA biosynthesis | ***0.63482*** | 0.01161 |
| 163.0481 | 12.6491 | C5H9NO5 | L-erythro-4-Hydroxyglutamate | 7 | Amino Acid Metabolism | Arginine and proline metabolism | 1.34100 | 0.00032 |
| 146.0692 | 14.8369 | C5H10N2O3 | L-Glutamine | 8 | Amino Acid Metabolism | Glutamate metabolism__Purine metabolism__Pyrimidine metabolism__D-Glutamine and D-glutamate metabolism__Nitrogen metabolism | ***-1.29940*** | 0.00457 |
| 155.0695 | 14.7300 | C6H9N3O2 | L-Histidine | 8 | Amino Acid Metabolism | Histidine metabolism__beta-Alanine metabolism | ***-0.95055*** | 0.00290 |
| 190.0953 | 21.9441 | C7H14N2O4 | LL-2,6-Diaminoheptanedioate | 6 | Amino Acid Metabolism | Lysine biosynthesis | -2.34360 | 0.01413 |
| 131.0946 | 10.7587 | C6H13NO2 | L-Leucine | 6 | Amino Acid Metabolism | Valine, leucine and isoleucine degradation__Valine, leucine and isoleucine biosynthesis | 0.84420 | 0.00010 |
| 146.1055 | 21.9485 | C6H14N2O2 | L-Lysine | 8 | Amino Acid Metabolism | Lysine biosynthesis__Lysine degradation__Biotin metabolism__Alkaloid biosynthesis II | ***-1.60900*** | 0.00006 |
| 132.0899 | 21.2008 | C5H12N2O2 | L-Ornithine | 8 | Amino Acid Metabolism | Arginine and proline metabolism__D-Arginine and D-ornithine metabolism__Glutathione metabolism | ***-2.34710*** | 0.00003 |
| 105.0427 | 15.7536 | C3H7NO3 | L-Serine | 8 | Amino Acid Metabolism | Glycine, serine and threonine metabolism__Methionine metabolism__Cysteine metabolism__Cyanoamino acid metabolism__Sphingolipid metabolism__Methane metabolism__Sulfur metabolism | ***0.74772*** | 0.00004 |
| 119.0582 | 14.7210 | C4H9NO3 | L-Threonine | 8 | Amino Acid Metabolism | Glycine, serine and threonine metabolism__Valine, leucine and isoleucine biosynthesis__Porphyrin and chlorophyll metabolism | 3.71760 | 0.00000 |
| 181.0740 | 12.8787 | C9H11NO3 | L-Tyrosine | 8 | Amino Acid Metabolism | Tyrosine metabolism__Phenylalanine, tyrosine and tryptophan biosynthesis__Novobiocin biosynthesis__Thiamine metabolism__Phenylpropanoid biosynthesis__Alkaloid biosynthesis I | -0.95965 | 0.00035 |
| 290.1229 | 16.6755 | C10H18N4O6 | N-(L-Arginino)succinate | 8 | Amino Acid Metabolism | Arginine and proline metabolism__Alanine and aspartate metabolism | ***-3.01050*** | 0.00000 |
| 218.1266 | 8.3759 | C9H18N2O4 | N2-(D-1-Carboxyethyl)-L-lysine | 7 | Amino Acid Metabolism | Lysine degradation | -2.29290 | 0.00044 |
| 232.1058 | 8.8266 | C9H16N2O5 | N2-Succinyl-L-ornithine | 6 | Amino Acid Metabolism | Arginine and proline metabolism | 0.95379 | 0.00133 |
| 188.1161 | 14.4520 | C8H16N2O3 | N6-Acetyl-L-lysine | 7 | Amino Acid Metabolism | Lysine degradation | -1.74360 | 0.00001 |
| 204.1110 | 12.7682 | C8H16N2O4 | N6-Acetyl-N6-hydroxy-L-lysine | 7 | Amino Acid Metabolism | Lysine degradation | -2.53770 | 0.00364 |
| 221.0902 | 11.1817 | C8H15NO6 | N-Acetyl-D-glucosamine | 6 | Amino Acid Metabolism | Glutamate metabolism__Aminosugars metabolism | ***-2.99780*** | 0.00001 |
| 217.1062 | 11.0140 | C8H15N3O4 | N-Acetyl-L-citrulline | 6 | Amino Acid Metabolism | arginine biosynthesis III | ***-2.64000*** | 0.00000 |
| 189.0637 | 14.4359 | C7H11NO5 | N-Acetyl-L-glutamate | 8 | Amino Acid Metabolism | Arginine and proline metabolism | -1.04750 | 0.00289 |
| 174.1005 | 9.7524 | C7H14N2O3 | N-Acetylornithine | 8 | Amino Acid Metabolism | Arginine and proline metabolism | ***-2.37190*** | 0.00000 |
| 130.1106 | 7.1747 | C6H14N2O | N-Acetylputrescine | 6 | Amino Acid Metabolism | Arginine and proline metabolism | -0.86244 | 0.00611 |
| 190.0590 | 16.9523 | C6H10N2O5 | N-Carbamyl-L-glutamate | 7 | Amino Acid Metabolism | Histidine metabolism | ***0.72900*** | 0.00038 |
| 174.0640 | 14.6705 | C6H10N2O4 | N-Formimino-L-glutamate | 8 | Amino Acid Metabolism | Histidine metabolism | ***-2.30850*** | 0.00792 |
| 177.0459 | 8.3939 | C6H11NO3S | N-Formyl-L-methionine | 7 | Amino Acid Metabolism | Methionine metabolism | ***2.68620*** | 0.00063 |
| 199.0247 | 16.0295 | C4H10NO6P | O-Phospho-L-homoserine | 8 | Amino Acid Metabolism | Glycine, serine and threonine metabolism | ***-1.89950*** | 0.00668 |
| 185.0089 | 16.6012 | C3H8NO6P | O-Phospho-L-serine | 8 | Amino Acid Metabolism | Glycine, serine and threonine metabolism__Cysteine metabolism | ***1.24370*** | 0.00250 |
| 219.1106 | 8.6430 | C9H17NO5 | Pantothenate | 8 | Amino Acid Metabolism | beta-Alanine metabolism__Pantothenate and CoA biosynthesis | ***-1.56970*** | 0.00001 |
| 150.0680 | 4.6761 | C9H10O2 | Phenylpropanoate | 5 | Amino Acid Metabolism | Phenylalanine metabolism | 1.03900 | 0.01417 |
| 164.0474 | 4.6798 | C9H8O3 | Phenylpyruvate | 8 | Amino Acid Metabolism | Phenylalanine metabolism__Phenylalanine, tyrosine and tryptophan biosynthesis | 1.64170 | 0.00779 |
| 123.9925 | 13.7650 | C2H5O4P | Phosphonoacetaldehyde | 6 | Amino Acid Metabolism | Aminophosphonate metabolism | -2.44510 | 0.00735 |
| 750.1397 | 15.5757 | C23H36N4O20P2 | UDP-N-acetylmuramoyl-L-alanine | 8 | Amino Acid Metabolism | D-Glutamine and D-glutamate metabolism__Peptidoglycan biosynthesis | ***4.19090*** | 0.00033 |
| 138.0430 | 28.7932 | C6H6N2O2 | Urocanate | 8 | Amino Acid Metabolism | Histidine metabolism | ***0.74135*** | 0.00000 |
| 156.0535 | 11.4999 | C6H8N2O3 | 4-Imidazolone-5-propanoate | 8 | Amino Acid Metabolism | Histidine metabolism | ***-2.76260*** | 0.00002 |
| 147.0531 | 14.8480 | C5H9NO4 | L-Glutamate | 8 | Amino Acid Metabolism | Arginine and proline metabolism__Glutamate metabolism__Histidine metabolism__D-Glutamine and D-glutamate metabolism__Glutathione metabolism_Butanoate metabolism_C5-Branched dibasic acid metabolism_Porphyrin and chlorophyll metabolism_Nitrogen metabolism | ***-1.29940*** | 0.00457 |
| 398.0997 | 17.4363 | C21H18O8 | Dihydro-NAME | 7 | Biosynthesis of Polyketides and Nonribosomal Peptides | Biosynthesis of type II polyketide products | -2.46400 | 0.00000 |
| 155.0582 | 8.8894 | C7H9NO3 | (3S,5R)-carbapenam | 7 | Biosynthesis of Secondary Metabolites | (5R)-carbapenem biosynthesis | -2.28580 | 0.00003 |
| 134.0216 | 16.7004 | C4H6O5 | (S)-Malate | 8 | Carbohydrate Metabolism | Citrate cycle (TCA cycle)__Glutamate metabolism__Alanine and aspartate metabolism__Pyruvate metabolism__Glyoxylate and dicarboxylate metabolism__Carbon fixation__Reductive carboxylate cycle (CO2 fixation) | ***-1.52410*** | 0.00000 |
| 136.0372 | 13.0123 | C4H8O5 | [FA trihydroxy(4:0)] 2,3,4-trihydroxy-butanoic acid | 8 | Carbohydrate Metabolism | Ascorbate and aldarate metabolism | -1.44870 | 0.00028 |
| 162.0528 | 13.1369 | C6H10O5 | 3-Ethylmalate | 7 | Carbohydrate Metabolism | Glyoxylate and dicarboxylate metabolism | -2.91960 | 0.00001 |
| 185.9929 | 17.4534 | C3H7O7P | 3-Phospho-D-glycerate | 8 | Carbohydrate Metabolism | Glycolysis / Gluconeogenesis__Glycine, serine and threonine metabolism__Glycerolipid metabolism__Glyoxylate and dicarboxylate metabolism__Carbon fixation | ***-1.85910*** | 0.00474 |
| 177.0637 | 7.8317 | C6H11NO5 | 4-Hydroxy-4-methylglutamate | 7 | Carbohydrate Metabolism | C5-Branched dibasic acid metabolism | 3.67730 | 0.00003 |
| 158.0689 | 12.9674 | C6H10N2O3 | 4-Methylene-L-glutamine | 5 | Carbohydrate Metabolism | C5-Branched dibasic acid metabolism | -1.54830 | 0.00004 |
| 274.0091 | 16.3983 | C6H11O10P | 6-Phospho-2-dehydro-D-gluconate | 8 | Carbohydrate Metabolism | Pentose phosphate pathway | 2.23380 | 0.00063 |
| 422.0827 | 16.8223 | C12H23O14P | alpha,alpha'-Trehalose 6-phosphate | 7 | Carbohydrate Metabolism | Starch and sucrose metabolism | ***-1.71900*** | 0.00168 |
| 272.0890 | 23.2226 | C12H16O7 | Arbutin | 5 | Carbohydrate Metabolism | Glycolysis / Gluconeogenesis | -0.65149 | 0.00392 |
| 196.0583 | 16.0945 | C6H12O7 | D-Gluconic acid | 8 | Carbohydrate Metabolism | Pentose phosphate pathway | ***-3.45790*** | 0.00001 |
| 178.0478 | 12.3723 | C6H10O6 | D-Glucono-1,5-lactone | 8 | Carbohydrate Metabolism | Pentose phosphate pathway | ***-0.89839*** | 0.00023 |
| 179.0794 | 16.2658 | C6H13NO5 | D-Glucosamine | 5 | Carbohydrate Metabolism | Aminosugars metabolism | ***-1.85610*** | 0.00207 |
| 180.0634 | 12.4954 | C6H12O6 | D-Glucose | 8 | Carbohydrate Metabolism | Glycolysis / Gluconeogenesis__Pentose phosphate pathway__Galactose metabolism__Starch and sucrose metabolism__Streptomycin biosynthesis__Indole and ipecac alkaloid biosynthesis | ***-3.87220*** | 0.00001 |
| 260.0297 | 16.2538 | C6H13O9P | D-Glucose 6-phosphate | 6 | Carbohydrate Metabolism | Starch and sucrose metabolism__Streptomycin biosynthesis__Inositol phosphate metabolism | -0.76016 | 0.01025 |
| 86.0370 | 3.5901 | C4H6O2 | Diacetyl | 8 | Carbohydrate Metabolism | Butanoate metabolism | 2.16810 | 0.00103 |
| 232.0348 | 15.6721 | C5H13O8P | D-Ribitol 5-phosphate | 7 | Carbohydrate Metabolism | Pentose and glucuronate interconversions | ***1.70200*** | 0.00000 |
| 169.9980 | 16.3988 | C3H7O6P | Glycerone phosphate | 8 | Carbohydrate Metabolism | Glycolysis / Gluconeogenesis__Inositol metabolism__Pentose and glucuronate interconversions__Fructose and mannose metabolism__Galactose metabolism__Glycerolipid metabolism__Glycerophospholipid metabolism__Pyruvate metabolism__Carbon fixation | ***2.76600*** | 0.00015 |
| 152.0684 | 12.8558 | C5H12O5 | L-Arabitol | 8 | Carbohydrate Metabolism | Pentose and glucuronate interconversions | 1.51070 | 0.00002 |
| 164.0685 | 10.8091 | C6H12O5 | L-Rhamnofuranose | 8 | Carbohydrate Metabolism | Fructose and mannose metabolism | 4.16180 | 0.00088 |
| 666.2223 | 17.3376 | C24H42O21 | Maltotetraose | 8 | Carbohydrate Metabolism | glycogen degradation I | -1.24320 | 0.00154 |
| 504.1696 | 16.3168 | C18H32O16 | Maltotriose | 6 | Carbohydrate Metabolism | glycogen degradation I | -1.63950 | 0.00000 |
| 182.0791 | 13.8198 | C6H14O6 | Mannitol | 8 | Carbohydrate Metabolism | Fructose and mannose metabolism | 2.03020 | 0.00034 |
| 309.1062 | 13.4556 | C11H19NO9 | N-Acetylneuraminate | 6 | Carbohydrate Metabolism | Aminosugars metabolism | 0.96690 | 0.00922 |
| 167.9824 | 16.8761 | C3H5O6P | Phosphoenolpyruvate | 6 | Carbohydrate Metabolism | Glycolysis / Gluconeogenesis__Citrate cycle (TCA cycle)__Phenylalanine, tyrosine and tryptophan biosynthesis__Aminophosphonate metabolism__Pyruvate metabolism__Carbon fixation__Reductive carboxylate cycle (CO2 fixation) | ***-1.64270*** | 0.01815 |
| 154.0031 | 12.0185 | C3H7O5P | Propanoyl phosphate | 8 | Carbohydrate Metabolism | Propanoate metabolism__C5-Branched dibasic acid metabolism | 2.33770 | 0.00000 |
| 88.0160 | 8.7945 | C3H4O3 | Pyruvate | 8 | Carbohydrate Metabolism | Glycolysis / Gluconeogenesis__Citrate cycle (TCA cycle)__Pentose phosphate pathway__Ascorbate and aldarate metabolism__Biosynthesis of steroids__Alanine and aspartate metabolism__Glycine, serine and threonine metabolism | ***0.85513*** | 0.00015 |
| 118.0266 | 15.6987 | C4H6O4 | Succinate | 8 | Carbohydrate Metabolism | Citrate cycle (TCA cycle)__Oxidative phosphorylation__Glutamate metabolism__Alanine and aspartate metabolism__Tyrosine metabolism__Phenylalanine metabolism__gamma-Hexachlorocyclohexane degradation__Glyoxylate and dicarboxylate metabolism | ***-1.03820*** | 0.00000 |
| 867.1307 | 15.5102 | C25H40N7O19P3S | Succinyl-CoA | 8 | Carbohydrate Metabolism | Citrate cycle (TCA cycle)__Valine, leucine and isoleucine degradation__Benzoate degradation via hydroxylation__1- and 2-Methylnaphthalene degradation__Benzoate degradation via CoA ligation__Propanoate metabolism__Reductive carboxylate cycle (CO2 fixation) | ***-2.29880*** | 0.00860 |
| 427.0297 | 16.3139 | C10H15N5O10P2 | ADP | 8 | Energy Metabolism | Oxidative phosphorylation__Photosynthesis__Purine metabolism__Zeatin biosynthesis | -0.69351 | 0.01708 |
| 506.9962 | 16.8687 | C10H16N5O13P3 | ATP | 8 | Energy Metabolism | Oxidative phosphorylation__Photosynthesis__Purine metabolism__Puromycin biosynthesis__Zeatin biosynthesis | ***-1.97140*** | 0.00088 |
| 97.9769 | 16.3546 | H3O4P | Orthophosphate | 7 | Energy Metabolism | Oxidative phosphorylation__Photosynthesis__Peptidoglycan biosynthesis | ***-1.73210*** | 0.00083 |
| 238.0690 | 9.8676 | C8H14O8 | 3-Deoxy-D-manno-octulosonate | 8 | Energy Metabolism | Lipopolysaccharide biosynthesis | -2.54830 | 0.00000 |
| 574.6761 | 15.4063 | C40H65N9O26P2 | UDPMurAc(oyl-L-Ala-D-gamma-Glu-L-Lys-D-Ala-D-Ala) | 8 | Glycan Biosynthesis and Metabolism | Peptidoglycan biosynthesis | ***1.43510*** | 0.00056 |
| 183.0662 | 14.6590 | C5H14NO4P | Choline phosphate | 8 | Lipid Metabolism | Glycerophospholipid metabolism__Glycine, serine and threonine metabolism | ***-3.74470*** | 0.00003 |
| 257.1028 | 13.7581 | C8H20NO6P | sn-glycero-3-Phosphocholine | 8 | Lipid Metabolism | Glycerophospholipid metabolism__Ether lipid metabolism | ***-2.44550*** | 0.00029 |
| 172.0137 | 15.0612 | C3H9O6P | sn-Glycerol 3-phosphate | 8 | Lipid Metabolism | Glycerolipid metabolism__Glycerophospholipid metabolism | -2.02500 | 0.00133 |
| 243.2195 | 3.7940 | C14H29NO2 | [FA amino(14:0)] 2-amino-tetradecanoic acid | 7 | Lipids: Fatty Acyls | Amino Fatty Acids | -0.94820 | 0.00501 |
| 172.1463 | 3.2504 | C10H20O2 | Decanoic acid | 5 | Lipids: Fatty Acyls | Fatty acid biosynthesis | 1.26610 | 0.00317 |
| 312.3030 | 3.2272 | C20H40O2 | FA (20:0) | 5 | Lipids: Fatty Acyls | Fatty Acids and Conjugates | ***3.64180*** | 0.00099 |
| 310.2874 | 3.3011 | C20H38O2 | FA (20:1) | 5 | Lipids: Fatty Acyls | Fatty Acids and Conjugates | ***2.68190*** | 0.00350 |
| 270.2559 | 3.3450 | C17H34O2 | FA (17:0) | 5 | Lipids: Fatty Acyls | Fatty Acids and Conjugates | ***1.39200*** | 0.01505 |
| 187.0845 | 4.6807 | C8H13NO4 | FA hydroxy(4:0) N-(3S-hydroxy-butanoyl)-homoserine lactone | 8 | Lipids: Fatty Acyls | Fatty amides | -0.96870 | 0.00061 |
| 242.2246 | 3.4334 | C15H30O2 | FA methyl(14:0) | 5 | Lipids: Fatty Acyls | Fatty Acids and Conjugates | ***1.24840*** | 0.01106 |
| 298.2872 | 3.2888 | C19H38O2 | FA methyl(18:0) | 5 | Lipids: Fatty Acyls | Fatty Acids and Conjugates | ***2.21450*** | 0.00902 |
| 312.2666 | 3.4622 | C19H36O3 | FA oxo(19:0) | 5 | Lipids: Fatty Acyls | Fatty Acids and Conjugates | ***-1.51360*** | 0.01743 |
| 353.3293 | 3.3768 | C22H43NO2 | N-(11Z-eicosaenoyl)-ethanolamine | 5 | Lipids: Fatty Acyls | Fatty amides | 3.06090 | 0.00856 |
| 171.0896 | 7.2128 | C8H13NO3 | N-Butyryl-L-homoserine lactone | 6 | Lipids: Fatty Acyls | Fatty amides | -0.99628 | 0.00955 |
| 248.1010 | 15.2310 | C9H16N2O6 | [PG (18:0)] 1-octadecanoyl-sn-glycero-3-phospho-(1'-sn-glycerol) | 7 | Lipids: Glycerophospholipids | Glycerophosphoglycerols | -0.85247 | 0.00126 |
| 666.4474 | 3.1386 | C34H67O10P | [PG (28:0)] | 5 | Lipids: Glycerophospholipids | Glycerophosphoglycerols | 1.18490 | 0.01193 |
| 439.2692 | 4.4734 | C20H42NO7P | LysoPC(12:0) | 7 | Lipids: Glycerophospholipids | Glycerophosphocholines | -0.79650 | 0.01374 |
| 470.2648 | 3.4492 | C21H43O9P | PG(15:0/0:0) | 5 | Lipids: Glycerophospholipids | Glycerophosphoglycerols | -0.95608 | 0.00115 |
| 624.4003 | 3.1600 | C31H61O10P | PG(25:0) | 7 | Lipids: Glycerophospholipids | Glycerophosphoglycerols | ***1.72230*** | 0.00247 |
| 680.4633 | 3.1251 | C35H69O10P | PG(29:0) | 5 | Lipids: Glycerophospholipids | Glycerophosphoglycerols | 0.96124 | 0.01429 |
| 694.4788 | 3.1206 | C36H71O10P | PG(30:0) | 5 | Lipids: Glycerophospholipids | Glycerophosphoglycerols | ***1.21270*** | 0.00172 |
| 708.4944 | 3.1173 | C37H73O10P | PG(31:0) | 5 | Lipids: Glycerophospholipids | Glycerophosphoglycerols | 0.82498 | 0.00969 |
| 722.5101 | 3.1145 | C38H75O10P | PG(32:0) | 5 | Lipids: Glycerophospholipids | Glycerophosphoglycerols | 0.74116 | 0.01235 |
| 736.5257 | 3.1161 | C39H77O10P | PG(33:0) | 5 | Lipids: Glycerophospholipids | Glycerophosphoglycerols | 0.89826 | 0.00329 |
| 764.5570 | 3.1184 | C41H81O10P | PG(35:0) | 5 | Lipids: Glycerophospholipids | Glycerophosphoglycerols | 0.80711 | 0.00826 |
| 762.5414 | 3.1122 | C41H79O10P | PG(35:1) | 5 | Lipids: Glycerophospholipids | Glycerophosphoglycerols | 0.85676 | 0.01484 |
| 832.5077 | 12.0140 | C43H77O13P | PI(34:3) | 5 | Lipids: Glycerophospholipids | Glycerophosphoinositols | ***1.51600*** | 0.00033 |
| 521.3469 | 7.0853 | C26H52NO7P | LysoPC(18:1) | 7 | Lipids: Glycerophospholipids | Glycerophosphocholines | ***2.82860*** | 0.00002 |
| 598.4018 | 13.1356 | C40H54O4 | [PR] Ketomyxol/ 2'-Hydroxyflexixanthin | 5 | Lipids: Prenols | Isoprenoids | -0.84763 | 0.00028 |
| 240.0637 | 7.1045 | C11H12O6 | (1R,6R)-6-Hydroxy-2-succinylcyclohexa-2,4-diene-1-carboxylate | 5 | Metabolism of Cofactors and Vitamins | Ubiquinone biosynthesis | 1.68570 | 0.00235 |
| 219.0409 | 12.7258 | C6H10N3O4P | 4-Amino-2-methyl-5-phosphomethylpyrimidine | 6 | Metabolism of Cofactors and Vitamins | Thiamine metabolism | 2.44010 | 0.00000 |
| 326.1228 | 13.1175 | C13H18N4O6 | 6,7-Dimethyl-8-(1-D-ribityl)lumazine | 8 | Metabolism of Cofactors and Vitamins | Riboflavin metabolism | -0.75021 | 0.00003 |
| 139.0269 | 13.9205 | C6H5NO3 | 6-Hydroxynicotinate | 7 | Metabolism of Cofactors and Vitamins | Nicotinate and nicotinamide metabolism | -1.19770 | 0.00011 |
| 188.1525 | 20.9271 | C9H20N2O2 | 7,8-Diaminononanoate | 8 | Metabolism of Cofactors and Vitamins | Biotin metabolism | -1.14530 | 0.00017 |
| 246.1037 | 8.6823 | C10H18N2O3S | 9-mercaptodethiobiotin | 7 | Metabolism of Cofactors and Vitamins | biotin biosynthesis II | -2.62970 | 0.00002 |
| 278.1267 | 10.3730 | C14H18N2O4 | alpha-Ribazole | 8 | Metabolism of Cofactors and Vitamins | Riboflavin metabolism__Porphyrin and chlorophyll metabolism | -2.03480 | 0.00012 |
| 244.0881 | 8.7315 | C10H16N2O3S | Biotin | 6 | Metabolism of Cofactors and Vitamins | Biotin metabolism | 0.66911 | 0.00039 |
| 226.0478 | 15.8936 | C10H10O6 | Chorismate | 6 | Metabolism of Cofactors and Vitamins | Ubiquinone biosynthesis__Phenylalanine, tyrosine and tryptophan biosynthesis__Folate biosynthesis__Biosynthesis of siderophore group nonribosomal peptides | 1.65770 | 0.00030 |
| 214.1317 | 8.9268 | C10H18N2O3 | Dethiobiotin | 8 | Metabolism of Cofactors and Vitamins | Biotin metabolism | -4.04470 | 0.00027 |
| 128.0474 | 7.0732 | C6H8O3 | Dihydro-4,4-dimethyl-2,3-Furandione | 7 | Metabolism of Cofactors and Vitamins | pantothenate and coenzyme A biosynthesis III __ pantothenate biosynthesis II | 3.90890 | 0.00150 |
| 131.0219 | 9.2559 | C4H5NO4 | Iminoaspartate | 8 | Metabolism of Cofactors and Vitamins | Nicotinate and nicotinamide metabolism | 1.13850 | 0.00620 |
| 322.1199 | 7.3870 | C12H22N2O6S | N-((R)-Pantothenoyl)-L-cysteine | 7 | Metabolism of Cofactors and Vitamins | Pantothenate and CoA biosynthesis | ***-2.68180*** | 0.00001 |
| 123.0321 | 7.8712 | C6H5NO2 | Nicotinate | 6 | Metabolism of Cofactors and Vitamins | Nicotinate and nicotinamide metabolism__Alkaloid biosynthesis II | 1.67180 | 0.00032 |
| 358.0966 | 9.8026 | C11H23N2O7PS | Pantetheine 4'-phosphate | 8 | Metabolism of Cofactors and Vitamins | Pantothenate and CoA biosynthesis | 0.72648 | 0.01701 |
| 226.0954 | 7.8604 | C10H14N2O4 | Porphobilinogen | 8 | Metabolism of Cofactors and Vitamins | Porphyrin and chlorophyll metabolism | -0.91177 | 0.00255 |
| 168.0898 | 5.6195 | C8H12N2O2 | Pyridoxamine | 7 | Metabolism of Cofactors and Vitamins | Vitamin B6 metabolism | 0.82567 | 0.01593 |
| 329.0528 | 11.3317 | C10H12N5O6P | 3',5'-Cyclic AMP | 6 | Nucleotide Metabolism | Purine metabolism | -0.92211 | 0.00196 |
| 128.0585 | 13.7965 | C5H8N2O2 | 5,6-Dihydrothymine | 8 | Nucleotide Metabolism | Pyrimidine metabolism | -1.29330 | 0.00006 |
| 314.0517 | 15.2120 | C8H15N2O9P | 5'-Phosphoribosyl-N-formylglycinamide | 8 | Nucleotide Metabolism | Purine metabolism | ***2.48460*** | 0.00001 |
| 135.0545 | 8.5389 | C5H5N5 | Adenine | 8 | Nucleotide Metabolism | Purine metabolism__Zeatin biosynthesis | 0.63407 | 0.00257 |
| 411.0349 | 14.1563 | C10H15N5O9P2 | dADP | 8 | Nucleotide Metabolism | Purine metabolism | 2.96160 | 0.00009 |
| 331.0681 | 12.4574 | C10H14N5O6P | dAMP | 5 | Nucleotide Metabolism | Purine metabolism | 0.68111 | 0.00111 |
| 228.0748 | 7.8149 | C9H12N2O5 | Deoxyuridine | 8 | Nucleotide Metabolism | Pyrimidine metabolism | 1.38880 | 0.00025 |
| 481.9895 | 16.3499 | C10H17N2O14P3 | dTTP | 8 | Nucleotide Metabolism | Pyrimidine metabolism | -1.40440 | 0.00382 |
| 151.0495 | 12.1702 | C5H5N5O | Guanine | 8 | Nucleotide Metabolism | Purine metabolism | ***1.20080*** | 0.00062 |
| 176.0433 | 17.3792 | C5H8N2O5 | N-Carbamoyl-L-aspartate | 8 | Nucleotide Metabolism | Pyrimidine metabolism__Alanine and aspartate metabolism | ***1.57190*** | 0.00004 |
| 102.0430 | 12.1111 | C3H6N2O2 | N-Formiminoglycine | 5 | Nucleotide Metabolism | Purine metabolism | ***1.91520*** | 0.00000 |
| 156.0171 | 10.8314 | C5H4N2O4 | Orotate | 8 | Nucleotide Metabolism | Pyrimidine metabolism | 0.77669 | 0.00932 |
| 126.0429 | 7.0906 | C5H6N2O2 | Thymine | 6 | Nucleotide Metabolism | Pyrimidine metabolism | -1.72150 | 0.00007 |
| 112.0273 | 9.5781 | C4H4N2O2 | Uracil | 8 | Nucleotide Metabolism | Pyrimidine metabolism__beta-Alanine metabolism__Pantothenate and CoA biosynthesis | 0.61116 | 0.00991 |
| 244.0696 | 9.6448 | C9H12N2O6 | Uridine | 5 | Nucleotide Metabolism | Pyrimidine metabolism | 0.65826 | 0.01128 |
| 483.9689 | 18.9324 | C9H15N2O15P3 | UTP | 8 | Nucleotide Metabolism | Pyrimidine metabolism | -1.83980 | 0.00066 |
| 186.1005 | 11.6335 | C8H14N2O3 | Ala-Pro | 5 | Peptide(di-) | Nonpolar peptide | -2.67140 | 0.00000 |
| 229.1064 | 11.2655 | C9H15N3O4 | Asn-Pro | 7 | Peptide(di-) | Polar peptide | -1.78610 | 0.00286 |
| 218.0725 | 7.8703 | C8H14N2O3S | Cys-Pro | 7 | Peptide(di-) | Nonpolar peptide | 0.67106 | 0.00919 |
| 276.0957 | 14.1527 | C10H16N2O7 | Glu-Glu | 7 | Peptide(di-) | Acidic peptide | -2.14190 | 0.01210 |
| 278.0936 | 12.6477 | C10H18N2O5S | Glu-Met | 5 | Peptide(di-) | Hydrophobic peptide | -1.04180 | 0.00832 |
| 202.1318 | 13.0281 | C9H18N2O3 | Ile-Ala | 7 | Peptide(di-) | Hydrophobic peptide | -2.86100 | 0.00001 |
| 259.1895 | 12.6138 | C12H25N3O3 | Ile-Lys | 7 | Peptide(di-) | Basic peptide | -1.21210 | 0.00152 |
| 206.0723 | 11.2711 | C7H14N2O3S | Met-Gly | 5 | Peptide(di-) | Hydrophobic peptide | -1.32300 | 0.00150 |
| 236.0825 | 9.3429 | C8H16N2O4S | Met-Ser | 7 | Peptide(di-) | Hydrophobic peptide | 1.20230 | 0.00012 |
| 222.1001 | 10.1135 | C11H14N2O3 | Phe-Gly | 5 | Peptide(di-) | Hydrophobic peptide | -2.50270 | 0.00000 |
| 216.1472 | 11.8713 | C10H20N2O3 | Val-Val | 5 | Peptide(di-) | Hydrophobic peptide | -0.86065 | 0.00186 |
| 345.1649 | 15.0513 | C13H23N5O6 | Ala-Ala-Ala-Asn | 5 | Peptide(tetra-) | Polar peptide | -2.16180 | 0.00002 |
| 331.1494 | 15.3126 | C12H21N5O6 | Ala-Ala-Asn-Gly | 5 | Peptide(tetra-) | Polar peptide | -1.63680 | 0.00003 |
| 334.1489 | 11.6106 | C12H22N4O7 | Ala-Ala-Ser-Ser | 5 | Peptide(tetra-) | Polar peptide | -0.75423 | 0.00621 |
| 457.2276 | 3.7030 | C18H31N7O7 | Ala-Asp-Pro-Arg | 5 | Peptide(tetra-) | Basic peptide | 1.80250 | 0.00220 |
| 411.2120 | 12.8459 | C18H29N5O6 | Ala-Gln-Pro-Pro | 5 | Peptide(tetra-) | Polar peptide | -3.02730 | 0.00333 |
| 418.1702 | 9.6764 | C16H26N4O9 | Ala-Glu-Ala-Glu | 7 | Peptide(tetra-) | Acidic peptide | -2.69160 | 0.00000 |
| 402.2120 | 9.4028 | C17H30N4O7 | Ala-Glu-Ala-Ile | 5 | Peptide(tetra-) | Hydrophobic peptide | -4.76240 | 0.00041 |
| 414.2841 | 14.4391 | C20H38N4O5 | Ala-Ile-Ile-Val | 5 | Peptide(tetra-) | Hydrophobic peptide | -1.63660 | 0.00016 |
| 330.1908 | 13.0616 | C14H26N4O5 | Ala-Leu-Ala-Gly | 5 | Peptide(tetra-) | Hydrophobic peptide | -2.85760 | 0.00000 |
| 388.2319 | 9.7493 | C17H32N4O6 | Ala-Leu-Val-Ser | 5 | Peptide(tetra-) | Hydrophobic peptide | -2.67950 | 0.00000 |
| 428.2384 | 8.7507 | C18H32N6O6 | Ala-Lys-Asn-Pro | 7 | Peptide(tetra-) | Basic peptide | 1.16440 | 0.00617 |
| 457.2103 | 13.2831 | C18H31N7O5S | Ala-Lys-Cys-His | 7 | Peptide(tetra-) | Basic peptide | -0.59271 | 0.00584 |
| 474.2223 | 8.1396 | C22H30N6O6 | Ala-Phe-Thr-His | 5 | Peptide(tetra-) | Basic peptide | -2.37260 | 0.00000 |
| 360.1645 | 11.6935 | C14H24N4O7 | Ala-Pro-Ser-Ser | 5 | Peptide(tetra-) | Polar peptide | -2.63340 | 0.00000 |
| 495.2443 | 8.4019 | C21H33N7O7 | Ala-Ser-Tyr-Arg | 7 | Peptide(tetra-) | Basic peptide | -3.56810 | 0.00005 |
| 342.1902 | 7.1169 | C15H26N4O5 | Ala-Val-Gly-Pro | 5 | Peptide(tetra-) | Hydrophobic peptide | 3.04580 | 0.00003 |
| 544.2970 | 12.3489 | C22H40N8O8 | Arg-Glu-Gln-Ile | 7 | Peptide(tetra-) | Basic peptide | 2.65020 | 0.00191 |
| 530.3164 | 10.7826 | C22H42N8O7 | Arg-Leu-Lys-Asp | 5 | Peptide(tetra-) | Basic peptide | -0.77696 | 0.00152 |
| 511.2386 | 12.0986 | C21H33N7O8 | Arg-Ser-Ser-Tyr | 7 | Peptide(tetra-) | Basic peptide | -1.02990 | 0.00306 |
| 457.1921 | 9.2304 | C17H27N7O8 | Asn-Asn-Asn-Pro | 5 | Peptide(tetra-) | Polar peptide | -3.33850 | 0.00000 |
| 496.1909 | 10.5307 | C20H28N6O9 | Asn-Asn-Ser-Tyr | 7 | Peptide(tetra-) | Hydrophobic peptide | 1.45450 | 0.00002 |
| 538.2010 | 5.5198 | C22H30N6O10 | Asn-Asp-Gln-Tyr | 7 | Peptide(tetra-) | Hydrophobic peptide | 1.74190 | 0.00001 |
| 485.2237 | 8.4006 | C19H31N7O8 | Asn-Gln-Gln-Pro | 5 | Peptide(tetra-) | Polar peptide | 0.80315 | 0.00247 |
| 509.2298 | 4.6910 | C23H35N5O6S | Asn-Met-Phe-Val | 5 | Peptide(tetra-) | Hydrophobic peptide | 3.56580 | 0.00136 |
| 423.2122 | 4.6293 | C19H29N5O6 | Asn-Pro-Pro-Pro | 5 | Peptide(tetra-) | Polar peptide | 0.87615 | 0.00976 |
| 529.2291 | 8.3032 | C24H31N7O7 | Asn-Pro-Tyr-His | 7 | Peptide(tetra-) | Basic peptide | -3.39890 | 0.00075 |
| 460.1380 | 8.2917 | C16H24N6O8S | Asp-Cys-Ser-His | 5 | Peptide(tetra-) | Basic peptide | 2.44780 | 0.00000 |
| 455.2005 | 10.9706 | C19H29N5O8 | Asp-Gln-Pro-Pro | 5 | Peptide(tetra-) | Acidic peptide | -2.47090 | 0.00000 |
| 458.2019 | 9.6201 | C19H30N4O9 | Asp-Leu-Asp-Pro | 5 | Peptide(tetra-) | Hydrophobic peptide | -3.05110 | 0.00002 |
| 529.1962 | 4.7447 | C20H31N7O8S | Asp-Met-Gln-His | 7 | Peptide(tetra-) | Basic peptide | 1.72500 | 0.00534 |
| 408.1493 | 12.0545 | C14H24N4O10 | Asp-Thr-Ser-Ser | 7 | Peptide(tetra-) | Acidic peptide | 1.66270 | 0.00026 |
| 386.1805 | 11.6589 | C16H26N4O7 | Asp-Val-Gly-Pro | 5 | Peptide(tetra-) | Hydrophobic peptide | -2.73280 | 0.00000 |
| 418.1337 | 11.7691 | C16H26N4O5S2 | Cys-Cys-Pro-Pro | 7 | Peptide(tetra-) | Nonpolar peptide | -5.26710 | 0.00009 |
| 490.1729 | 13.7746 | C19H30N4O9S | Glu-Met-Asp-Pro | 5 | Peptide(tetra-) | Hydrophobic peptide | -2.44620 | 0.00798 |
| 540.1742 | 12.1832 | C20H36N4O7S3 | Glu-Met-Met-Met | 5 | Peptide(tetra-) | Hydrophobic peptide | 1.30210 | 0.00023 |
| 528.3053 | 4.6824 | C27H40N6O5 | His-Ile-Ile-Phe | 5 | Peptide(tetra-) | Basic peptide | 3.86350 | 0.00214 |
| 573.2358 | 12.8769 | C26H35N7O6S | His-Met-Thr-Trp | 5 | Peptide(tetra-) | Basic peptide | -1.24950 | 0.00080 |
| 542.3212 | 4.6774 | C28H42N6O5 | Ile-Lys-Trp-Pro | 5 | Peptide(tetra-) | Basic peptide | 4.26560 | 0.00022 |
| 414.2483 | 8.2642 | C19H34N4O6 | Ile-Val-Pro-Ser | 7 | Peptide(tetra-) | Hydrophobic peptide | 0.90552 | 0.00101 |
| 588.3737 | 13.0020 | C29H48N8O5 | Lys-Lys-Lys-Trp | 5 | Peptide(tetra-) | Basic peptide | -1.43310 | 0.00064 |
| 498.1635 | 12.1611 | C18H34N4O6S3 | Met-Met-Met-Ser | 5 | Peptide(tetra-) | Hydrophobic peptide | 1.43640 | 0.00013 |
| 283.1282 | 9.0329 | C11H17N5O4 | Ala-Gly-His | 5 | Peptide(tri-) | Basic peptide | 1.10030 | 0.00012 |
| 233.1010 | 9.2516 | C8H15N3O5 | Ala-Gly-Ser | 7 | Peptide(tri-) | Polar peptide | -2.74600 | 0.00083 |
| 405.1384 | 9.6317 | C15H23N3O10 | Glu-Glu-Glu | 5 | Peptide(tri-) | Acidic peptide | 1.09920 | 0.00070 |
| 193.0409 | 8.2321 | C6H11NO4S | &gamma;-thiomethyl glutamate | 7 | Undefined | Undefined | 2.21840 | 0.00000 |
| 228.1110 | 8.1137 | C10H16N2O4 | (S)-ATPA | 7 | Undefined | Undefined | -1.07170 | 0.00020 |
| 722.4453 | 7.2455 | C36H66O14 | 13-[O(2')-&beta;-D-glucopyranosyl-&beta;-D-glucopyranosyloxy]docosanoate O(6'')-acetate | 5 | Undefined | Undefined | -1.08930 | 0.00172 |
| 117.1153 | 19.1401 | C6H15NO | 2-Methylcholine | 7 | Undefined | Undefined | 0.66755 | 0.00964 |
| 266.1126 | 12.2196 | C10H14N6O3 | 3'-amino-3'-deoxyadenosine | 5 | Undefined | Undefined | 0.88700 | 0.01274 |
| 143.0946 | 4.6638 | C7H13NO2 | 4-(Trimethylammonio)but-2-enoate | 7 | Undefined | Undefined | 0.78482 | 0.00558 |
| 266.0647 | 21.9465 | C10H10N4O5 | 5'-Oxoinosine | 5 | Undefined | Undefined | -1.18740 | 0.00002 |
| 161.0510 | 7.2031 | C6H11NO2S | allylcysteine | 5 | Undefined | alliin degradation __ isoalliin degradation | 2.61970 | 0.00000 |
| 220.0519 | 13.5547 | C7H12N2O4S | AMCC | 7 | Undefined | Undefined | -1.72470 | 0.00498 |
| 119.0946 | 12.9063 | C5H13NO2 | betaine aldehyde hydrate | 7 | Undefined | glycine betaine biosynthesis III (plants) | 2.44520 | 0.00000 |
| 260.0831 | 7.8045 | C10H16N2O4S | d-biotin d-sulfoxide | 5 | Undefined | Undefined | -0.67992 | 0.00857 |
| 244.1059 | 8.7335 | C10H16N2O5 | dihydrothymidine | 7 | Undefined | Undefined | -1.79890 | 0.00659 |
| 167.0253 | 10.6368 | C4H9NO4S | Homocysteinesulfinicacid | 5 | Undefined | Undefined | -2.44560 | 0.00000 |
| 176.1053 | 12.9454 | C8H16O4 | L-Cladinose | 5 | Undefined | Undefined | -2.48230 | 0.00000 |
| 228.1474 | 7.8000 | C11H20N2O3 | L-isoleucyl-L-proline | 7 | Undefined | Undefined | -1.28530 | 0.00296 |
| 133.0198 | 8.6936 | C4H7NO2S | L-thiazolidine-4-carboxylate | 7 | Undefined | Undefined | 1.27220 | 0.00288 |
| 414.2044 | 3.5324 | C24H30O6 | Magnoshinin | 7 | Undefined | Undefined | 3.42760 | 0.00046 |
| 213.1110 | 13.6555 | C9H15N3O3 | Methyl 2-diazoacetamidohexonate | 7 | Undefined | Undefined | -1.10360 | 0.00136 |
| 439.2357 | 8.3343 | C26H33NO5 | Militarinone B | 5 | Undefined | Undefined | -3.25330 | 0.00002 |
| 225.0791 | 21.9260 | C14H11NO2 | Murrayanine | 5 | Undefined | Undefined | -0.72017 | 0.00417 |
| 285.0960 | 11.1441 | C11H15N3O6 | N4-Acetylcytidine | 5 | Undefined | Undefined | 0.95373 | 0.00102 |
| 205.0952 | 9.8011 | C8H15NO5 | N-Acetyl-D-fucosamine | 5 | Undefined | Undefined | -2.39850 | 0.00018 |
| 237.0850 | 13.2638 | C8H15NO7 | N-Acetyl-D-glucosaminate | 5 | Undefined | Undefined | ***-2.42020*** | 0.00783 |
| 129.1518 | 14.6571 | C8H19N | Octylamine | 7 | Undefined | Undefined | 2.09670 | 0.00331 |
| 214.0953 | 7.7962 | C9H14N2O4 | Pyrimidine nucleoside | 5 | Undefined | Undefined | -0.91803 | 0.00012 |
| 221.0358 | 14.0798 | C7H11NO5S | S-(3-oxo-3-carboxy-n-propyl)cysteine | 5 | Undefined | Undefined | -0.71790 | 0.00039 |
| 425.2198 | 9.7697 | C18H31N7O3S | S-adenosyl-1,8-diamino-3-thiooctane | 7 | Undefined | Undefined | -4.12950 | 0.00000 |
| 211.0670 | 9.0818 | C10H13NO2S | S-benzyl-D-cysteine | 7 | Undefined | Undefined | -3.43610 | 0.00000 |
| 143.0582 | 5.2834 | C6H9NO3 | Vinylacetylglycine | 7 | Undefined | Undefined | -0.73447 | 0.00320 |
| 134.0580 | 9.6077 | C5H10O4 | (R)-2,3-Dihydroxy-3-methylbutanoate | 6 | Undefined | Valine, leucine and isoleucine biosynthesis | ***-2.01060*** | 0.00008 |
| 148.0736 | 7.5611 | C6H12O4 | (R)-2,3-Dihydroxy-3-methylpentanoate | 8 | Undefined | Valine, leucine and isoleucine biosynthesis | 0.91051 | 0.00396 |
| 6 hr Metabolites | | | | | | | | |
| Mass | **RT** | **Formula** | **Putative metabolite** | **Confidence** | **MAP** | **Pathway** | **Log2-fold change** | **FDR adjusted *p*-value** |
| 134.0580 | 9.6077 | C5H10O4 | (R)-2,3-Dihydroxy-3-methylbutanoate | 6 | Amino Acid Metabolism | Valine, leucine and isoleucine biosynthesis | -0.92969 | 0.00225 |
| 113.0477 | 8.5001 | C5H7NO2 | (S)-1-Pyrroline-5-carboxylate | 8 | Amino Acid Metabolism | Glutamate metabolism__Arginine and proline metabolism | -0.95720 | 0.00071 |
| 132.0423 | 14.9690 | C5H8O4 | (S)-2-Acetolactate | 8 | Amino Acid Metabolism | Valine, leucine and isoleucine biosynthesis__C5-Branched dibasic acid metabolism | 0.78541 | 0.01090 |
| 180.0899 | 9.4283 | C9H12N2O2 | 3-Hydroxykynurenamine | 5 | Amino Acid Metabolism | Tryptophan metabolism | -0.86929 | 0.00084 |
| 156.0535 | 11.4999 | C6H8N2O3 | 4-Imidazolone-5-propanoate | 8 | Amino Acid Metabolism | Histidine metabolism | ***-0.68956*** | 0.00326 |
| 145.0375 | 8.9960 | C5H7NO4 | 4-Oxoglutaramate | 6 | Amino Acid Metabolism | Histidine metabolism | ***0.81674*** | 0.00030 |
| 324.0242 | 16.2085 | C10H13O10P | 5-O-(1-Carboxyvinyl)-3-phosphoshikimate | 6 | Amino Acid Metabolism | Phenylalanine, tyrosine and tryptophan biosynthesis | 3.84280 | 0.00018 |
| 103.0997 | 20.7356 | C5H13NO | Choline | 8 | Amino Acid Metabolism | Glycine, serine and threonine metabolism, Glycerophospholipid metabolism | 0.92033 | 0.00016 |
| 259.0457 | 16.3549 | C6H14NO8P | D-Glucosamine 6-phosphate | 8 | Amino Acid Metabolism | Glutamate metabolism, Aminosugars metabolism | ***0.98829*** | 0.00151 |
| 142.0742 | 12.5569 | C6H10N2O2 | Ectoine | 5 | Amino Acid Metabolism | Glycine, serine and threonine metabolism | -1.11810 | 0.00000 |
| 199.0959 | 11.5965 | C8H13N3O3 | gamma-Glutamyl-beta-aminopropiononitrile | 5 | Amino Acid Metabolism | Cyanoamino acid metabolism | -2.55680 | 0.00000 |
| 216.1108 | 8.5437 | C9H16N2O4 | gamma-Glutamyl-gamma-aminobutyraldehyde | 7 | Amino Acid Metabolism | Arginine and proline metabolism | -2.15930 | 0.00000 |
| 161.0688 | 9.6548 | C6H11NO4 | L-2-Aminoadipate | 8 | Amino Acid Metabolism | Lysine biosynthesis, Lysine degradation, Penicillin and cephalosporin biosynthesis | 1.01980 | 0.00217 |
| 89.0478 | 14.5914 | C3H7NO2 | L-Alanine | 8 | Amino Acid Metabolism | Alanine and aspartate metabolism, Cysteine metabolism, Taurine and hypotaurine metabolism, Selenoamino acid metabolism, D-Alanine metabolism, Carbon fixation, Reductive carboxylate cycle (CO2 fixation) | ***1.15530*** | 0.00002 |
| 175.0957 | 15.6479 | C6H13N3O3 | L-Citrulline | 8 | Amino Acid Metabolism | Arginine and proline metabolism | ***-1.65420*** | 0.00027 |
| 169.0046 | 16.3292 | C3H7NO5S | L-Cysteate | 8 | Amino Acid Metabolism | Cysteine metabolism, Taurine and hypotaurine metabolism | ***-2.44900*** | 0.00016 |
| 240.0240 | 16.2292 | C6H12N2O4S2 | L-Cystine | 5 | Amino Acid Metabolism | Cysteine metabolism | ***1.16030*** | 0.00022 |
| 146.0692 | 14.8369 | C5H10N2O3 | L-Glutamine | 8 | Amino Acid Metabolism | Glutamate metabolism, Purine metabolism, Pyrimidine metabolism, D-Glutamine and D-glutamate metabolism, Nitrogen metabolism | ***-0.75023*** | 0.00162 |
| 139.0745 | 7.4610 | C6H9N3O | L-Histidinal | 8 | Amino Acid Metabolism | Histidine metabolism | ***2.25040*** | 0.00957 |
| 155.0695 | 14.7300 | C6H9N3O2 | L-Histidine | 8 | Amino Acid Metabolism | Histidine metabolism, beta-Alanine metabolism | ***-0.91786*** | 0.00527 |
| 131.0946 | 10.7587 | C6H13NO2 | L-Leucine | 6 | Amino Acid Metabolism | Valine, leucine and isoleucine degradation, Valine, leucine and isoleucine biosynthesis | 0.65902 | 0.00078 |
| 105.0427 | 15.7536 | C3H7NO3 | L-Serine | 8 | Amino Acid Metabolism | Glycine, serine and threonine metabolism, Methionine metabolism, Cysteine metabolism, Cyanoamino acid metabolism, Sphingolipid metabolism, Methane metabolism, Sulfur metabolism | ***0.98828*** | 0.00102 |
| 119.0582 | 14.7210 | C4H9NO3 | L-Threonine | 8 | Amino Acid Metabolism | Glycine, serine and threonine metabolism, Valine, leucine and isoleucine biosynthesis, Porphyrin and chlorophyll metabolism | 1.61490 | 0.00002 |
| 290.1229 | 16.6755 | C10H18N4O6 | N-(L-Arginino)succinate | 8 | Amino Acid Metabolism | Arginine and proline metabolism, Alanine and aspartate metabolism | -0.91550 | 0.00029 |
| 232.1058 | 8.8266 | C9H16N2O5 | N2-Succinyl-L-ornithine | 6 | Amino Acid Metabolism | Arginine and proline metabolism | 0.85708 | 0.01127 |
| 217.1062 | 11.0140 | C8H15N3O4 | N-Acetyl-L-citrulline | 6 | Amino Acid Metabolism | arginine biosynthesis III | ***-2.12880*** | 0.00032 |
| 189.0637 | 14.4359 | C7H11NO5 | N-Acetyl-L-glutamate | 8 | Amino Acid Metabolism | Arginine and proline metabolism | ***1.03410*** | 0.01159 |
| 174.1005 | 9.7524 | C7H14N2O3 | N-Acetylornithine | 8 | Amino Acid Metabolism | Arginine and proline metabolism | -0.58832 | 0.00170 |
| 130.1106 | 7.1747 | C6H14N2O | N-Acetylputrescine | 6 | Amino Acid Metabolism | Arginine and proline metabolism | -0.80477 | 0.01099 |
| 174.0640 | 14.6705 | C6H10N2O4 | N-Formimino-L-glutamate | 8 | Amino Acid Metabolism | Histidine metabolism | ***1.28240*** | 0.00014 |
| 177.0459 | 8.3939 | C6H11NO3S | N-Formyl-L-methionine | 7 | Amino Acid Metabolism | Methionine metabolism | ***-1.86350*** | 0.00107 |
| 219.0740 | 14.1218 | C8H13NO6 | O-Succinyl-L-homoserine | 8 | Amino Acid Metabolism | Methionine metabolism, Sulfur metabolism | 1.44860 | 0.00351 |
| 219.1106 | 8.6430 | C9H17NO5 | Pantothenate | 8 | Amino Acid Metabolism | beta-Alanine metabolism, Pantothenate and CoA biosynthesis | ***1.16200*** | 0.00020 |
| 164.0474 | 4.6798 | C9H8O3 | Phenylpyruvate | 8 | Amino Acid Metabolism | Phenylalanine metabolism, Phenylalanine, tyrosine and tryptophan biosynthesis | 2.48440 | 0.00289 |
| 197.1164 | 11.5505 | C9H15N3O2 | Hercynine | 5 | Amino Acid Metabolism | Histidine metabolism | ***-1.30340*** | 0.00001 |
| 398.0997 | 17.4363 | C21H18O8 | Dihydro-NAME | 7 | Biosynthesis of Polyketides and Nonribosomal Peptides | Biosynthesis of type II polyketide products | -1.46510 | 0.00069 |
| 155.0582 | 8.8894 | C7H9NO3 | (3S,5R)-carbapenam | 7 | Biosynthesis of Secondary Metabolites | (5R)-carbapenem biosynthesis | -1.11150 | 0.00020 |
| 90.0317 | 10.0198 | C3H6O3 | (S)-Lactate | 8 | Carbohydrate Metabolism | Glycolysis / Gluconeogenesis, Pyruvate metabolism, Propanoate metabolism, Styrene degradation | 0.97327 | 0.00109 |
| 195.0743 | 16.9602 | C6H13NO6 | 2-Amino-2-deoxy-D-gluconate | 5 | Carbohydrate Metabolism | Pentose phosphate pathway, Aminosugars metabolism | 0.89365 | 0.00602 |
| 177.0637 | 7.8317 | C6H11NO5 | 4-Hydroxy-4-methylglutamate | 7 | Carbohydrate Metabolism | C5-Branched dibasic acid metabolism | 1.00970 | 0.00021 |
| 120.0423 | 12.2572 | C4H8O4 | D-Erythrose | 5 | Carbohydrate Metabolism | Undefined | 0.66448 | 0.00002 |
| 178.0478 | 12.3723 | C6H10O6 | D-Glucono-1,5-lactone | 8 | Carbohydrate Metabolism | Pentose phosphate pathway | ***-0.78664*** | 0.00465 |
| 179.0794 | 16.2658 | C6H13NO5 | D-Glucosamine | 5 | Carbohydrate Metabolism | Aminosugars metabolism | ***-0.82613*** | 0.00007 |
| 260.0297 | 16.2538 | C6H13O9P | D-Glucose 6-phosphate | 6 | Carbohydrate Metabolism | Starch and sucrose metabolism, Streptomycin biosynthesis, Inositol phosphate metabolism | ***0.98829*** | 0.00023 |
| 240.0847 | 15.7413 | C8H16O8 | D-glycero-L-galacto-Octulose | 7 | Carbohydrate Metabolism | Undefined | ***-0.82613*** | 0.00000 |
| 232.0348 | 15.6721 | C5H13O8P | D-Ribitol 5-phosphate | 7 | Carbohydrate Metabolism | Pentose and glucuronate interconversions | ***1.67390*** | 0.00007 |
| 230.0190 | 15.9172 | C5H11O8P | D-Ribose 5-phosphate | 6 | Carbohydrate Metabolism | Pentose phosphate pathway, Purine metabolism, Carbon fixation | ***0.68100*** | 0.00761 |
| 290.0404 | 16.3305 | C7H15O10P | D-Sedoheptulose 7-phosphate | 6 | Carbohydrate Metabolism | Pentose phosphate pathway, Carbon fixation | ***0.95417*** | 0.00041 |
| 152.0684 | 12.8558 | C5H12O5 | L-Arabitol | 8 | Carbohydrate Metabolism | Pentose and glucuronate interconversions | 1.89670 | 0.00000 |
| 666.2223 | 17.3376 | C24H42O21 | Maltotetraose | 8 | Carbohydrate Metabolism | glycogen degradation I | 1.58490 | 0.00005 |
| 504.1696 | 16.3168 | C18H32O16 | Maltotriose | 6 | Carbohydrate Metabolism | glycogen degradation I | 1.20530 | 0.00021 |
| 309.1062 | 13.4556 | C11H19NO9 | N-Acetylneuraminate | 6 | Carbohydrate Metabolism | Aminosugars metabolism | ***1.13560*** | 0.00525 |
| 154.0031 | 12.0185 | C3H7O5P | Propanoyl phosphate | 8 | Carbohydrate Metabolism | Propanoate metabolism, C5-Branched dibasic acid metabolism | 1.42800 | 0.00007 |
| 88.0160 | 8.7945 | C3H4O3 | Pyruvate | 8 | Carbohydrate Metabolism | Glycolysis / Gluconeogenesis, Citrate cycle (TCA cycle), Pentose phosphate pathway, Ascorbate and aldarate metabolism, Biosynthesis of steroids, Alanine and aspartate metabolism, Glycine, serine and threonine metabolism | ***0.88098*** | 0.00051 |
| 598.3574 | 4.6510 | C28H54O13 | Sucrose monopalmitate | 7 | Carbohydrate Metabolism | Undefined | -0.58705 | 0.00268 |
| 566.0552 | 16.6734 | C15H24N2O17P2 | UDP-glucose | 6 | Carbohydrate Metabolism | Pentose and glucuronate interconversions, Galactose metabolism, Ascorbate and aldarate metabolism, Pyrimidine metabolism, Starch and sucrose metabolism, Nucleotide sugars metabolism, Glycerolipid metabolism, Zeatin biosynthesis, Biosynthesis of ansamycins | ***1.81310*** | 0.00002 |
| 238.0690 | 9.8676 | C8H14O8 | 3-Deoxy-D-manno-octulosonate | 8 | Energy Metabolism | Lipopolysaccharide biosynthesis | -1.18340 | 0.00037 |
| 574.6761 | 15.4063 | C40H65N9O26P2 | UDPMurAc(oyl-L-Ala-D-gamma-Glu-L-Lys-D-Ala-D-Ala) | 8 | Glycan Biosynthesis and Metabolism | Peptidoglycan biosynthesis | ***1.25120*** | 0.00184 |
| 183.0662 | 14.6590 | C5H14NO4P | Choline phosphate | 8 | Lipid Metabolism | Glycerophospholipid metabolism, Glycine, serine and threonine metabolism | ***-1.86250*** | 0.00000 |
| 104.0474 | 8.4394 | C4H8O3 | (R)-3-Hydroxybutanoate | 8 | Lipid Metabolism | Synthesis and degradation of ketone bodies, Butanoate metabolism | ***-1.06740*** | 0.00136 |
| 257.1028 | 13.7581 | C8H20NO6P | sn-glycero-3-Phosphocholine | 8 | Lipid Metabolism | Glycerophospholipid metabolism, Ether lipid metabolism | -0.68798 | 0.00653 |
| 324.3030 | 3.2225 | C21H40O2 | [FA (21:2)] octadecenoic Acid | 5 | Lipids: Fatty Acyls | Fatty Acids and Conjugates | ***-1.09780*** | 0.00483 |
| 200.1776 | 3.5230 | C12H24O2 | Dodecanoic acid | 7 | Lipids: Fatty Acyls | Fatty acid biosynthesis | -0.71972 | 0.00511 |
| 200.1051 | 7.0974 | C10H16O4 | FA (12:1) Decenedioic acid | 5 | Lipids: Fatty Acyls | Fatty Acids and Conjugates | 0.89442 | 0.00303 |
| 226.1928 | 3.5122 | C14H26O2 | FA (14:1) tetradecenoic acid | 5 | Lipids: Fatty Acyls | Fatty Acids and Conjugates | -0.76716 | 0.00211 |
| 312.3030 | 3.2272 | C20H40O2 | FA (20:0) | 5 | Lipids: Fatty Acyls | Fatty Acids and Conjugates | 0.98976 | 0.00642 |
| 187.0845 | 4.6807 | C8H13NO4 | FA hydroxy(4:0) N-(3S-hydroxy-butanoyl)-homoserine lactone | 8 | Lipids: Fatty Acyls | Fatty amides | ***1.08080*** | 0.00015 |
| 160.1100 | 7.1169 | C8H16O3 | FA hydroxy(8:0) hydroxy-octanoic acid | 7 | Lipids: Fatty Acyls | Fatty Acids and Conjugates | ***1.07770*** | 0.00357 |
| 298.2872 | 3.2888 | C19H38O2 | FA methyl(18:0) | 5 | Lipids: Fatty Acyls | Fatty Acids and Conjugates | ***-1.08930*** | 0.00293 |
| 353.3293 | 3.3768 | C22H43NO2 | N-(11Z-eicosaenoyl)-ethanolamine | 5 | Lipids: Fatty Acyls | Fatty amides | 1.91950 | 0.01104 |
| 666.4474 | 3.1386 | C34H67O10P | [PG (28:0)] | 5 | Lipids: Glycerophospholipids | Glycerophosphoglycerols | 0.82317 | 0.00362 |
| 521.3489 | 4.0759 | C26H52NO7P | LysoPC(18:1) | 7 | Lipids: Glycerophospholipids | Glycerophosphocholines | ***2.46610*** | 0.00000 |
| 424.2595 | 3.8603 | C20H41O7P | PA(17:0/0:0) | 5 | Lipids: Glycerophospholipids | Glycerophosphates | 0.83488 | 0.00136 |
| 498.2963 | 3.4042 | C23H47O9P | PG(17:0) | 5 | Lipids: Glycerophospholipids | Glycerophosphoglycerols | 0.60070 | 0.00638 |
| 762.4466 | 28.2974 | C42H67O10P | PG(18:4(6Z,9Z,12Z,15Z)/18:4(6Z,9Z,12Z,15Z)) | 7 | Lipids: Glycerophospholipids | Glycerophosphoglycerols | 0.75096 | 0.00718 |
| 624.4003 | 3.1600 | C31H61O10P | PG(25:0) | 7 | Lipids: Glycerophospholipids | Glycerophosphoglycerols | 0.65504 | 0.00085 |
| 680.4633 | 3.1251 | C35H69O10P | PG(29:0) | 5 | Lipids: Glycerophospholipids | Glycerophosphoglycerols | 0.62511 | 0.00397 |
| 694.4788 | 3.1206 | C36H71O10P | PG(30:0) | 5 | Lipids: Glycerophospholipids | Glycerophosphoglycerols | ***1.04780*** | 0.00002 |
| 832.5077 | 12.0140 | C43H77O13P | PI(34:3) | 5 | Lipids: Glycerophospholipids | Glycerophosphoinositols | ***2.65780*** | 0.00000 |
| 848.4612 | 4.7338 | C42H74O13P2 | PGP(36:5) | 7 | Lipids: Glycerophospholipids | Undefined | ***1.07930*** | 0.00013 |
| 506.1787 | 16.1202 | C25H30O11 | [Fv Tetramethoxy(4:0)] 4_6_2'_4'-Tetramethoxychalcone 2'-beta-glucoside | 5 | Lipids: Polyketides | Flavonoids | 0.97718 | 0.00279 |
| 424.1154 | 12.4770 | C23H20O8 | 6-Hydroxy-6a,12a-dehydro-alpha-toxicarol | 7 | Lipids: Polyketides | Flavonoids | -1.55680 | 0.00597 |
| 195.0757 | 9.2198 | C7H9N5O2 | 2-Amino-4-hydroxy-6-hydroxymethyl-7,8-dihydropteridine | 8 | Metabolism of Cofactors and Vitamins | Folate biosynthesis | 1.87320 | 0.00980 |
| 246.1037 | 8.6823 | C10H18N2O3S | 9-mercaptodethiobiotin | 7 | Metabolism of Cofactors and Vitamins | biotin biosynthesis II | -0.86334 | 0.00757 |
| 278.1267 | 10.3730 | C14H18N2O4 | alpha-Ribazole | 8 | Metabolism of Cofactors and Vitamins | Riboflavin metabolism, Porphyrin and chlorophyll metabolism | -2.04110 | 0.00011 |
| 226.0478 | 15.8936 | C10H10O6 | Chorismate | 6 | Metabolism of Cofactors and Vitamins | Ubiquinone biosynthesis, Phenylalanine, tyrosine and tryptophan biosynthesis, Folate biosynthesis, Biosynthesis of siderophore group nonribosomal peptides | 2.34220 | 0.00001 |
| 214.1317 | 8.9268 | C10H18N2O3 | Dethiobiotin | 8 | Metabolism of Cofactors and Vitamins | Biotin metabolism | -2.38260 | 0.00000 |
| 131.0219 | 9.2559 | C4H5NO4 | Iminoaspartate | 8 | Metabolism of Cofactors and Vitamins | Nicotinate and nicotinamide metabolism | 0.77532 | 0.00633 |
| 115.0269 | 7.2352 | C4H5NO3 | Maleamate | 7 | Metabolism of Cofactors and Vitamins | Nicotinate and nicotinamide metabolism | 0.89463 | 0.00008 |
| 322.1199 | 7.3870 | C12H22N2O6S | N-((R)-Pantothenoyl)-L-cysteine | 7 | Metabolism of Cofactors and Vitamins | Pantothenate and CoA biosynthesis | -2.60450 | 0.00000 |
| 123.0321 | 7.8712 | C6H5NO2 | Nicotinate | 6 | Metabolism of Cofactors and Vitamins | Nicotinate and nicotinamide metabolism, Alkaloid biosynthesis II | 1.26150 | 0.00421 |
| 254.0898 | 24.3403 | C11H14N2O5 | N-Ribosylnicotinamide | 8 | Metabolism of Cofactors and Vitamins | Nicotinate and nicotinamide metabolism | 0.80211 | 0.00026 |
| 278.1298 | 4.9319 | C11H22N2O4S | Pantetheine | 7 | Metabolism of Cofactors and Vitamins | Pantothenate and CoA biosynthesis | ***1.06180*** | 0.00163 |
| 376.1382 | 7.8162 | C17H20N4O6 | Riboflavin | 6 | Metabolism of Cofactors and Vitamins | Riboflavin metabolism | 0.76058 | 0.00863 |
| 128.0585 | 13.7965 | C5H8N2O2 | 5,6-Dihydrothymine | 8 | Nucleotide Metabolism | Pyrimidine metabolism | -0.67793 | 0.00008 |
| 314.0517 | 15.2120 | C8H15N2O9P | 5'-Phosphoribosyl-N-formylglycinamide | 8 | Nucleotide Metabolism | Purine metabolism | ***3.70870*** | 0.00049 |
| 135.0545 | 8.5389 | C5H5N5 | Adenine | 8 | Nucleotide Metabolism | Purine metabolism, Zeatin biosynthesis | 0.61540 | 0.00429 |
| 267.0966 | 8.4193 | C10H13N5O4 | Adenosine | 8 | Nucleotide Metabolism | Purine metabolism | 0.77893 | 0.00707 |
| 243.0856 | 11.6453 | C9H13N3O5 | Cytidine | 7 | Nucleotide Metabolism | Pyrimidine metabolism | 0.91651 | 0.01197 |
| 387.0236 | 16.2051 | C9H15N3O10P2 | dCDP | 6 | Nucleotide Metabolism | Pyrimidine metabolism | ***-1.04520*** | 0.00037 |
| 151.0495 | 12.1702 | C5H5N5O | Guanine | 8 | Nucleotide Metabolism | Purine metabolism | ***1.47550*** | 0.00017 |
| 283.0918 | 12.4403 | C10H13N5O5 | Guanosine | 8 | Nucleotide Metabolism | Purine metabolism | ***1.03520*** | 0.00070 |
| 176.0433 | 17.3792 | C5H8N2O5 | N-Carbamoyl-L-aspartate | 8 | Nucleotide Metabolism | Pyrimidine metabolism, Alanine and aspartate metabolism | ***-2.20010*** | 0.00000 |
| 102.0430 | 12.1111 | C3H6N2O2 | N-Formiminoglycine | 5 | Nucleotide Metabolism | Purine metabolism | ***2.68620*** | 0.00000 |
| 156.0171 | 10.8314 | C5H4N2O4 | Orotate | 8 | Nucleotide Metabolism | Pyrimidine metabolism | ***-1.99530*** | 0.01040 |
| 126.0429 | 7.0906 | C5H6N2O2 | Thymine | 6 | Nucleotide Metabolism | Pyrimidine metabolism | -0.82674 | 0.00079 |
| 244.0696 | 9.6448 | C9H12N2O6 | Uridine | 5 | Nucleotide Metabolism | Pyrimidine metabolism | ***1.63340*** | 0.00079 |
| 192.0568 | 10.4210 | C6H12N2O3S | Ala-Cys | 7 | Peptide(di-) | Nonpolar peptide | -2.50500 | 0.00000 |
| 186.1005 | 11.6335 | C8H14N2O3 | Ala-Pro | 5 | Peptide(di-) | Nonpolar peptide | -2.37660 | 0.00000 |
| 229.1064 | 11.2655 | C9H15N3O4 | Asn-Pro | 7 | Peptide(di-) | Polar peptide | -0.81219 | 0.00074 |
| 218.0725 | 7.8703 | C8H14N2O3S | Cys-Pro | 7 | Peptide(di-) | Nonpolar peptide | 0.69217 | 0.00056 |
| 276.0957 | 14.1527 | C10H16N2O7 | Glu-Glu | 7 | Peptide(di-) | Acidic peptide | 2.42640 | 0.00000 |
| 243.1583 | 16.5156 | C11H21N3O3 | Lys-Pro | 7 | Peptide(di-) | Basic peptide | -1.30640 | 0.00018 |
| 345.1649 | 15.0513 | C13H23N5O6 | Ala-Ala-Ala-Asn | 5 | Peptide(tetra-) | Polar peptide | -1.03270 | 0.00048 |
| 331.1494 | 15.3126 | C12H21N5O6 | Ala-Ala-Asn-Gly | 5 | Peptide(tetra-) | Polar peptide | -1.33170 | 0.00000 |
| 358.1489 | 9.7958 | C14H22N4O7 | Ala-Asp-Gly-Pro | 7 | Peptide(tetra-) | Acidic peptide | -0.99995 | 0.00328 |
| 411.2120 | 12.8459 | C18H29N5O6 | Ala-Gln-Pro-Pro | 5 | Peptide(tetra-) | Polar peptide | -1.90430 | 0.00001 |
| 402.2120 | 9.4028 | C17H30N4O7 | Ala-Glu-Ala-Ile | 5 | Peptide(tetra-) | Hydrophobic peptide | -2.41900 | 0.00015 |
| 434.1839 | 12.4771 | C17H30N4O7S | Ala-Glu-Cys-Ile | 7 | Peptide(tetra-) | Hydrophobic peptide | -1.61010 | 0.00373 |
| 330.1908 | 13.0616 | C14H26N4O5 | Ala-Leu-Ala-Gly | 5 | Peptide(tetra-) | Hydrophobic peptide | -2.28460 | 0.00036 |
| 316.1745 | 14.4197 | C13H24N4O5 | Ala-Leu-Gly-Gly | 5 | Peptide(tetra-) | Hydrophobic peptide | -1.01280 | 0.00997 |
| 388.2319 | 9.7493 | C17H32N4O6 | Ala-Leu-Val-Ser | 5 | Peptide(tetra-) | Hydrophobic peptide | -2.35840 | 0.00000 |
| 462.1904 | 12.2269 | C17H30N6O7S | Ala-Met-Asn-Gln | 7 | Peptide(tetra-) | Hydrophobic peptide | -2.50110 | 0.00000 |
| 474.2223 | 8.1396 | C22H30N6O6 | Ala-Phe-Thr-His | 5 | Peptide(tetra-) | Basic peptide | -2.08400 | 0.01101 |
| 360.1645 | 11.6935 | C14H24N4O7 | Ala-Pro-Ser-Ser | 5 | Peptide(tetra-) | Polar peptide | -2.83670 | 0.00138 |
| 495.2443 | 8.4019 | C21H33N7O7 | Ala-Ser-Tyr-Arg | 7 | Peptide(tetra-) | Basic peptide | -1.55700 | 0.00182 |
| 342.1902 | 7.1169 | C15H26N4O5 | Ala-Val-Gly-Pro | 5 | Peptide(tetra-) | Hydrophobic peptide | 2.89470 | 0.00005 |
| 461.2055 | 4.1574 | C17H31N7O6S | Arg-Cys-Pro-Ser | 7 | Peptide(tetra-) | Basic peptide | -0.67918 | 0.00476 |
| 544.2970 | 12.3489 | C22H40N8O8 | Arg-Glu-Gln-Ile | 7 | Peptide(tetra-) | Basic peptide | 2.59060 | 0.00000 |
| 530.3164 | 10.7826 | C22H42N8O7 | Arg-Leu-Lys-Asp | 5 | Peptide(tetra-) | Basic peptide | -0.76477 | 0.01133 |
| 496.1909 | 10.5307 | C20H28N6O9 | Asn-Asn-Ser-Tyr | 7 | Peptide(tetra-) | Hydrophobic peptide | 2.50600 | 0.00000 |
| 538.2010 | 5.5198 | C22H30N6O10 | Asn-Asp-Gln-Tyr | 7 | Peptide(tetra-) | Hydrophobic peptide | 1.35050 | 0.00001 |
| 485.2237 | 8.4006 | C19H31N7O8 | Asn-Gln-Gln-Pro | 5 | Peptide(tetra-) | Polar peptide | 0.79736 | 0.00047 |
| 478.1850 | 8.4556 | C17H30N6O8S | Asn-Lys-Asp-Cys | 5 | Peptide(tetra-) | Basic peptide | 0.88300 | 0.00657 |
| 488.2056 | 8.5534 | C19H32N6O7S | Asn-Met-Gln-Pro | 7 | Peptide(tetra-) | Hydrophobic peptide | 0.81564 | 0.00253 |
| 529.2291 | 8.3032 | C24H31N7O7 | Asn-Pro-Tyr-His | 7 | Peptide(tetra-) | Basic peptide | -2.55860 | 0.00369 |
| 455.2005 | 10.9706 | C19H29N5O8 | Asp-Gln-Pro-Pro | 5 | Peptide(tetra-) | Acidic peptide | 1.16390 | 0.00012 |
| 458.2019 | 9.6201 | C19H30N4O9 | Asp-Leu-Asp-Pro | 5 | Peptide(tetra-) | Hydrophobic peptide | -2.83080 | 0.00001 |
| 529.1962 | 4.7447 | C20H31N7O8S | Asp-Met-Gln-His | 7 | Peptide(tetra-) | Basic peptide | 2.84320 | 0.00001 |
| 526.1603 | 14.4452 | C19H34N4O7S3 | Asp-Met-Met-Met | 5 | Peptide(tetra-) | Hydrophobic peptide | -2.47830 | 0.00270 |
| 581.1979 | 11.6407 | C25H35N5O7S2 | Asp-Met-Met-Trp | 5 | Peptide(tetra-) | Hydrophobic peptide | -0.93169 | 0.00004 |
| 480.1845 | 8.4533 | C21H28N4O9 | Asp-Pro-Ser-Tyr | 5 | Peptide(tetra-) | Hydrophobic peptide | 0.76326 | 0.00873 |
| 386.1805 | 11.6589 | C16H26N4O7 | Asp-Val-Gly-Pro | 5 | Peptide(tetra-) | Hydrophobic peptide | -3.82510 | 0.00034 |
| 540.1742 | 12.1832 | C20H36N4O7S3 | Glu-Met-Met-Met | 5 | Peptide(tetra-) | Hydrophobic peptide | 2.75050 | 0.00001 |
| 498.1635 | 12.1611 | C18H34N4O6S3 | Met-Met-Met-Ser | 5 | Peptide(tetra-) | Hydrophobic peptide | 1.81130 | 0.00001 |
| 366.1538 | 7.8837 | C16H22N4O6 | Ala-Asn-Tyr | 7 | Peptide(tri-) | Hydrophobic peptide | 2.45460 | 0.00000 |
| 243.1219 | 12.6568 | C10H17N3O4 | Ala-Gly-Pro | 7 | Peptide(tri-) | Polar peptide | -0.72483 | 0.00392 |
| 233.1010 | 9.2516 | C8H15N3O5 | Ala-Gly-Ser | 7 | Peptide(tri-) | Polar peptide | 0.68209 | 0.00726 |
| 317.1223 | 10.5451 | C12H19N3O7 | Asp-Pro-Ser | 5 | Peptide(tri-) | Acidic peptide | -0.58651 | 0.00090 |
| 347.1331 | 15.9972 | C13H21N3O8 | Glu-Ala-Glu | 5 | Peptide(tri-) | Acidic peptide | 1.12380 | 0.00088 |
| 331.1745 | 10.1428 | C14H25N3O6 | Glu-Ala-Ile | 7 | Peptide(tri-) | Hydrophobic peptide | 0.99476 | 0.00159 |
| 317.1589 | 14.9702 | C13H23N3O6 | Glu-Ala-Val | 7 | Peptide(tri-) | Hydrophobic peptide | 0.77060 | 0.00296 |
| 393.1208 | 15.5942 | C14H23N3O8S | Glu-Asp-Met | 7 | Peptide(tri-) | Hydrophobic peptide | -2.06200 | 0.00532 |
| 351.1431 | 10.6352 | C16H21N3O6 | Glu-Gly-Phe | 5 | Peptide(tri-) | Hydrophobic peptide | 0.94191 | 0.00005 |
| 359.2056 | 9.2561 | C16H29N3O6 | Glu-Ile-Val | 7 | Peptide(tri-) | Hydrophobic peptide | 0.64477 | 0.00562 |
| 344.2169 | 21.1969 | C14H28N6O4 | Ile-Arg-Gly | 5 | Peptide(tri-) | Basic peptide | -1.00980 | 0.00070 |
| 316.2112 | 20.5938 | C14H28N4O4 | Ile-Gly-Lys | 7 | Peptide(tri-) | Basic peptide | -0.93124 | 0.00836 |
| 319.1568 | 11.1613 | C13H25N3O4S | Ile-Gly-Met | 5 | Peptide(tri-) | Hydrophobic peptide | -1.31140 | 0.00003 |
| 305.1588 | 10.7173 | C12H23N3O6 | Ile-Ser-Ser | 5 | Peptide(tri-) | Hydrophobic peptide | -1.20560 | 0.00240 |
| 317.1698 | 13.8953 | C12H23N5O5 | Lys-Asn-Gly | 5 | Peptide(tri-) | Basic peptide | -1.09070 | 0.00048 |
| 334.1849 | 10.9269 | C13H26N4O6 | Lys-Thr-Ser | 7 | Peptide(tri-) | Basic peptide | -2.49250 | 0.00000 |
| 361.1309 | 12.8983 | C14H23N3O6S | Met-Asp-Pro | 5 | Peptide(tri-) | Hydrophobic peptide | -0.98294 | 0.00172 |
| 482.1978 | 14.4391 | C25H30N4O4S | Met-Phe-Trp | 5 | Peptide(tri-) | Hydrophobic peptide | -1.63140 | 0.00169 |
| 293.1223 | 14.8370 | C10H19N3O7 | Thr-Ser-Ser | 5 | Peptide(tri-) | Polar peptide | -1.67400 | 0.00002 |
| 307.1380 | 11.1069 | C11H21N3O7 | Thr-Thr-Ser | 5 | Peptide(tri-) | Polar peptide | -0.67081 | 0.00017 |
| 330.2017 | 21.2774 | C13H26N6O4 | Val-Gly-Arg | 7 | Peptide(tri-) | Basic peptide | -1.23890 | 0.00033 |
| 193.0409 | 8.2321 | C6H11NO4S | &gamma;-thiomethyl glutamate | 7 | Undefined | Undefined | -0.90642 | 0.00070 |
| 291.0954 | 12.5760 | C11H17NO8 | 2,7-Anhydro-alpha-N-acetylneuraminic acid | 5 | Undefined | Undefined | -0.95425 | 0.01067 |
| 383.2060 | 12.9247 | C20H31O7 | 20-Trihydroxy-leukotriene-B4 | 5 | Undefined | Undefined | -1.17800 | 0.00133 |
| 379.1487 | 12.3346 | C16H21N5O6 | 3,3-dimethyl-2-oxobutyric acid 5-(6-aminopurin-9-yl)-3,4-dihydroxytetrahydrofuran-2-ylmethyl ester | 7 | Undefined | Undefined | -0.73043 | 0.00317 |
| 498.1159 | 17.0238 | C25H22O11 | 3,4-Dicaffeoyl-1,5-quinolactone | 5 | Undefined | Undefined | -1.96740 | 0.00019 |
| 114.0793 | 14.2490 | C5H10N2O | 3-Amino-2-piperidone | 5 | Undefined | Undefined | -1.40830 | 0.00085 |
| 266.1126 | 12.2196 | C10H14N6O3 | 3'-amino-3'-deoxyadenosine | 5 | Undefined | Undefined | 1.25040 | 0.00005 |
| 220.0519 | 13.5547 | C7H12N2O4S | AMCC | 7 | Undefined | Undefined | -1.74210 | 0.01073 |
| 193.0586 | 13.2017 | C6H11NO6 | CPD0-1656 | 7 | Undefined | Undefined | 0.88192 | 0.00014 |
| 244.1059 | 8.7335 | C10H16N2O5 | dihydrothymidine | 7 | Undefined | Undefined | -1.58860 | 0.00047 |
| 157.1103 | 9.3663 | C8H15NO2 | Homostachydrine | 5 | Undefined | Undefined | -1.25790 | 0.00000 |
| 228.1474 | 7.8000 | C11H20N2O3 | L-isoleucyl-L-proline | 7 | Undefined | Undefined | -2.25890 | 0.00001 |
| 358.1114 | 15.6822 | C12H22O12 | melibionate | 7 | Undefined | Undefined | 0.89877 | 0.00079 |
| 439.2357 | 8.3343 | C26H33NO5 | Militarinone B | 5 | Undefined | Undefined | -1.69020 | 0.00125 |
| 191.0617 | 5.4806 | C7H13NO3S | N-Acetylmethionine | 7 | Undefined | Undefined | -0.80470 | 0.01028 |
| 293.1110 | 10.7185 | C11H19NO8 | N-Acetylmuramate | 8 | Undefined | Undefined | -0.59093 | 0.00323 |
| 425.2198 | 9.7697 | C18H31N7O3S | S-adenosyl-1,8-diamino-3-thiooctane | 7 | Undefined | Undefined | -1.96240 | 0.00011 |
| 211.0670 | 9.0818 | C10H13NO2S | S-benzyl-D-cysteine | 7 | Undefined | Undefined | -1.67750 | 0.00003 |
| 335.1581 | 7.1067 | C14H25NO8 | validoxylamine A | 5 | Undefined | validamycin A biosynthesis | -1.13520 | 0.00006 |
